# Adipogenic Activity of Chemicals Used in Plastic Consumer Products

**DOI:** 10.1021/acs.est.1c06316

**Published:** 2022-01-26

**Authors:** Johannes Völker, Felicity Ashcroft, Åsa Vedøy, Lisa Zimmermann, Martin Wagner

**Affiliations:** † Department of Biology, Norwegian University of Science and Technology (NTNU), 7491 Trondheim, Norway; ‡ Department of Aquatic Ecotoxicology, Goethe University Frankfurt am Main, 60438 Frankfurt am Main, Germany

**Keywords:** adipogenesis, endocrine-disrupting chemicals, metabolic disruptors, non-target chemical analysis, obesogens

## Abstract

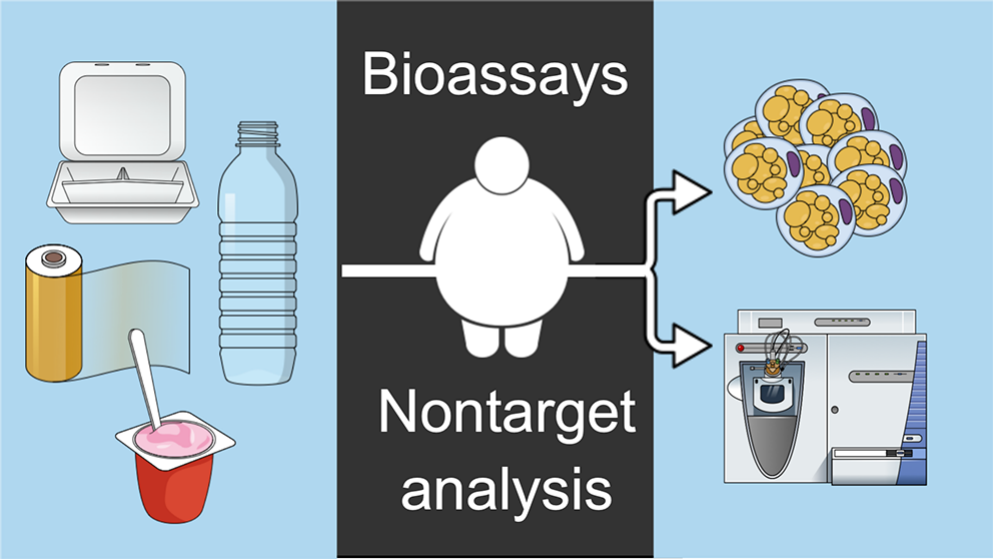

Bisphenols and phthalates, chemicals frequently used in plastic products, promote obesity in cell and animal models. However, these well-known metabolism-disrupting chemicals (MDCs) represent only a minute fraction of all compounds found in plastics. To gain a comprehensive understanding of plastics as a source of exposure to MDCs, we characterized the chemicals present in 34 everyday products using nontarget high-resolution mass spectrometry and analyzed their joint adipogenic activities by high-content imaging. We detected 55,300 chemical features and tentatively identified 629 unique compounds, including 11 known MDCs. Importantly, the chemicals extracted from one-third of the products caused murine 3T3-L1 preadipocytes to proliferate, and differentiate into adipocytes, which were larger and contained more triglycerides than those treated with the reference compound rosiglitazone. Because the majority of plastic extracts did not activate the peroxisome proliferator-activated receptor γ and the glucocorticoid receptor, the adipogenic effects are mediated via other mechanisms and, thus, likely to be caused by unknown MDCs. Our study demonstrates that daily-use plastics contain potent mixtures of MDCs and can, therefore, be a relevant yet underestimated environmental factor contributing to obesity.

## Abstract 2

Plastics contain a potent mixture of chemicals that promote adipogenesis, a key process in the development of obesity.

## 1 Introduction

The obesity pandemic generates a considerable burden of disease through comorbidities such as type 2 diabetes, cardiovascular disease, hypertension, nonalcoholic fatty liver disease, stroke, and certain types of cancer.^1^ The number of obese people worldwide has nearly tripled since 1975, and in 2016, more than 41 million children under the age of five were classified as being overweight or obese.^2^ This is problematic because a high body mass index (BMI) is one of the top risk factors for deaths,^3^ and overweight in childhood or adolescence is a good predictor of adult obesity.^4^ Accordingly, a high BMI and the associated comorbidities contributed to four million deaths globally in 2015 with cardiovascular diseases as the leading cause of death, followed by diabetes, chronic kidney diseases, and cancer.^5^

This public health problem has been largely attributed to genetic background and changes in lifestyle, such as diet, exercise, sleep deficiency, and aging. However, epidemiological evidence suggests that these factors are not sufficient to explain the magnitude and speed of the obesity pandemic’s spread.^6^ For instance, even after normalizing for caloric intake and exercise, the BMI of US adults increased by 2.3 kg m^–2^ between 1998 and 2006.^6^ Consequently, identifying and understanding other environmental factors than lifestyle is crucial to manage obesity.^7^ Given that the endocrine system controls appetite, satiety, metabolism, and weight, exposure to endocrine-disrupting chemicals is one such factor.^8^ Connecting endocrine disruption and obesity gave rise to the so-called obesogen hypothesis, which poses that environmental chemicals (obesogens) contribute to obesity by direct (e.g., promotion of adipocyte commitment, differentiation, and growth) and indirect mechanisms (e.g., change in metabolic setpoints).^9,10^ When this hypothesis was expanded to include other metabolic disorders, such as type 2 diabetes, the term metabolism-disrupting chemical (MDC) was adopted. Many obesogens are endocrine-disrupting chemicals that interfere with normal endocrine regulation. Prominent endocrine disruptors, such as the biocide tributyltin and the pesticide dichlorodiphenyltrichloroethane (DDT), and plastic chemicals, such as bisphenols and phthalates, disrupt metabolic functions or promote obesity in cell and animal experiments.^8^ This is further supported by epidemiological studies that have linked weight gain in humans to bisphenol A (BPA) exposure,^11^ while contradicting outcomes have been reported regarding a link to phthalate exposure.^12−14^ Nonetheless, recent longitudinal studies report a positive association between early-life exposures to certain phthalates and obesity.^15,16^

Considering the chemical complexity of plastic consumer products, bisphenols and phthalates represent only the tip of the iceberg. A final article often consists of one or more polymers, multiple intentionally added substances, such as fillers or additives, as well as nonintentionally added substances, for instance, residues from the manufacturing.^17^ Based on regulatory inventories, over 4000 substances are associated with plastic food packaging alone,^17^ and 10,547 chemicals are known to be used in plastics.^18^ Moreover, empirical data suggest that plastics contain more chemicals than currently known. For instance, using nontarget chemical analysis, we detected hundreds to thousands of chemicals in plastic consumer products, most of which remain unknown.^19^ Importantly, the totality of plastic chemicals in a product was toxic in vitro, inducing baseline toxicity, oxidative stress, cytotoxicity, and endocrine effects.

Building on these results and the fact that bisphenols and phthalates are known MDCs,^8,9,20^ we hypothesized that MDCs are present in plastic consumer products and that metabolic disruption might represent a common but understudied toxicological property of plastic chemicals. We decided to study all extractable chemicals from plastic products instead of individual, well-known compounds, to avoid issues associated with an a priori selection (e.g., confirmation bias) as well as to cover unknown chemicals and mixture effects. Thus, we used the same plastic consumer products we have extensively characterized previously^19^ and investigated the extracts’ adipogenic activity in murine 3T3-L1 cells. Following exposure to MDCs, 3T3-L1 pre-adipocytes differentiate into adipocytes and accumulate triglycerides until they finally resemble mature white fat cells.^21^ The bioassay targets the induction of adipogenesis at the cellular level and represents a well-established in vitro model for metabolic disruption in vivo.^22^ We performed optimization experiments and applied high-content fluorescence microscopy combined with automated image processing to increase the sensitivity and throughput of the assay. We also investigated the underlying mechanism of the adipogenic response by testing whether the extracted plastic chemicals activate the human peroxisome proliferator-activated receptor gamma (PPARγ) or glucocorticoid receptor (GR). We selected PPARγ as a key regulator of adipogenesis^23^ and included GR because glucocorticoids are important regulators of lipid metabolism.^24^ Accordingly, an excess of agonists for these receptors is associated with obesogenic effects in animal models and humans (e.g., weight gain).^22^ Moreover, we performed nontarget, ultrahigh performance liquid chromatography coupled to a quadrupole of flight spectrometer (LC-QTOF-MS/MS) to characterize the chemicals present in plastics and compared these with compounds known to induce adipogenesis.

## 2 Materials and Methods

A list of used chemicals is provided in the Supporting Information (Table S1).

### 2.1 Sample Selection and Plastic Extraction

We used the same 34 plastic samples (Table 1) as in Zimmermann et al.^19^ The samples cover petroleum-based polymer types with the highest market share (polypropylene (PP) > low density polyethylene (LDPE) > high density polyethylene (HDPE) > polyvinyl chloride (PVC) > polyurethane (PUR) > polyethylene terephthalate (PET) > polystyrene (PS)),^25^ and polylactic acid (PLA) as a biobased alternative. The samples include 21 products with and 13 products without food contact. Further specifications on the sample selection, collection, and polymer identification are described by Zimmermann et al.^19^ We extracted 3 g of the sample, including three procedure blanks (PB 1–3), with methanol and concentrated the extracts to a final volume of 200 μL using dimethyl sulfoxide as a keeper. We used methanol as a solvent because it extracts rather polar compounds (i.e., chemicals that may also migrate into water), while it does not dissolve the polymers we analyzed. In addition, the application of the same method as in our previous work allowed us to reuse the analytical data to screen for MDCs. To contextualize the bioassay results, we use “plastic equivalents” such that “1 mg plastic” corresponds to the chemicals extracted from 1 mg of plastic. Accordingly, 1 μL of extract corresponds to 15 mg of plastic. See the Supporting Information (plastic extraction) for details.

**Table 1:** Plastic Products Analyzed in this Study, Results of the Nontarget Chemical Analysis, and the Tentatively Identified MDCs

sample	plastic product	LC-QTOF-MS/MS (number of features)				tentatively identified MDCs
		in sample	with MS2	ID score ≥ 40	% of MS2	
HDPE 1	refillable drinking bottle^a^	779	203	38	18.7	TPP
HDPE 2	yogurt drinking bottle^a^	107	34	7	20.6	
HDPE 3	bin liner	614	153	30	19.6	TPP
HDPE 4	shower gel bottle	164	50	16	32.0	EHDP
LDPE 1	lemon juice bottle^a^	241	66	20	30.3	EHDP
LDPE 2	plastic wrap^a^	1833	543	98	18.0	TPP
LDPE 3	freezer bag^a^	1603	416	62	14.9	TPP
LDPE 4	hair conditioner bottle	1702	544	89	16.4	allethrin, TPP
PS 1	yogurt cup^a^	447	96	12	12.5	TPP
PS 2	fruit tray^a^	1122	293	44	15.0	DPP, TPP
PS 3	vegetable tray^a^	308	63	11	17.5	
PS 4	plastic cup^a^	119	30	7	23.3	
PP 1	refillable drinking bottle^a^	1365	396	87	22.0	TPP
PP 2	yogurt cup^a^	1870	549	93	16.9	TPP
PP 3	gummy candy packaging^a^	3159	910	117	12.9	TPP
PP 4	handkerchief packaging	1798	519	85	16.4	TPP
PP 5	shampoo bottle	268	101	29	28.7	
PET 1	soft drink bottle^a^	148	55	18	32.7	
PET 2	yogurt cup^a^	179	51	12	23.5	
PET 3	oven bag^a^	647	159	30	18.9	
PET 4	vegetable tray^a^	695	182	20	11.0	
PET 5	shampoo bottle	375	89	11	12.4	
PVC 1	plastic wrap^a^	3655	1374	118	8.6	
PVC 2	place mat	2426	819	145	17.7	DPP, TPP
PVC 3	pond liner	1270	450	91	20.2	DINP, TPP
PVC 4	floor covering	2361	868	145	16.7	BBP, BPDP, DBP, DEHP, DINP, DPP, EHDP, TBEP, TOCP, TPP
PUR 1	scouring pad	5619	1773	216	12.2	EHDP, TPP
PUR 2	kids bath sponge	4521	1182	151	12.8	
PUR 3	acoustic foam	6242	2117	224	10.6	EHDP, TPP
PUR 4	shower slippers	1035	300	78	26.0	EHDP, TPP
PLA 1	yogurt cup^a^	2421	772	52	6.7	TPP
PLA 2	vegetable tray^a^	1983	672	40	6.0	
PLA 3	coffee cup lid^a^	N/A	N/A	N/A	N/A	
PLA 4	coffee cup lid^a^	2575	857	73	8.5	

a FCM = food contact material, BBP = benzyl butyl phthalate, BPDP = tert-butylphenyl diphenyl phosphate, DBP = dibutyl phthalate, DEHP = bis(2-ethylhexyl) phthalate, DINP = di-iso-nonyl phthalate, DPP = diphenyl phosphate, EHDP = 2-ethylhexyl diphenyl phosphate, N/A = not analyzed, TBEP = tris(2 butoxyethyl) phosphate, TOCP = tri-o-cresyl phosphate, and TPP = triphenyl phosphate.

### 2.2 Bioassays

We performed differentiation assays with murine 3T3-L1 adipocytes (Zenbio Inc., SP-L1-F, lot 3T3L1062104) to examine the induction of adipogenesis, and used CALUX reporter gene assays (BioDetection Systems B.V., Amsterdam, The Netherlands) to investigate the agonistic activity at the human peroxisome proliferator-activated receptor γ (PPARγ)^26^ and the glucocorticoid receptor (GR).^27^ All experiments were conducted with negative controls, vehicle controls, positive controls, and PB 1–3. Samples, controls, and blanks were diluted 1000-fold (adipogenesis assay) or 500-fold (reporter gene assays) with medium, resulting in a maximum final solvent concentration of 0.1 or 0.2% (v/v), respectively. Each sample was analyzed in serial dilutions of 1:2 with four replicates per concentration in at least three independent experiments per assay. Moreover, the respective reference compound was included on every microtiter plate to control for potential variations between plates, and the sample arrangement was randomized to exclude position effects. As negative controls and vehicle controls did not differ significantly, the results from both controls were pooled. Furthermore, there was no contamination during sample extraction and analysis because none of the controls and blanks induced activity (Figures S1 and S2). Details on the cell culture conditions can be found in the Supporting Information (cell culture conditions).

### 2.3 Adipogenesis Assay

We performed the differentiation assays with 3T3-L1 cells in accordance with a previously described method.^28^ In brief, an experiment consists of 3 days predifferentiation (1 day seeding and 2 days to allow cells to enter the resting state), followed by an 8 days differentiation window (2 days induction of differentiation and 6 days maintenance). Subconfluent cells of passage 10 were trypsinized and counted with a flow cytometer (NovoCyte, Acea Biosciences). 15,000 cells well^–1^ were seeded in 200 μL of preadipocyte medium (PAM: DMEM-high supplemented with 10% bovine calf serum and 1% penicillin/streptomycin) into 96-well black, clear-bottom tissue culture plates (655,090, Greiner Bio-One) and incubated at 37 °C and 5% CO_2_. After 24 h, we checked that the cells had reached confluency, replaced the medium with 200 μL fresh PAM well^–1^, and cultured the cells for another 48 h to initiate growth arrest. We included a preadipocyte control (undifferentiated cells) on every plate which we kept cultivated in PAM, while the rest of the cells were differentiated as described below.

### 2.4 Optimization Experiments

Given that a systematic analysis of dexamethasone (DEX) effects on triglyceride accumulation and differentiation efficiency in 3T3-L1 cells was lacking, we conducted optimization experiments to identify a suitable DEX concentration to initiate adipocyte differentiation that results in the lowest baseline as well as the highest sensitivity and dynamic range when coexposed to the reference compound rosiglitazone. Moreover, we compared two methods to quantify triglycerides based on NileRed staining. We determined the total NileRed fluorescence well^–1^ and compared it to an automated imaging and analysis platform to determine whether the latter improves the sensitivity and dynamic range for screening adipogenic activity.

Based on the results (Figure S3) and in comparison with previous studies, we found that a rather low DEX concentration (6.25 nM) was optimal to initiate adipocyte differentiation without increasing the assay’s baseline. Compared to the fluorescence readout well^–1^, the automated imaging approach was more sensitive to measure proliferation, enhanced the dynamic range of the assay (Figure S3), and provided more information by enabling single-cell analysis and, therefore, a more detailed characterization of the adipocyte population (pre-adipocytes, adipocytes, and mature adipocytes). Accordingly, we analyzed the effects of the plastic extracts using 6.25 nM DEX during the differentiation window and the automated imaging approach. See the Supporting Information (optimization of the adipogenesis assay) for details.

### 2.5 Dosing of Samples

To initiate differentiation, we replaced the PAM medium with 200 μL of differentiation medium well^–1^ (DM: DMEM-high supplemented with 10% FBS, 1% penicillin/streptomycin, 20 mM HEPES, 1 μg mL^–1^ insulin, 0.5 mM 3-isobutyl-1-methylxanthine (IBMX), and 6.25 nM DEX) containing five concentrations of samples serially diluted 1:2 (0.19–3 mg plastic well^–1^ equivalent to 0.94–15 mg plastic mL^–1^) or rosiglitazone (1.17–300 nM). After 48 h, the medium was replaced with 200 μL of adipocyte maintenance medium well^–1^ (DM without IBMX and DEX) containing the respective controls, samples, or rosiglitazone. The medium was renewed every other day during the 6 days maintenance period.

### 2.6 Fixation and Staining

After 11 days, cells were fixed with 2% paraformaldehyde and costained with NileRed and NucBlue. Imaging was carried out on the Cytation 5 cell imaging multimode reader (BioTek). Three images per field (Brightfield, NucBlue, and NileRed) and nine fields per well were captured. See the Supporting Information (Fixation and Staining) for details.

### 2.7 Image Analysis

**Figure 1:**
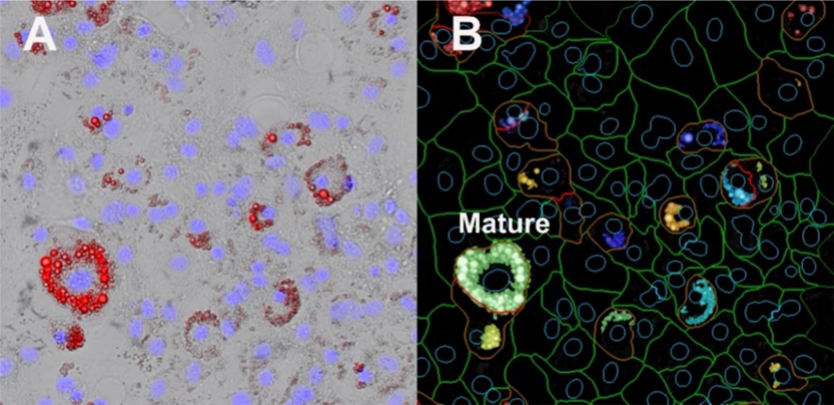
Image analysis example showing differentiated 3T3-L1 cells exposed to rosiglitazone (4.69 nM). (A) Merged brightfield and fluorescence images. Nuclei are stained with NucBlue (blue) and lipid with NileRed (red). (B) Corresponding object identification performed with CellProfiler. Nuclei are outlined in blue, cell boundaries in green, and adipocytes in red. Identified lipid droplets are shown as a solid color, and all lipid droplets associated with a given adipocyte are displayed in the same color. The images contain an example of a mature adipocyte (Mature).

Images were analyzed in the open-source software CellProfiler.^29^ A description of the image analysis protocol is in the Supporting Information (CellProfiler Analysis) and the pipelines are available at Zenodo (DOI 10.5281/zenodo.5513372). We quantified proliferation based on the total number of cells in an image (nuclei count), and adipogenesis was assessed by multiple endpoints: the total number of lipid droplets per image (lipid droplet count), the total area occupied by lipid droplets per image (total area), and the total intensity of the NileRed staining within the lipid droplets per image (total intensity). We also investigated the lipid content of individual adipocytes using single-cell analysis, where an adipocyte was defined as a cell containing at least one lipid droplet. We measured the total area occupied by lipid (lipid droplet area per adipocyte) and the average intensity of NileRed staining (average fluorescent intensity per adipocyte). These measurements were used to count the number of mature adipocytes [defined as having a lipid droplet area ≥ 8 average-sized lipid droplets (1000 pixels)] per image and to compare the lipid content of the adipocytes between treatment groups. To control for potential cross-plate differences in staining intensity, the average fluorescence intensity per adipocyte was normalized to the mean average fluorescence intensity for an internal plate control (adipocytes treated with 300 nM rosiglitazone). An example of the images that were captured and visualized output from the image analysis is shown in Figure 1.

### 2.8 Reporter Gene Assays

We performed the CALUX reporter gene assays, which are based on U2OS cell lines, in 384-well plates and used imaging of the NucBlue staining to count nuclei in a single 4× image to normalize the reporter gene response and to assess cytotoxicity. Rosiglitazone was the reference compound for PPAR*γ* and DEX for GR (Figure S4). See the Supporting Information (Reporter Gene Assays) for the detailed protocol.

### 2.9 Analysis of Bioassay Data

We used GraphPad Prism 9 (GraphPad Software, San Diego, CA) for nonlinear regressions and statistical analysis, and interpolated plastic equivalents inducing 10 or 20% effect (effect concentration, EC_10_, or EC_20_) from the respective dose–response curves. The limit of detection (LOD) of each endpoint and experiment was calculated as three times the standard deviation (SD) of pooled controls (i.e., a z-score of 3). Samples inducing an effect ≥LOD were considered adipogenic if they did so in at least two of the investigated endpoints. See the Supporting Information (Analysis of Bioassay Data) for details.

### 2.10 Nontarget Chemical Analysis

We analyzed all samples, except PLA 3, using ultrahigh performance LC-QTOF-MS/MS with an Acquity UPLC Waters liquid chromatography system coupled to a SYNAPT G2-S mass spectrometer (both Waters Norge, Oslo, Norway). The analytical method has been described in the study of Zimmermann et al.,^30,31^ and a brief description as well as information about the data analysis and compound identification can be found in the Supporting Information (Nontarget Chemical Analysis).

### 2.11 Comparison with Chemicals Known to Induce Adipogenesis

We built a list of 120 known adipogenic chemicals (Table S2) by searching the Web of Science (Core Collection) for studies investigating chemicals in the adipogenesis assay and complemented the search with chemicals reviewed by Amato et al.^20^ We cross-referenced the list with the tentatively identified compounds in the plastic samples based on our previous gas chromatography (GC)-QTOF-MS/MS analysis^19^ and the present LC-QTOF-MS/MS analysis to determine whether some of these compounds are MDCs (Table 1). See the Supporting Information (Comparison with Chemicals Known to Induce Adipogenesis) for details.

## 3 Results

### 3.1 Adipogenic Activity of Plastic Consumer Products

**Figure 2:**
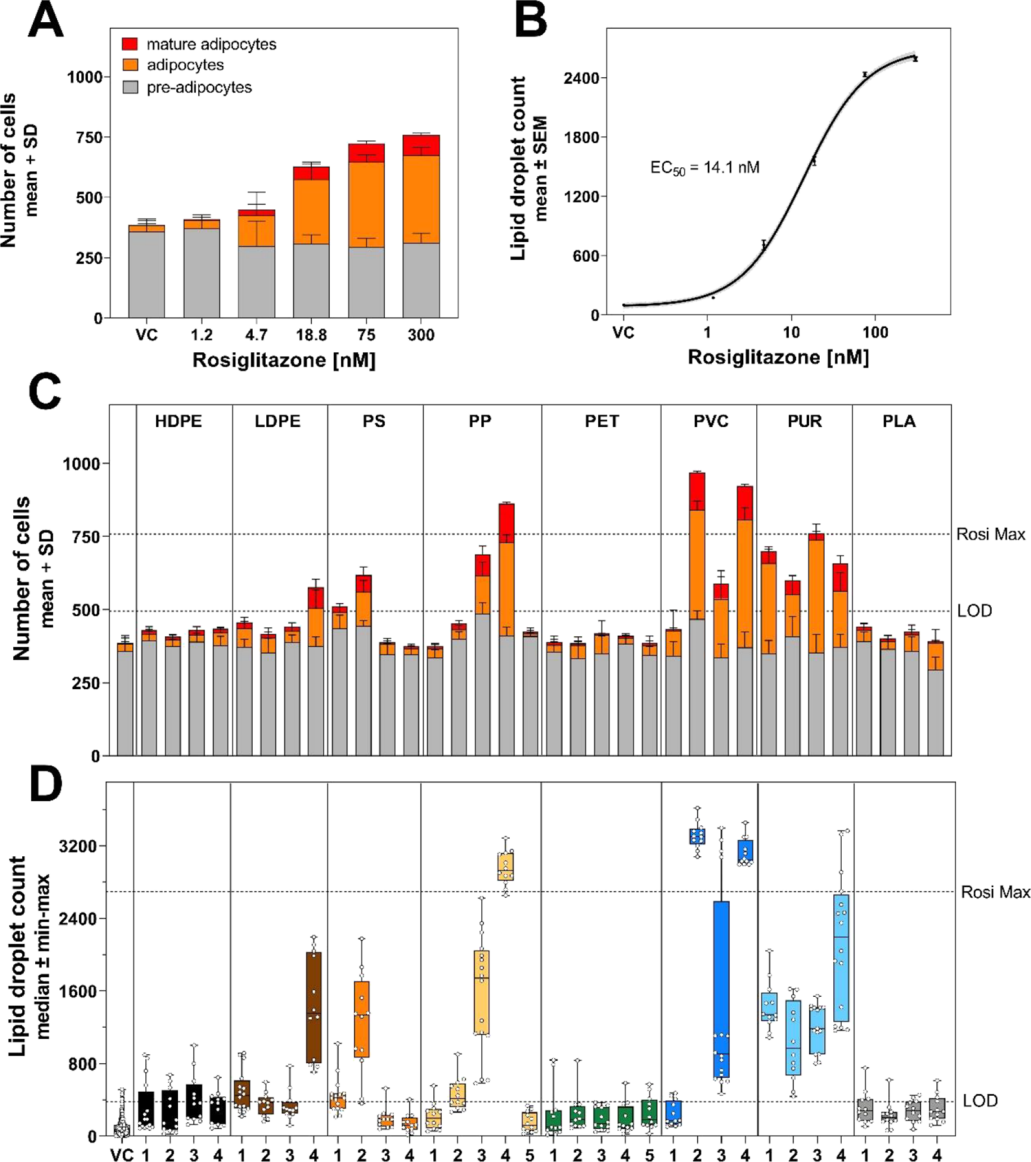
Effect of rosiglitazone on (A) the adipocyte population and (B) the lipid droplet count (pooled data from four experiments). Effect of plastic extracts on (C) the adipocyte population and (D) the lipid droplet count in the highest noncytotoxic concentration. The highest noncytotoxic concentration was 3 mg plastics well^–1^ except for PP 4 (1.5 mg plastic well^–1^) as well as PUR 2 and PUR 3 (0.75 mg plastic well^–1^). VC = vehicle control, LOD = limit of detection, Rosi Max = maximal response of rosiglitazone.

To exclude cytotoxic effects masking the adipogenic response, we used nuclei count data to assess cytotoxicity (>20% lower nuclei counts compared to vehicle controls). Most extracts were not cytotoxic up to the maximum concentration tested (3 mg plastic well^–1^), except for PP 4, PUR 3, and PUR 4. The latter two were the most cytotoxic samples with the highest noncytotoxic concentration (HNC) being 0.75 mg plastic well^–1^. The HNC for PP 4 was 1.5 mg plastic well^–1^ (Figure S5). To assess the induction of adipogenesis by the plastic extracts, we present the numbers of adipocytes and mature adipocytes in the cell populations and the total lipid droplet count per image for the HNC of each sample. Data for these endpoints were compared to both vehicle and rosiglitazone-treated controls (Figure 2). Dose–response relationships for all endpoints and example images can be found in the Supporting Information (Figures S6–S20).

The extracts of 11 plastic consumer products induced adipogenesis with four samples having an equal or stronger effect than the maximal response of cells exposed to rosiglitazone (PVC 2 and 4, PP 4, and PUR 3). Similar to rosiglitazone (Figure 2A), the proliferative effect of the plastic extracts was driven by an increase in the numbers of adipocytes and mature adipocytes, while the number of preadipocytes remained stable (Figure 2C). Regarding the polymer type, the extracts of PUR and PVC products were the most potent, with seven out of eight samples inducing adipogenesis, whereas for PP, PS, and LDPE, only specific samples induced adipogenic responses. In contrast, PET, HDPE, and PLA samples were consistently inactive. The same pattern is reflected in the lipid droplet count data (Figure 2D). Here, however, some additional samples induced a slight increase in lipid droplets (LDPE 1, PS 1, and PP 2).

**Figure 3:**
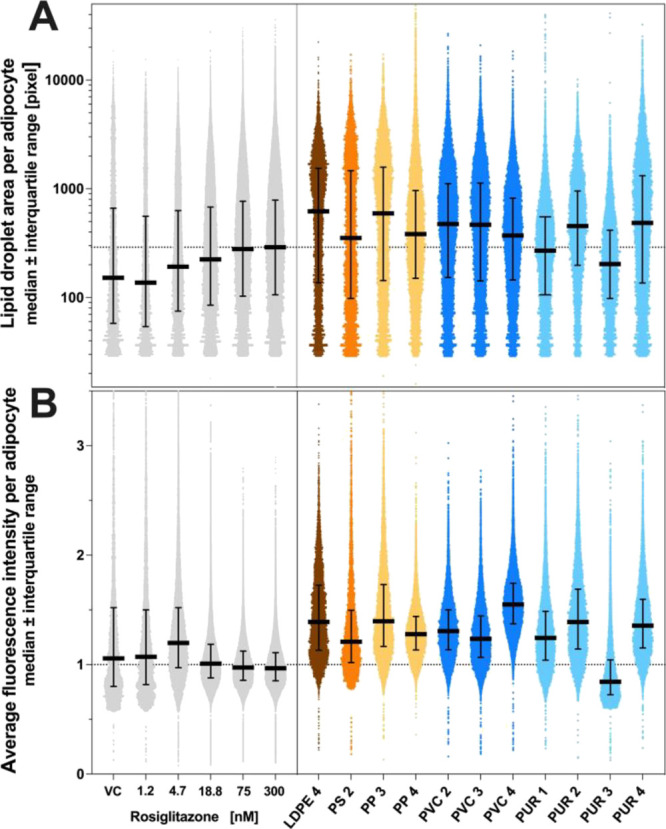
(A) Size distribution of the adipocyte population and (B) accumulation of triglyceride per adipocyte in cells exposed to rosiglitazone (left) or the highest noncytotoxic concentration of the 11 active plastic extracts (right). Single-cell data from one experiment. Intensity data are normalized on the mean of the highest rosiglitazone concentration (300 nM). VC = vehicle control.

Having demonstrated that certain plastic extracts were potent stimulators of adipogenesis, we next wanted to explore the lipid content of the resulting adipocytes. Given the propensity of environmental pollutants to promote unhealthy adipogenesis, we used the single-cell data to look specifically at whether the adipocytes generated by plastic extract exposures accumulated more lipid than those generated by exposure to rosiglitazone (Figure 3). We present here the results from one out of the four experiments (full results in Figure S21). Exposure to rosiglitazone dose-dependently increased the lipid content of adipocytes. A median lipid droplet area of 137 pixels cell^–1^ was measured at the lowest concentration versus 290 pixels cell^–1^ at the highest concentration (Figure 3A), while the average fluorescence intensity of the lipid droplets remained stable (Figure 3B). Thus, the adipocytes increased in size in response to rosiglitazone, but triglyceride accumulation within the droplets remained constant. Compared with the maximal response to rosiglitazone, adipocytes exposed to many of the active plastic extracts were larger and had a higher triglyceride content. The lipid droplet area per adipocyte was greater in 9 of the 11 active samples with a median increase of 21.6–114% (PS 2–LDPE 4), and the average fluorescence intensity was higher in 10 of the 11 active samples with a median increase of 25.1–60.4% (PS 2–PVC 4). These effects were consistent across all experiments, except for PVC 3 (Figure S21).

### 3.2 Reporter Gene Assays

We observed that plastic extracts were more cytotoxic in the U2OS cells used in the reporter gene assays than in the 3T3-L1 cells with five samples being cytotoxic. The most cytotoxic sample was PP 4 with an HNC of 0.19 mg plastic well^–1^, followed by PS 2, PP 3 (HNC of 0.38 mg plastic well^–1^), as well as PLA 1 and PVC 2 (0.75 mg plastic well^–1^, Table S4).

**Figure 4:**
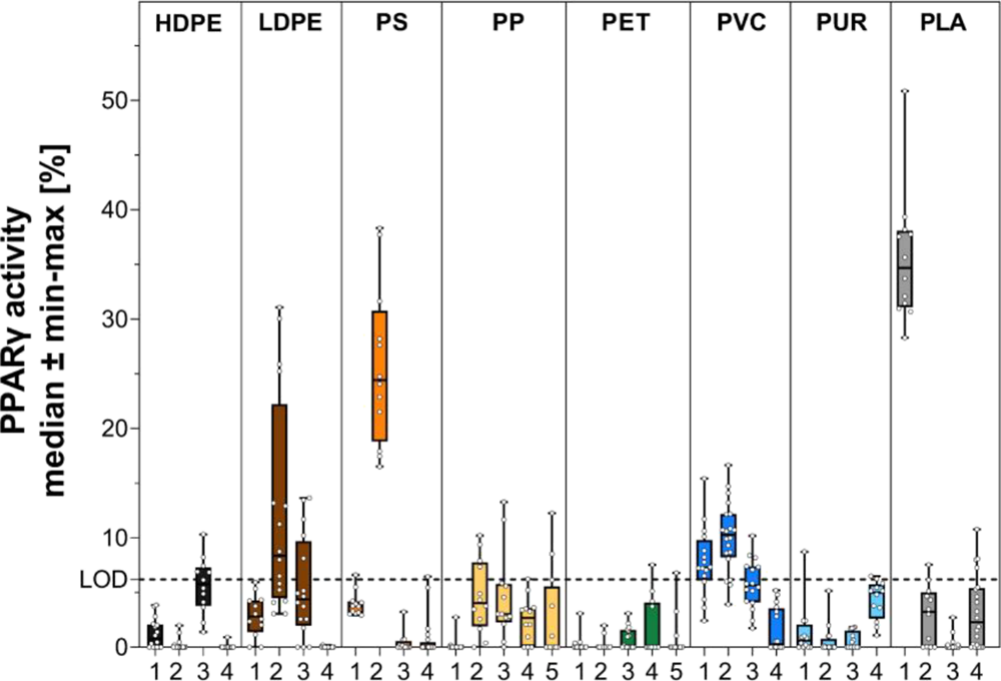
PPARγ activity induced by plastic extracts at the highest noncytotoxic concentration. The highest noncytotoxic concentration was 1.5 mg plastic well^–1^, except for PP 4 (0.19 mg plastic well^–1^), PS 2, and PP 3 (0.38 mg plastic well^–1^) as well as PLA 1 and PVC 2 (0.75 mg plastic well^–1^).

None of the samples activated GR (Figure S22). Five extracts activated PPARγ (Figure 4), and PLA 1 was the most potent sample with a median receptor activity of 34.7%, followed by PS 2 (24.4%), PVC 2 (10.3%), LDPE 2 (8.4%), and PVC 1 (7.3%). Accordingly, the PPARγ activity of the plastic extracts is a poor predictor of their adipogenic activity (Figure S23), except for PVC 2 and PS 2, which induced both PPARγ and adipogenesis at similar concentrations. Moreover, three out of the five samples activating PPARγ did not induce adipogenesis in 3T3-L1 cells (PLA 1, LDPE 2, and PVC 1).

### 3.3 Chemicals Tentatively Identified in Plastics

Using nontarget GC-QTOF-MS/MS, we previously identified 260 unique chemicals in extracts of the same plastic products.^19^ This corresponds to 231 tentatively identified chemicals with 227 unique PubChem CIDs in the samples used in this study (Table S3). In the nontarget LC-QTOF-MS/MS analysis performed here, we detected in total 55,300 features (e.g., unidentified chemicals) across all samples that had a >10-fold higher abundance compared to the blanks or were only present in samples. Here, the number of features in individual samples ranged from 107 (HDPE 2) to 6242 (PUR 3, Table 1). In total, 5500 features had MS/MS data that we could use for compound identification, out of which we detected between 30 (PS 4) and 2117 features (PUR 3) per sample.

For tentatively identifying the plastic chemicals, we used the MS/MS data with the MassBank library (14,788 compounds) and three in silico-fragmented databases of chemicals potentially used in plastics or (pre)registered for authorization on the European market (in total 75,510 compounds).^31^ These queries resulted in a successful identification of 2364 features across all samples, corresponding to 629 unique chemicals (Supporting Information Excel Table S1). Accordingly, 6 (PLA 2) to 33% (PET 1) of the features in each sample were tentatively identified. For the 25 compounds with the highest identification scores (≥50) and abundance in the samples, we confirmed the plausibility of the identification by checking whether the compounds are known to be used in plastics (Supporting Information Excel Table S2). We found that 14 out of 25 compounds are used in plastics, including five plasticizers (e.g., acetyl tributyl citrate), four flame retardants (e.g., tris(2-butoxyethyl) phosphate, tris(3-methylphenyl) phosphate), and multiple processing aids, such as the lubricant 2-nonyl-N-(2-nonylphenyl) aniline, the hardener 4-methylphthalic anhydride, and the slip additive (Z)-docos-13-enamide. We also identified compounds that probably migrated from the packed content into the packaging (two octadecanamides used in cosmetics) and one compound that was implausible (the veterinary drug febarbamate).

When cross-referencing the chemicals tentatively identified in plastics against a list of known MDCs (Table S2), we found 11 compounds known to induce adipogenesis in 3T3-L1 cells. The MDCs present in our samples included four phthalates and six organophosphates (Table 1). Benzyl butyl phthalate (BBP), dibutyl phthalate (DBP), and di(2-ethylhexyl) phthalate (DEHP) were present in PVC 4. Di-iso-nonyl phthalate (DINP) was detected in PVC 3 and 4. Diphenyl phosphate (DPP), 2-ethylhexyl diphenyl phosphate (EHDP), and triphenyl phosphate (TPP) were detected in multiple samples. When using raw abundance as a proxy for concentration, high levels of TPP, DPP, and EHDP (the MDCs present in at least three samples) were detected in two to three active PVC samples (Table S5). In contrast, the other active samples contained very low levels of these chemicals (PS 2, PP 4, PUR 2, and PUR 3). Interestingly, we did not detect organotin compounds or bisphenols (Supporting Information Excel Table S1) despite these being known MDCs and thought of as being common in PVC and other plastics.^9,20^ This can either be due to the limited sensitivity of our nontarget screening (e.g., data-dependent acquisition and positive ionization in the LC-QTOF-MS/MS, and lack of derivatization in the GC-QTOF-MS/MS) or the absence of those compounds in the products we selected.

## 4 Discussion

### 4.1 Adipogenic Activity of Plastic Consumer Products

To test our hypothesis that plastic products contain MDCs, we characterized the adipogenic activity of all compounds extractable from plastic consumer products. Eleven out of 34 products contained chemicals that induce adipogenesis and are, thus, MDCs in vitro (Figure 2). The chemicals extracted from some plastics trigger potent effects that are similar to or higher than those induced by the reference compound rosiglitazone (PVC 2 and 4, PP 4). Supramaximal efficacies have previously been reported for single compounds, such as dibutyl phthalate and tert-butyl phenyl diphenyl phosphate^32^ but only at concentrations ≥10 μM, illustrating the potency of the extracted mixtures.

Products with multiple applications, including two FCMs (PS 2, PP 3) and nine non-FCMs, contained adipogenic chemicals. While chemicals migrating from packaging into food represent an obvious source of human exposure,^33^ compounds released from non-FCMs can also contribute via dermal uptake (e.g., PUR 4 shower slippers) or inhalation. For instance, dust contains chemical mixtures that induce adipogenesis.^32^ Here, we show that plastic flooring (e.g., PVC 4) contains MDCs that may contribute to human exposure if they partition into dust. Given the potency of the extracted mixtures and considering our close and constant contact with plastics, our results support the idea that plastic chemicals can contribute to an obesogenic environment and, thus, the obesity pandemic.

The chemicals present in PVC and PUR products most consistently induced potent adipogenic responses, while compounds extracted from PET, HDPE, and PLA products were inactive. Apart from the PLA samples, this is in line with our previous findings for other toxicity endpoints.^19^ This suggests that PVC and PUR are more likely to contain MDCs compared to other polymers. However, the chemicals extracted from some PP, PS, and LDPE products also induced adipogenesis (Figure 2). This further corroborates the notion that caution is needed when trying to generalize the occurrence of toxic chemicals based on the polymer type.^19^

Unhealthy or dysfunctional adipocytes are part of the obesity phenotype. They are larger, have an impaired glucose uptake and insulin signaling, an elevated inflammatory response, and decreased respiration.^34^ While we did not investigate the latter characteristics, adipocytes exposed to plastic chemicals were larger and contained more triglycerides compared to those treated with rosiglitazone (Figure 3). Because rosiglitazone promotes the development of healthy white adipocytes,^35,36^ these results suggest that exposure to plastic chemicals could shift adipocytes toward an unhealthy phenotype. Similar trends have been reported for a range of MDCs, including BPA,^37^ organotin compounds,^38,39^ and DEHP,^40^ which we detected in PVC 4 (Table 1). Hence, it will be interesting to investigate whether plastic chemicals also trigger the other hallmarks of unhealthy, dysfunctional adipocytes.

### 4.2 Plastic Chemicals and Adipogenesis

Using nontarget high-resolution mass spectrometry, we show that plastic products contain hundreds to thousands of extractable chemicals, of which only a minority was identifiable using spectral libraries and in silico tools. This is in line with our previous research^19,31^ and points toward the presence of unknown chemicals in plastics (e.g., nonintentionally added substances). Accordingly, the relatively low identification performance in our study is a result of the limited coverage of chemical databases. These limitations notwithstanding, we tentatively identified a range of known plastic chemicals providing confidence in the accuracy of the identifications.

The plastic products contained known MDCs, including four phthalates (only in PVC 3 and 4) and six organophosphates (Table 1). Biomonitoring data suggest that humans are commonly exposed to some of these compounds.^41−43^ As an example, the phthalates DBP and DEHP, as well as the flame retardants TPP and TBEP we found in plastics, were recently detected in matched maternal and cord blood samples.^44^ Accordingly, plastic products can be one source of exposure to these MDCs.

Known MDCs may explain the adipogenic response to chemicals extracted from some but not all plastic samples. Most active samples contained at least one MDC with TPP, DPP, and EHDP being present in multiple samples. Interestingly, we detected 10 known MDCs in the floor covering (PVC 4). While the active PVC samples had high levels of TPP, DPP, and EHDP, the abundance of these chemicals was very low in the other active samples (Table S5). This suggests that compounds other than the known MDCs contributed to the adipogenesis induced by the plastic extracts.

### 4.3 Underlying Mechanisms

PPARγ is a key regulator of adipogenesis,^23^ and many MDCs that induce adipogenesis also activate PPARγ.^9^ Despite the common idea that PPARγ activation is a main mechanism via which anthropogenic chemicals trigger adipogenesis, most of the adipogenic plastic samples in fact did not activate this receptor (Figure 4). Only in two cases (PVC 2, PS 2) did a high PPARγ activity correspond to a strong induction of lipid droplet formation. Moreover, three samples (PLA 1, LDPE 2, and PVC 1) activated PPARγ but were inactive in the adipogenesis assay. Thus, the adipogenic effects of the plastic extracts are not necessarily dependent on the direct activation of PPARγ, and other mechanisms must be involved.

GR is another important nuclear receptor that participates in adipogenesis, and various MDCs activate GR.^45^ In particular, glucocorticoids are essential in inducing adipocyte differentiation (Figure S3). However, none of the plastic extracts activated GR, rendering this an unlikely mechanism of action in this case.

Elucidating the mechanism by which plastic chemicals induce adipogenesis is complex because we deal with two black boxes, namely, the complex chemical mixtures present in plastics and the multitude of potential mechanisms of action involved in adipogenesis in 3T3-L1 cells.^22^ In addition to PPARγ and GR, (ant)agonists of multiple other nuclear receptors, such as the retinoid X receptor α, estrogen receptor, androgen receptor, liver X receptor, and thyroid receptor β, have been demonstrated or are discussed to contribute to adipogenesis.^46^ In light of the diversity of compounds we detected in plastics, it appears probable that these act via multiple mechanisms that are in most cases PPARγ- and GR-independent. Although more work needs to be done to elucidate the underlying mechanisms, our results underline the importance of using integrative methods, such as the adipogenesis assay, to identify MDCs triggering cellular responses rather than assessing (anta)agonism at selected nuclear receptors.

### 4.4 Limitations and Future Directions

To the best of our knowledge, this is the first study investigating the adipogenic activity of chemicals extractable from plastic consumer products. Considering the diversity of plastic products and their chemical composition, the sample set is certainly not representative of all plastic chemicals humans are exposed to. While it is challenging to comprehensively characterize the human exposure to plastic chemicals from all types of products, given their ubiquity and diversity, a way forward is to prioritize polymer types that are likely to contain MDCs, such as PVC and PUR.

Given that we aimed at investigating whether MDCs are present in plastic products, we used methanol to extract the samples. This simulates a worst-case scenario. Thus, even though we demonstrated that potent (mixtures of) MDCs are present in consumer products, it remains to be investigated whether these will migrate under more realistic conditions into air, water, or food, or can be taken up dermally. Using the same samples as in the present study, we recently demonstrated that a significant number of chemicals that cause in vitro toxicity, such as antiandrogenic compounds, migrate into water.^31^ However, it remains unknown if this is also the case for the MDCs described here.

Moreover, because we aimed at investigating final products, we analyzed plastic packaging that contained foodstuff or personal care products. Because chemical migration is not a one-way street, we cannot exclude the possibility that compounds from the contents migrated into the packaging. The detection of chemicals used in cosmetics in its packaging underlines this limitation. Such compounds may contribute to the observed adipogenic activity or PPARγ activation, and future research should cover unused final packaging.

The nontarget chemical analysis resulted in the tentative identification of several MDCs. However, many compounds remain unidentified, and there is some likelihood of false-positive identifications. The challenge of a rather low identification success is well known for environmental pollutants^47^ and can be addressed by building more comprehensive spectral databases. Recent efforts to build specific databases for plastic chemicals are promising,^17,18^ but must be complemented with spectral information and nonintentionally added substances. In addition, we show that known MDCs only partially, if at all, contribute to the adipogenesis induced by plastic extracts. This points toward the presence of unidentified MDCs in plastics. To identify the compounds that are indeed causative for the observed responses, future research should apply effect-directed analysis.

Moreover, while our results indicate that plastic chemicals may promote development toward unhealthy adipocytes, more evidence is needed to further support this hypothesis. For instance, one needs to extend the adipogenesis assay to cover later stages of adipocyte development and investigate biomarkers of inflammation and metabolic function (e.g., glucose uptake and insulin sensitivity).

Taken together, we demonstrated that plastic consumer products contain potent (mixtures of) MDCs that induce adipogenesis in vitro via mechanisms that are, for the most part, not mediated via PPARγ or GR. Accordingly, and considering our constant contact with a multitude of plastic products, we conclude that plastic chemicals may contribute to an obesogenic environment. Given that the plastic products containing MDCs also contained compounds triggering other toxicological endpoints,^19^ a shift toward chemically less-complex plastics represents a way forward to a nontoxic environment.

## Supporting Information Available

List of the used chemicals and consumables, results of the optimization experiments, dose–response relationships for the reference compounds and samples, example images of active samples, list of chemicals inducing adipogenesis, cytotoxicity results, single-cell data from four independent experiments, tentatively identified compounds, comparison of the adipogenic effects of plastic extracts and the abundance of frequently detected MDCs, as well as bioassay data (PDF)
Tables of tentatively identified chemicals and bioassay data (XLSX)

## Notes

The manuscript has been published as a preprint on bioRxiv.^48^ The raw mass spectral data can be accessed under DOI 10.18710/6MEOMA and the CellProfiler pipelines under DOI 10.5281/zenodo.5513372.
