# Supplementary material for: Adipogenic Activity of Chemicals Used in Plastic Consumer Products
Source: Environ Sci Technol. 2022 Jan 26;56(4):2487–96. doi: 10.1021/acs.est.1c06316 (PMC8851687; doi:10.1021/acs.est.1c06316)
Supplement: Supplementary file 1 — es1c06316_si_001.pdf [file es1c06316_si_001.pdf]

Supporting Information for

Adipogenic activity of chemicals used in plastic consumer products

Johannes Völker[1], Felicity Ashcroft[1], Åsa Vedøy[1], Lisa Zimmermann[2] and Martin Wagner[1]*

[1] Department of Biology, Norwegian University of Science and Technology (NTNU), 7491 Trondheim, Norway

[2] Department of Aquatic Ecotoxicology, Goethe University Frankfurt am Main, 60438 Frankfurt am Main, Germany

*corresponding author: Martin Wagner, Email: martin.wagner@ntnu.no

Number of pages: 53

Number of figures: 24

Number of tables: 5

Supporting Materials and Methods

Plastic extraction

We used the original samples stored in glassware, except for PLA 3. As there was not enough material available for the original PLA 3 sample and we were not able to obtain more of the same product, we replaced the sample with a PLA coffee lid sample. To avoid sample contamination, glass or polytetrafluorethylene consumables were used for the sample extraction and all material was rinsed twice with acetone and annealed at 200 °C for ≥ 3 h. The samples were cut into 0.5–0.8 × 2 cm pieces. Foamy products were cut to a thickness of 0.5 cm. We weighed three grams each into 1 or 2 transparent glass vials depending on the sample volume, added 20 mL methanol (≥ 99.9%, Sigma Aldrich), and extracted the samples by sonication in an ultrasonic bath for 1 h at room temperature. We then transferred the methanol into clean glass vials, added 200 µL dimethyl sulfoxide (≥ 99.5%, Sigma Aldrich) as a keeper and evaporated the samples under a gentle stream of nitrogen to a final volume of 200 µL. Further, we treated three procedural blanks (PB 1–3) not containing any sample identically to control for contamination and stored the final extracts at -20 °C prior to analysis.

Cell culture conditions

3T3-L1 cells were cultured in preadipocyte medium (PAM: DMEM-high supplemented with 10% bovine calf serum and 1% penicillin/streptomycin). Culturing 3T3-L1 cells over multiple passages can cause a decline in differentiation efficiency due to the loss of contact inhibition.[1] Thus, cryo-cultures of passage 9 were thawed, subcultured once upon reaching 60-80% confluency, and sub-confluent cells of passage 10 were used for all experiments to ensure comparability and preserve differentiation capability. CALUX cells were maintained in growth medium (DMEM/F-12 supplemented with 7.5% fetal bovine serum, 1%

penicillin/streptomycin, and 0.2 mg mL^{-1} G418 and non-essential amino acids) and subcultured twice weekly and used until passage 20.

Optimization of the adipogenesis assay

We conducted optimization experiments to identify a suitable concentration of dexamethasone (DEX) to initiate adipocyte differentiation. Moreover, we applied high-content fluorescence imaging combined with an automated image processing in addition to the fluorescence readout $well^{-1}$ at the end of the experiment and compared both methods with regards to sensitivity.

Following the growth arrest window, the medium was replaced with 200 µL differentiation medium $well^{-1}$ (DM: DMEM-high supplemented with 10% FBS, 1% penicillin/streptomycin, 20 mM HEPES, 1 µg mL^{-1} insulin and 0.5 mM 3-isobutyl-1-methylxanthine (IBMX)) containing either none or six concentrations of dexamethasone (6.25–250 nM) and eight concentrations of the reference compound rosiglitazone (300 pM – 1 µM). After the 48-h differentiation window, we replaced the medium with 200 µL adipocyte maintenance medium $well^{-1}$ (DM without IBMX and DEX) containing the eight rosiglitazone concentrations and changed the medium every other day during the maintenance period.

Counting nuclei based on NucBlue staining using the imaging approach was more sensitive than the standard fluorescence readout for detecting the proliferative effect of rosiglitazone (Figure S3 A) with an EC_{50} of 16.3 and 41.2 nM for nuclei counts and fluorescence readout, respectively. In contrast, quantification of adipogenesis had comparable sensitivity (Figure S3 B) with an EC_{50} of 10.6 and 11.4 nM for lipid droplet intensity and total NileRed fluorescence readout, respectively. However, the dynamic range of the assay was greatly enhanced using the imaging approach with an 11.7-fold increase in the lipid droplet count at the highest rosiglitazone concentration versus 4.69-fold increase in the fluorescence readout. In addition, the imaging-based approach provides more information including the

characterization of the differentiation stage of cells in the population, and single-cell measurements to quantify the size of adipocytes and triglyceride accumulation (e.g., number and size of lipid droplets). Thus, high-content imaging with automated image processing can greatly extend our capabilities fir screening of MDCs *in vitro*.

Glucocorticoids in the differentiation medium are essential to prime the preadipocytes for adipogenesis and the differentiation success is variable and weak when DEX is absent (Figure S24). In contrast, an excess of DEX (>25 nM) results in a significant stimulation of adipogenesis without an additional inducer and, thus, reduces the capability of the assay to detect adipogenic responses (Figure S3 C). This is in line with a previous study reporting up to 40% of adipocytes in the vehicle controls using 250 nM DEX in the differentiation medium.[2] Accordingly, the use of DEX concentrations varying from 0[3-5] up to 1 µM[1, 2, 6, 7] might contribute to the poor reproducibility and comparability of 3T3-L1 studies.[8] Based on our experiments, we recommend using a rather low DEX concentration of 6.25 nM which was sufficient to initiate adipocyte differentiation without increasing the assay baseline (Figure S3 D).

Fixation and staining

After 11 d, the medium was removed, and cells were rinsed with PBS and fixed with 2% paraformaldehyde for 10 min on ice. The fixative was removed, and cells were rinsed twice with PBS and stored at 4 °C prior to staining. Cells were co-stained with 100 µL NileRed solution well^{-1} (19.5 mL PBS + 500 µL AdipoRed (N3013, Lonza) and 1 drop mL^{-1} NucBlue (R37605, Thermo, Hoechst 3342 staining)). Plates were incubated for 40 min in the dark at room temperature. Stained cells were washed twice with PBS and stored at 4 °C prior to analysis.

Fluorescence per well was measured using a Cytation 5 Cell Imaging Multimode reader (BioTek with excitation at 485 nm and emission at 572 nm for NileRed, and excitation at 360 nm and emission at 460 nm for NucBlue). Imaging was carried out on the same instrument using a 10× Plan Fluorite objective (WD10, NA 0.3). Image-based autofocusing of NucBlue fluorescence was used to select the image plane, and three images per field were captured (Brightfield, NucBlue and NileRed). A 365 LED with DAPI filter cube (Ex 377/50, Em 447/60) was used to detect the NucBlue staining, and a 523 LED with RFP filter cube (Ex 531/40, Em 593/40) for NileRed. Nine fields were captured per well.

Cell profiler analysis

For the adipogenesis assay, NucBlue and NileRed staining imaged at x10 magnification were analyzed using the following protocol to generate the assay measurements described.

1. Cell identification: nuclei (primary objects) were identified using an Otsu thresholding method based on NucBlue staining and used as the seed objects to identify cells (secondary objects). NileRed images were smoothed by gaussian filtration and used to guide the propagation algorithm for secondary object identification with a minimal threshold factor to limit the foreground.

2. Lipid droplet identification (adapted from Adomshick *et al.*)[9]: lipid droplets were identified using a minimum cross entropy thresholding method applied to the NileRed images followed by a filtration step based on the mean intensity per droplet.

3. Image-based measurements: the number of cells and the number of lipid droplets were counted in each image. We additionally measured the total area occupied by lipid droplets, and the intensity of the Nile Red staining in this region.

4. Single cell analysis: to measure the lipid content per cell, lipid droplets were assigned to a given parent and merged such that the total area occupied by lipid, and the average intensity of the NileRed staining in this region could be calculated.

5. *Data processing:* filtration steps were subsequently applied to identify adipocytes (any cell containing at least one lipid droplet), and mature adipocytes (a cell having a lipid droplet area ≥ 1000 pixels, equivalent to ≥ 8 average size lipid droplets.)

For the nuclear counts (reporter gene assays), NucBlue staining imaged at x4 magnification was analyzed using the following protocol to quantify the number of nuclei in a given field. Nuclear counts were used for normalization and calculation of cytotoxicity.

1. Image correction: we applied the background method, with smoothing based on a gaussian filter to calculate an illumination function which was applied to NucBlue images to correct for the uneven illumination resulting from imaging the 384 well plates at ×4 magnification.

2. Identification of nuclei: nuclei were identified using an Otsu thresholding method based on the corrected images and we filtered the resulting objects based on their shape (form factor) to obtain final nuclear counts.

The cell profiler pipelines can be accessed under DOI 10.5281/zenodo.5513372

Reporter gene assays

We performed the CALUX reporter gene assays (U2OS cell lines) in white clear polystyrene CellStar 384-well plates (781098, Greiner Bio-One). Trypsinized cells were resuspended in assay medium (DMEM/F-12 without phenol red supplemented with 5% charcoal-stripped FBS, 1% penicillin/streptomycin, non-essential amino acids). 3000 cells well^{-1} were seeded in 25 µL, and plates were incubated at 37 °C and 5% CO_2. Samples and reference compounds

were prepared in assay medium (2-fold higher than the final assay concentration) in six concentrations per sample serially diluted 1:2 or eight concentrations of the reference compound (rosiglitazone for PPARγ and dexamethasone for GR, Figure S4). After 24 h of incubation, 25 µL sample was added to the 25 µL assay medium well^{-1} (1-fold), resulting in final sample concentrations of 0.05–1.5 mg plastic well^{-1} (equivalent to 0.09–30 mg plastic mL^{-1}). After 23 h of exposure, the medium was replaced with 25 µL NucBlue staining solution well^{-1} (1 drop NucBlue per mL PBS, Thermo Fisher Scientific) and incubated for 30 min in the dark at room temperature.

Imaging was performed on the Cytation 5 Cell Imaging Multimode reader (BioTek) with a 4× Plan Fluorite objective (WD 17 NA 0.13) using a 365 LED with DAPI filter cube (Ex 377/50, Em 447/60) to detect NucBlue staining. A single field was captured per well. Following the imaging, a white sticker was placed on the transparent bottom of the plates, and the staining solution was replaced with 20 µL cell lysis buffer (25 mM pH 7.8 TRIS, 2 mM DDT, 2 mM CDTA, 10% glycerol, and 1% Tritron-X100), and cells were lysed by linear shaking for 3 min. Luminescence was measured (Cytation 5) for one second after injection of 30 µL illuminate mix (20 mM Tricine, 1.07 mM $C_4H_2Mg_5O_{14}$, 2.67 mM $MgSO_4 \times 7H_20$, 0.1 mM EDTA, 1.5 mM DDT, 539 µM D-Luciferine, 5.49 mM ATP) followed by quenching of the reaction with 30 µL 0.1 M NaOH.

Analysis of bioassay data

We used GraphPad Prism 9 (GraphPad Software, San Diego, CA) for non-linear regressions and statistical analysis. To express cytotoxicity, we normalized the nuclei count to the vehicle controls (0% cytotoxicity) and a value of zero (100% cytotoxicity). We used 20% as cytotoxicity threshold. When the value of an individual image was >20%, all channels of that image were excluded from further analysis (cytotoxic or out of focus). If the mean value of a

replicate exceeded 20%, the replicate was excluded. When more than one replicate per concentration exceeded the threshold, the concentration was defined as cytotoxic. Fluorescence and luminescence readouts were corrected for background (wells without cells). Percentage increase (proliferative effects) or fold induction over the corresponding vehicle control were calculated for each endpoint of the adipogenesis assay to compare both methods in the optimization experiments. To express agonistic activity in the reporter gene assays, luminescence data were normalized to the maximal assay response (100% activity: upper plateau of the dose-response relationship) of the corresponding reference compound and the mean value of the vehicle control (0% activity). The limit of detection (LOD) of each endpoint and experiment was calculated as three times the standard deviation (SD) of pooled controls (equivalent to a z-score of 3). For samples to be considered adipogenic, these had to trigger effects ≥LOD in at least two endpoints in the adipogenesis assays. For samples to be considered agonists of PPARγ or GR, the reporter gene activity had to be ≥LOD. Dose-response relationships for all investigated endpoints were calculated using a four-parameter logistic function constrained to the lower plateau (0% activity). The respective plastic equivalents inducing 10 or 20% effect (effect concentration, EC_{10}, or EC_{20}) were interpolated from the dose-response curves.

Nontarget chemical analysis

We analyzed all samples, except PLA 3, using ultra-high performance liquid chromatography coupled to a quadrupole time of flight spectrometer (LC-QTOF-MS/MS) with an Acquity UPLC Waters liquid chromatography system coupled to a SYNAPT G2-S mass spectrometer (both Waters Norge, Oslo, Norway). The analytical method has been described previously.[10, 11] In brief, we injected 2 µL sample, equivalent to the chemicals extracted from 1.5 mg plastic, and performed the chromatographic separation on an Acquity UPLC BEH C18 column equipped with a C18 guard column (both from Waters). The mass spectrometer equipped with

an electron-spray ionization source was operated in positive ionization mode with a mass range of 50–1200 Da at a resolution of 20,000. MS data were recorded from 2–35.5 min with a data-dependent acquisition (triggered when the threshold of an individual ion intensity exceeded 25,000 counts, maximum 15 precursor ions per survey scan) using a collision energy ramp (8–35 eV in the low mass region and 30–70 eV in the high mass region). After every 7th–8th sample, we analyzed a solvent blank (mobile phase, methanol or DMSO, n = 15) and a quality control sample (containing an aliquot of each sample). Two PBs from the extraction were also analyzed. The raw mass spectral data can be accessed under DOI 10.5281/zenodo.4781257 (published after publication).

Chemical data analysis and compound identification

We imported the data for the 15 blanks, two PBs, six quality controls, and 33 samples to Progenesis QI (version 3.0, Nonlinear Dynamics) and corrected for the lock mass of leucine enkephalin. We automatically aligned the retention times of all blanks and samples using the quality controls. Peak picking was performed on the samples using common adducts (M+H, M+2H, M+H-H2O, M+H-2H_2O, 2M+H, M+Na, M+2Na, M+H+Na, M+2H+Na, M+2Na+H, M+2Na-H), an automatic sensitivity, a minimum peak width of 0.02 min and a fragment sensitivity of 0.2% of the base peak.

We generated a list of chemical features which had MS/MS data and performed the further data analysis as described before.[11] Basically, we filtered for features that were not detected in the solvent and PBs or present in the samples with an at least 10-fold higher raw abundance than the maximum abundance of that feature in any of the blanks.

To tentatively identify the remaining features, we compared their mass spectra with the empirical spectra in MassBank (14,788 compounds, release version 2021.03, https://github.com/MassBank/MassBank-data/releases/tag/2021.03) and with three databases

covering chemicals present in plastic packaging (2680 compounds), registered under the REACH regulation in 2020 (7092 compounds) and (pre)registered under REACH in 2017 (65,738 compounds) using the Metascope algorithm in Progenesis QI with a precursor tolerance of 5 ppm and a fragment tolerance of 10 ppm. The compounds in the latter three databases were *in silico* fragmented using the Metascope algorithm. The resulting identification was accepted if the match score was > 40. When a feature had multiple identifications with a score > 40, the first hit with the highest score was accepted. The identification corresponds to confidence level 3 according to Schymanski *et al.*[12]

Comparison with chemicals known to induce adipogenesis

We built a list of know adipogenic chemicals by searching Web of Science (Core Collection) for studies investigating chemicals in the adipogenesis assay using the following search strings: "(3T3L1 OR 3T3-L1) AND toxic* AND chemical*" (58 hits) as well as "(3T3L1 OR 3T3-L1) AND (obesogen* OR metabolic disruptor*) AND in vitro" (241 hits). The search was conducted on March 22, 2021. We removed duplicates and reviews and screened the remaining 254 full text articles for studies that investigated the adipogenic activity of chemicals in 3T3-L1 adipocytes. We decided not to perform a quality assessment to keep the list broad and included from 47 suitable studies all chemicals which were reported to be adipogenic. We further complemented the list with the chemicals reviewed by Amato *et al.*[13] and ended up with a list of 120 adipogenic chemicals (Table S2). For comparison with our results, we added the associated PubChem CIDs.

To cross-reference this list with the compounds we tentatively identified in plastics, we built a joint compound list based on our previous GC-QTOF-MS/MS analysis[14] and the present LC-QTOF-MS/MS analysis. For the former, we translated the available CAS numbers of all tentatively identified compounds to SMILES using the US EPA's CompTox Dashboard

(https://comptox.epa.gov/dashboard) and then translated the SMILES to PubChem CIDs using the PubChem Identifier Exchange Service (https://pubchem.ncbi.nlm.nih.gov/idexchange). If CAS numbers were invalid or unavailable, we searched the compound name in PubChem and manually annotated the CID. For the LC-QTOF-MS/MS data, we used the PubChem CIDs provided by Progenesis QI or manually annotated the compound names provided by MassBank. The combined list from the GC- and LC-QFOT/MS/MS data contained 803 unique chemicals with CIDs (Table S3 and Excel Table S1). To determine whether some of these compounds are MDCs, we cross-referenced both CID lists (Table 1).

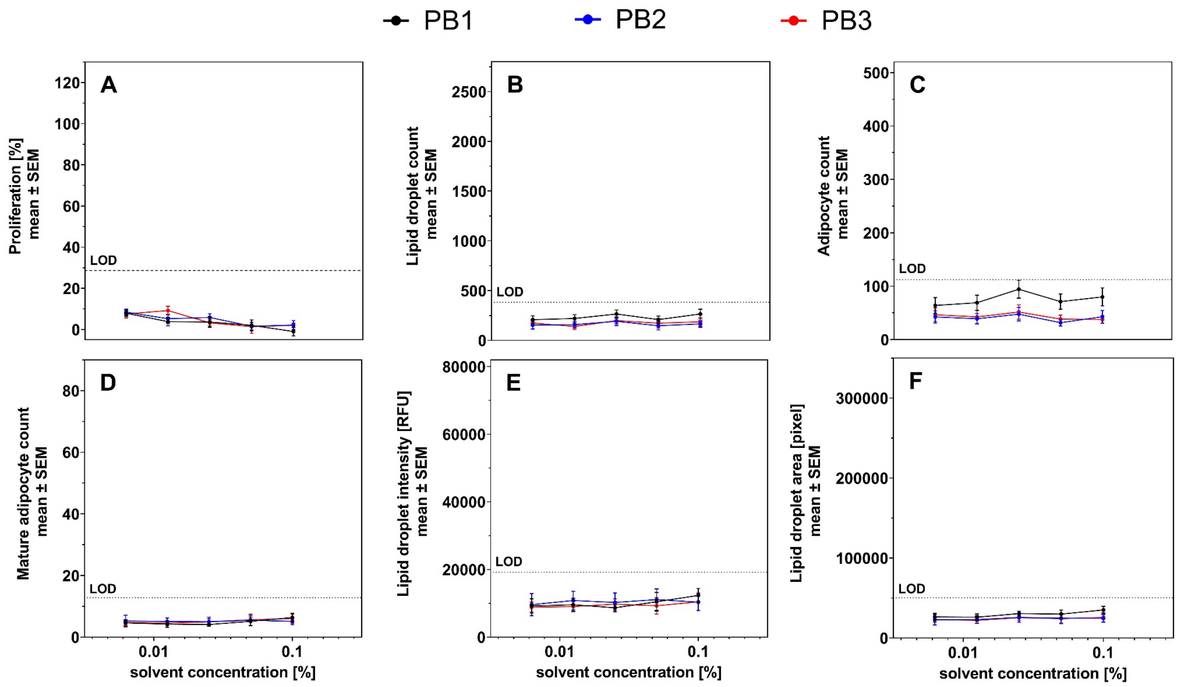

Figure S1. Dose-response relationships for the adipogenesis assay endpoints of the three procedure blanks (PB 1–3). (A) proliferation normalized on the mean of the vehicle control, (B) lipid droplet count per field, (C) adipocyte count per field, (D) mature adipocyte count per field, (E) total intensity of the NileRed staining within the lipid droplet mask per field and (F) total area occupied by lipid droplets per field. Twelve replicates per concentration (n = 12). LOD = limit of detection, RFU = relative fluorescence unit.

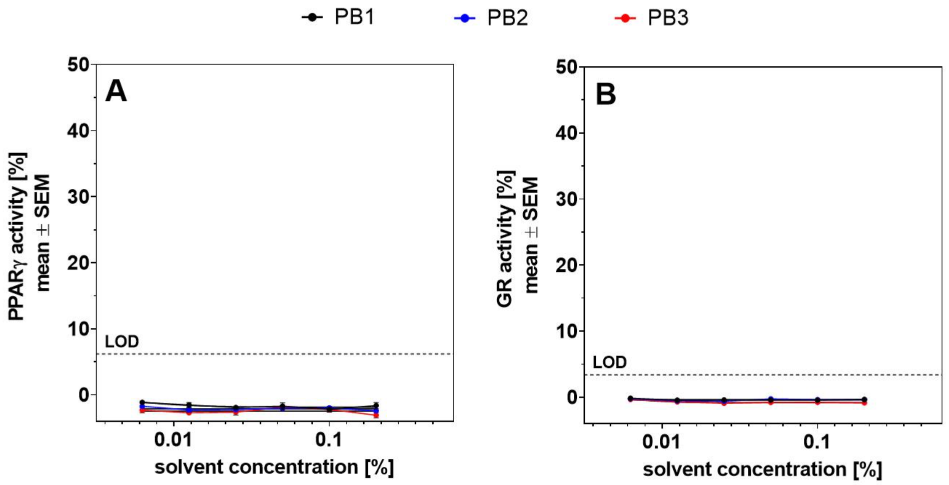

Figure S2. Dose-response relationships for (A) PPARγ and (B) GR activity of the three procedure blanks (PB 1–3). Twelve or more replicates per concentration (n ≥ 12). PPARγ = peroxisome proliferator receptor gamma, GR = glucocorticoid receptor, LOD = limit of detection.

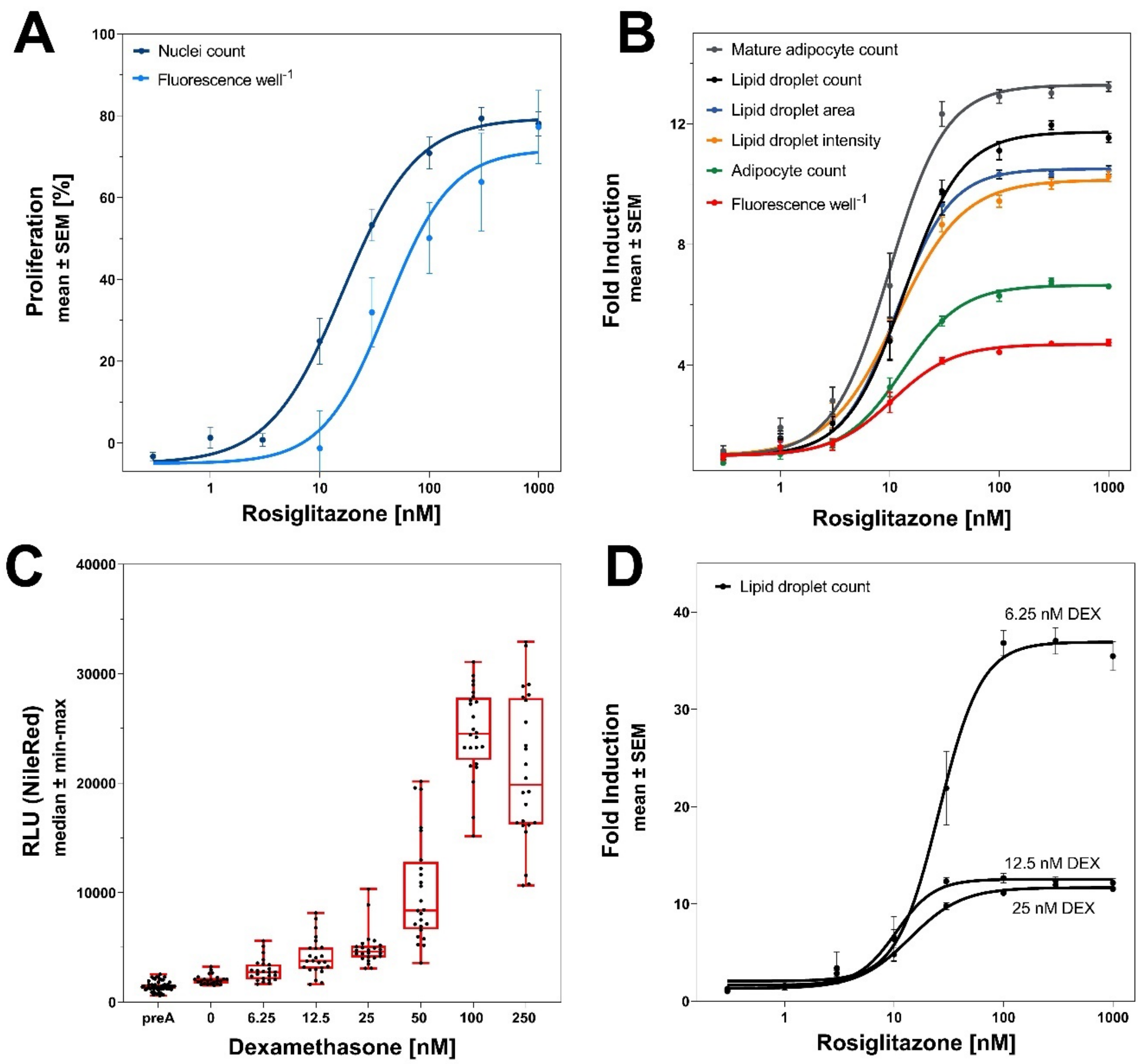

Figure S3. Optimization of the adipogenesis assay. (A) Proliferative effects induced by rosiglitazone analyzed by imaging (DAPI filter) and total fluorescence readout (NucBlue) with 25 nM dexamethasone (DEX) in the differentiation medium (DM). (B) Multiple adipogenic endpoints analyzed by imaging (RFP filter) compared to total fluorescence readout (NileRed) with 25 nM DEX in DM. (C) Total fluorescence readout (NileRed) for the preadipocyte control (preA, undifferentiated) and the differentiated vehicle controls with increasing dexamethasone concentrations in DM. (D) Lipid droplet count induction by rosiglitazone with 6.25, 12.5, 25 nM DEX in DM.

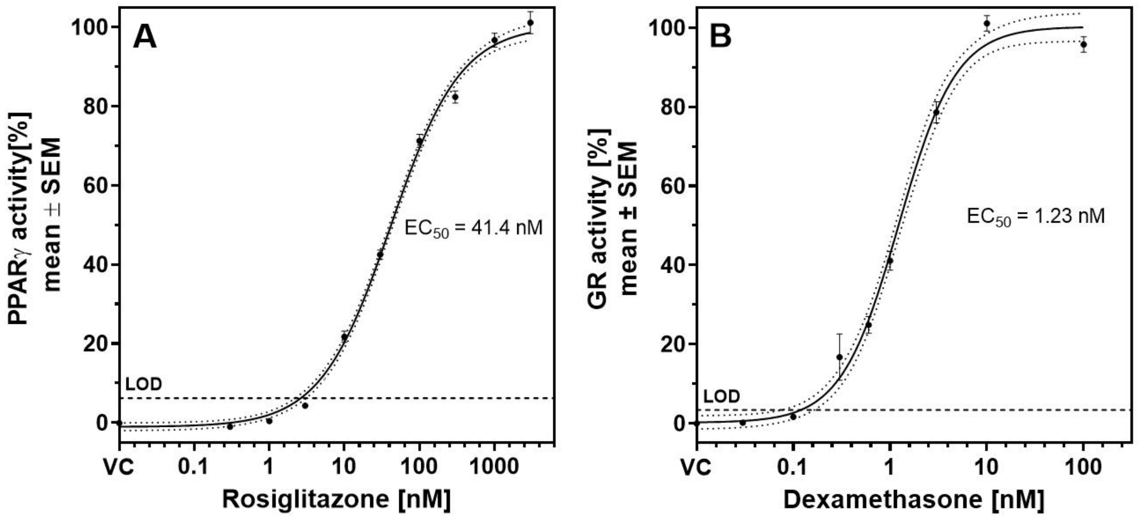

Figure S4. Dose-response relationships for (A) PPARγ and (B) GR activity of the reference compound rosiglitazone and dexamethasone. 48 or more replicates per concentration ($n \geq 48$). PPARγ = peroxisome proliferator receptor gamma, GR = glucocorticoid receptor, LOD = limit of detection, VC = vehicle control.

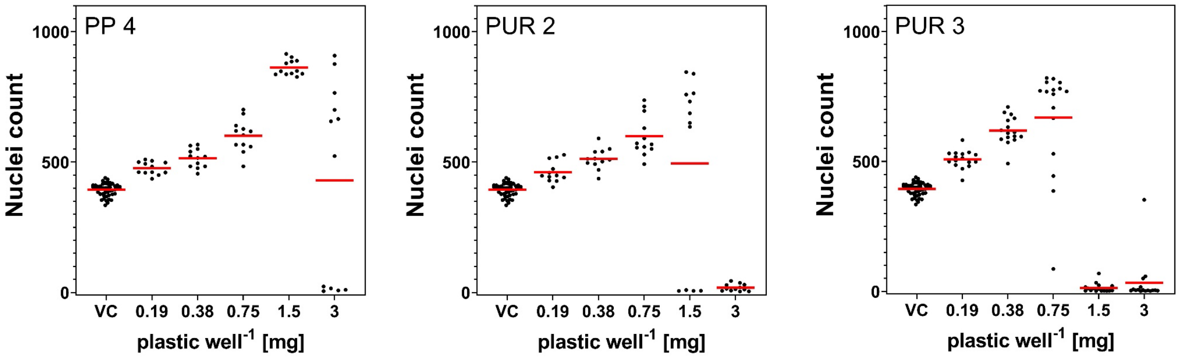

Figure S5. Nuclei count of the cytotoxic plastic extracts (PP 4, PUR 2, PUR 3) in the adipogenesis assay. Data is presented as mean count per field (red lines) from three to four independent experiments performed with four replicates each (dots, $n \geq 12$). VC = vehicle control.

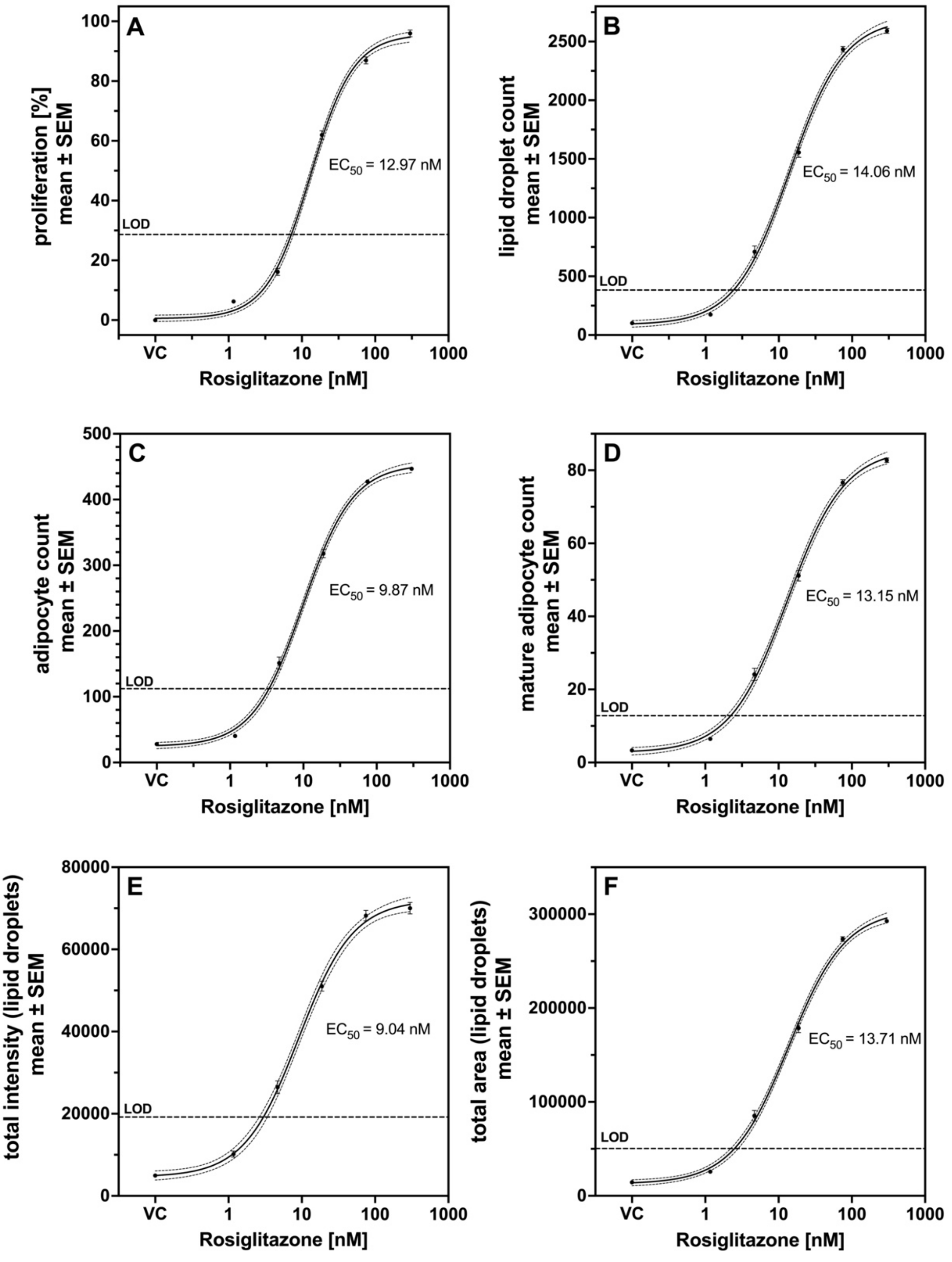

Figure S6. Dose-response relationship for the reference compound rosiglitazone in the adipogenesis assay with 6.25 nM dexamethasone in the differentiation medium. (A) proliferation normalized on the mean of the vehicle control, (B) lipid droplet count per field, (C) adipocyte count per field, (D) mature adipocyte count per field, (E) total intensity of the NileRed staining within the lipid droplet mask per field and (F) total area occupied by lipid droplets per field. 160 or more replicates per concentration ($n \geq 160$). VC = vehicle control, LOD = limit of detection, RFU = relative fluorescence unit.

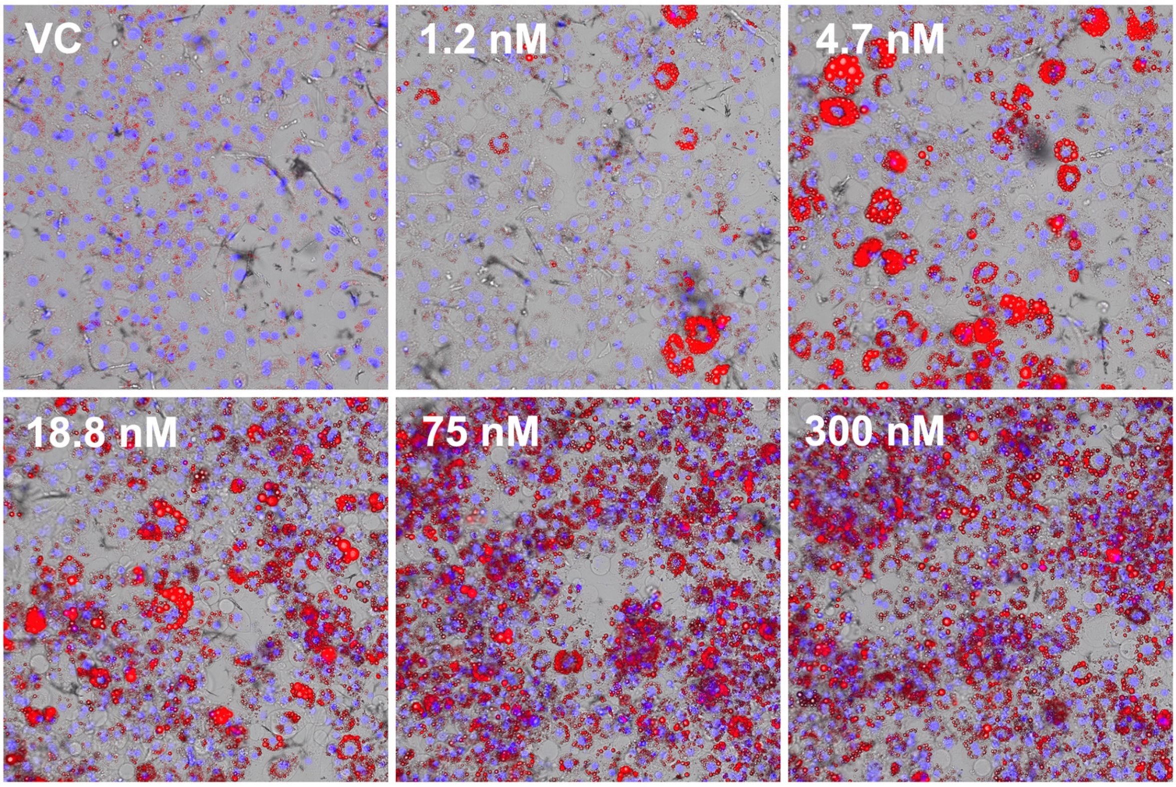

Figure S7. Dose-dependent induction of adipogenesis in 3T3-L1 cells exposed to the reference compound rosiglitazone. Merged brightfield and fluorescence images. Nuclei are stained with NucBlue (blue) and triglycerides with NileRed (red). VC = vehicle control. Raw pictures were processed in the same manner for visualization.

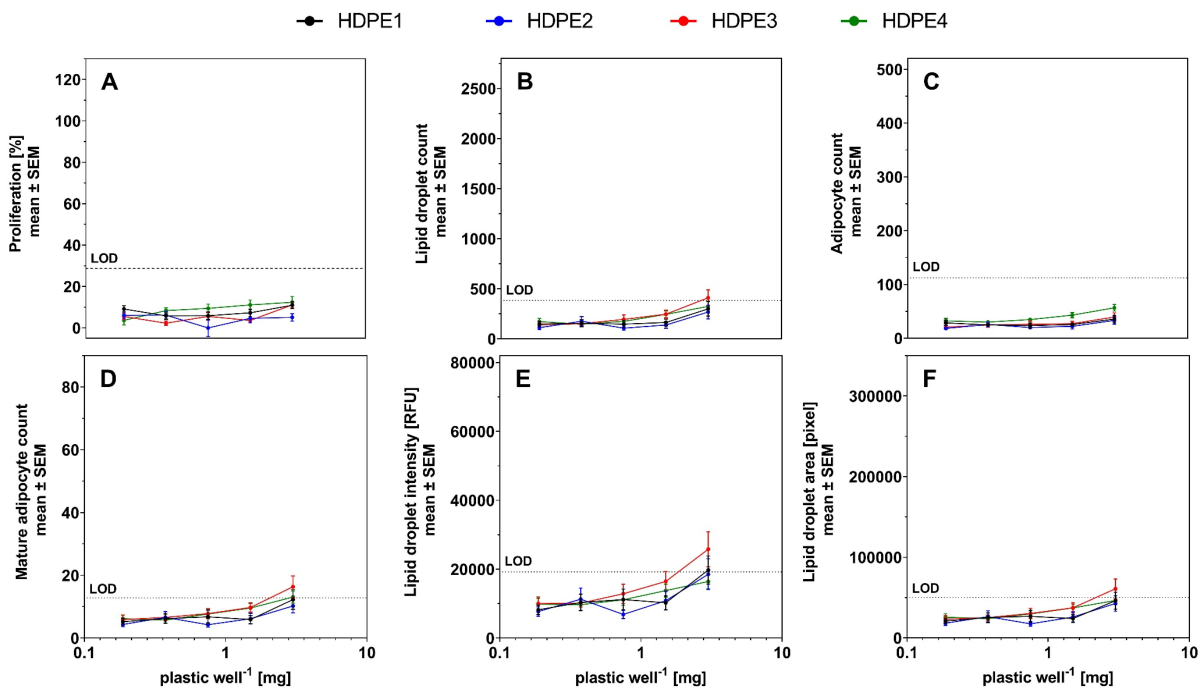

Figure S8. Dose-response relationship for the HDPE plastic extracts (HDPE 1–4) in the adipogenesis assay. (A) proliferation normalized on the mean of the vehicle control, (B) lipid droplet count per field, (C) adipocyte count per field, (D) mature adipocyte count per field, (E) total intensity of the NileRed staining within the lipid droplet mask per field and (F) total area occupied by lipid droplets per field. Twelve or more replicates per concentration ($n \geq 12$). LOD = limit of detection, RFU = relative fluorescence unit.

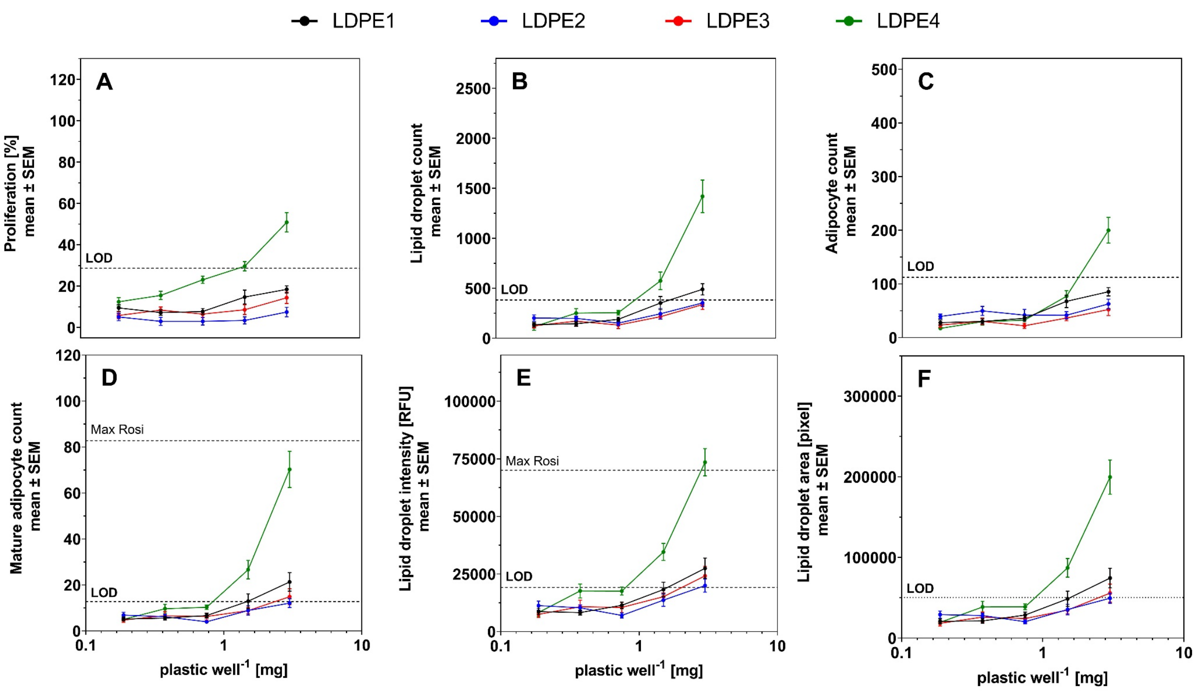

Figure S9. Dose-response relationship for the LDPE plastic extracts (LDPE 1–4) in the adipogenesis assay. (A) proliferation normalized on the mean of the vehicle control, (B) lipid droplet count per field, (C) adipocyte count per field, (D) mature adipocyte count per field, (E) total intensity of the NileRed staining within the lipid droplet mask per field and (F) total area occupied by lipid droplets per field. Twelve or more replicates per concentration ($n \geq 12$). LOD = limit of detection, Max Rosi = rosiglitazone maximal response, RFU = relative fluorescence unit.

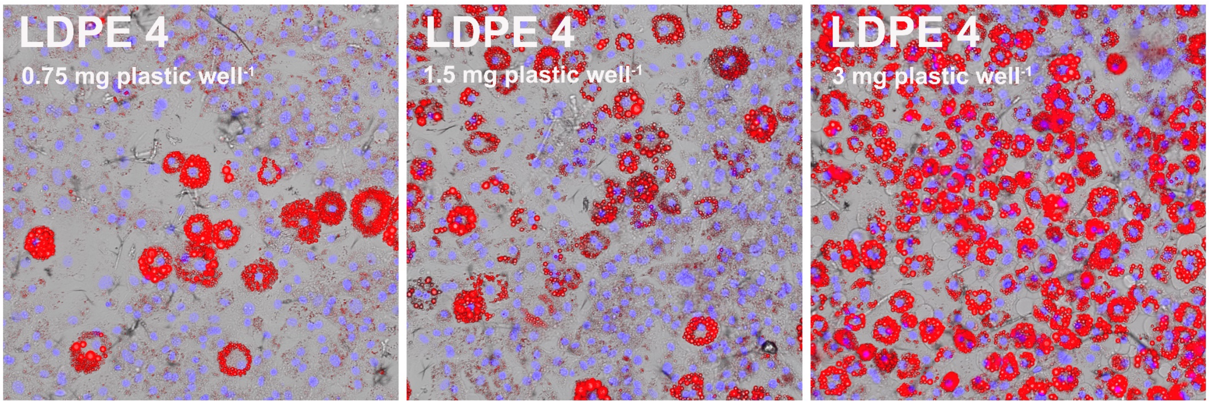

Figure S10. Dose-dependent induction of adipogenesis in 3T3-L1 cells exposed to the active plastic extract LDPE 4. Merged brightfield and fluorescence images. Nuclei are stained with NucBlue (blue) and triglycerides with NileRed (red). Raw pictures were processed in the same manner for visualization.

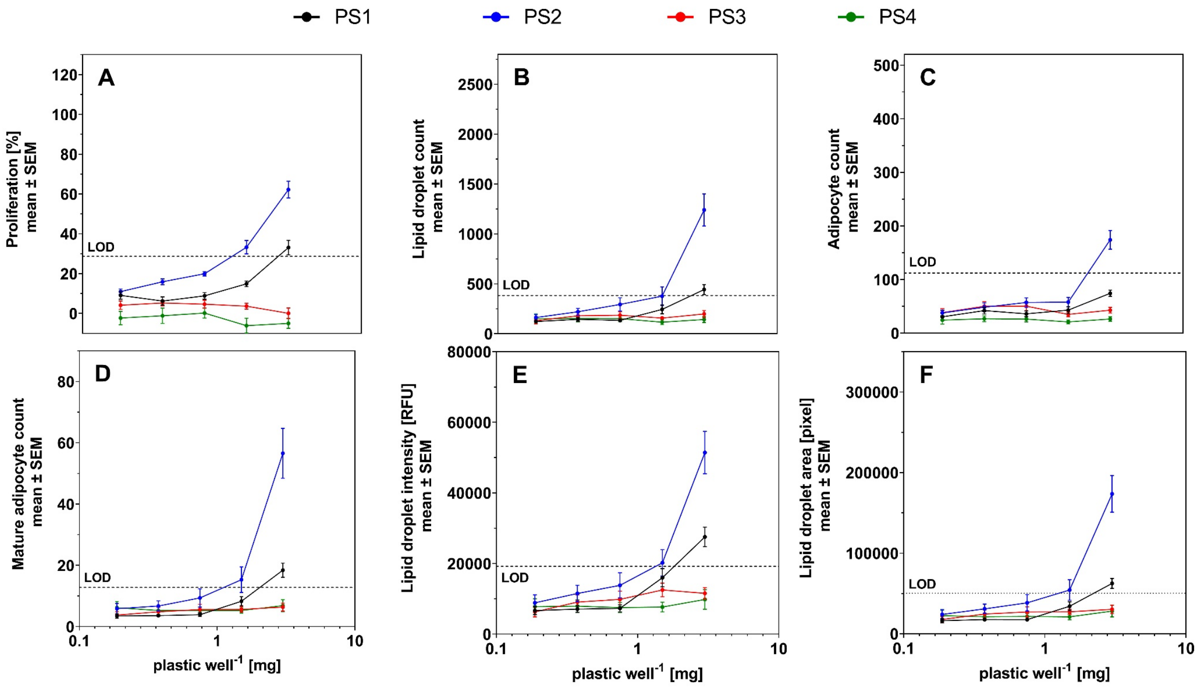

Figure S11. Dose-response relationships for the PS plastic extracts (PS 1–4) in the adipogenesis assay. (A) proliferation normalized on the mean of the vehicle control, (B) lipid droplet count per field, (C) adipocyte count per field, (D) mature adipocyte count per field, (E) total intensity of the NileRed staining within the lipid droplet mask per field and (F) total area occupied by lipid droplets per field. Twelve or more replicates per concentration ($n \geq 12$). LOD = limit of detection, RFU = relative fluorescence unit.

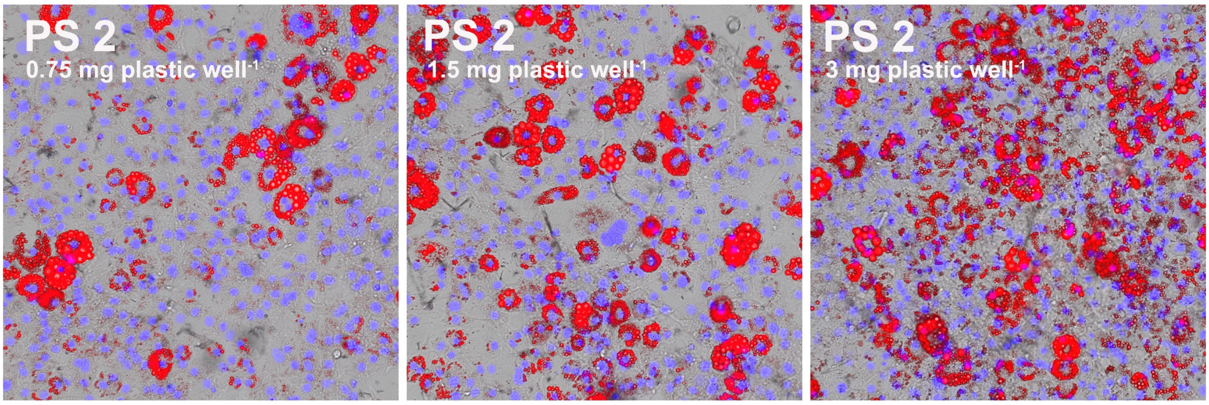

Figure S12. Dose-dependent induction of adipogenesis in 3T3-L1 cells exposed to the active plastic extract PS 2. Merged brightfield and fluorescence images. Nuclei are stained with NucBlue (blue) and triglycerides with NileRed (red). Raw pictures were processed in the same manner for visualization.

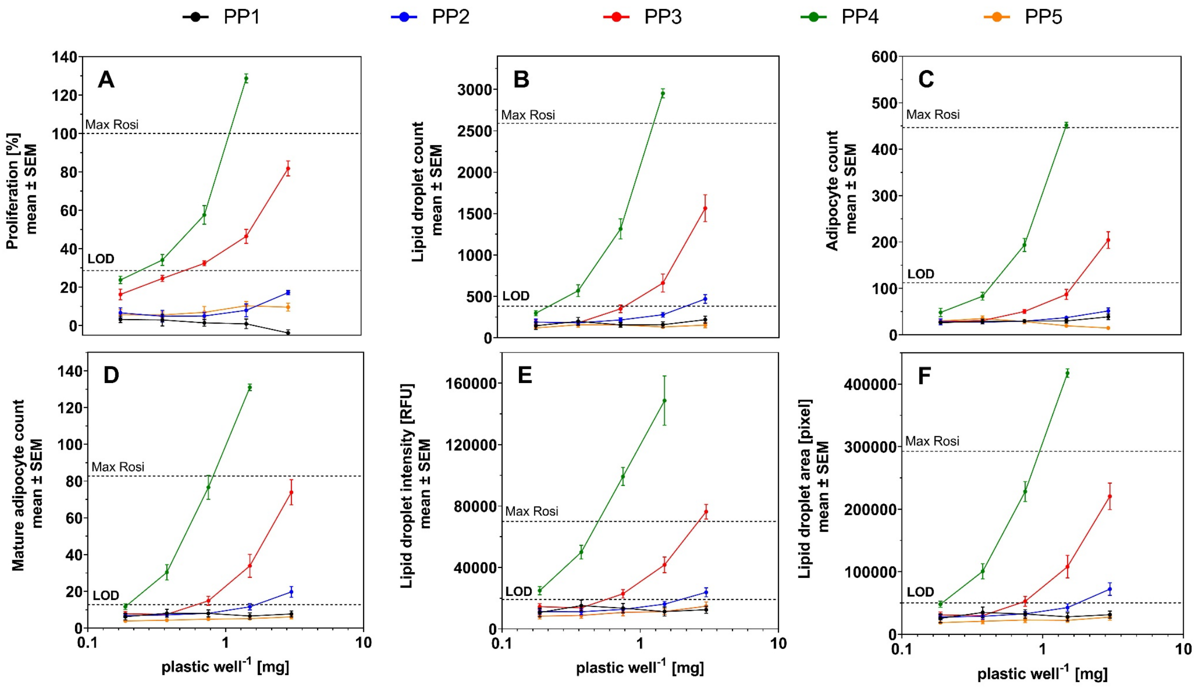

Figure S13. Dose-response relationship for the PP plastic extracts (PP 1–5) in the adipogenesis assay. (A) proliferation normalized on the mean of the vehicle control, (B) lipid droplet count per field, (C) adipocyte count per field, (D) mature adipocyte count per field, (E) total intensity of the NileRed staining within the lipid droplet mask per field and (F) total area occupied by lipid droplets per field. Twelve or more replicates per concentration ($n \geq 12$). LOD = limit of detection, Max Rosi = rosiglitazone maximal response, RFU = relative fluorescence unit.

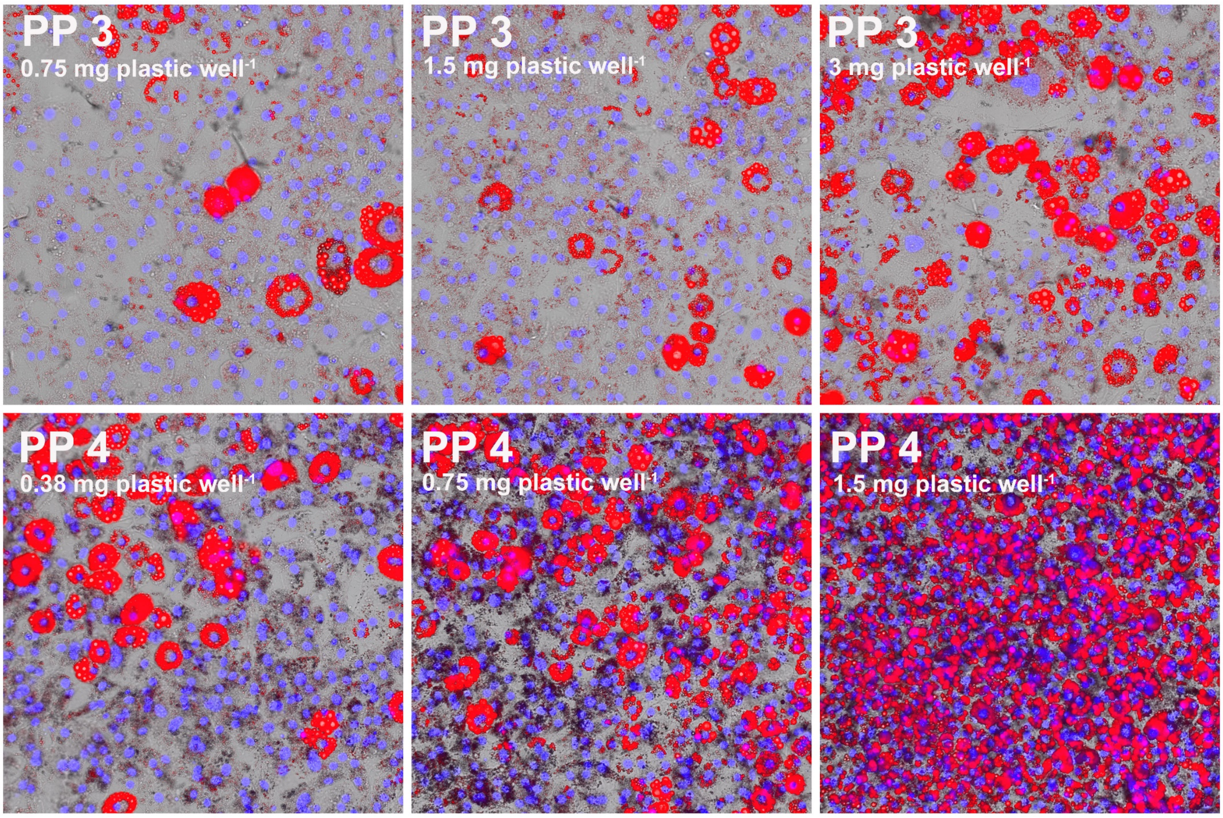

Figure S14. Dose-dependent induction of adipogenesis in 3T3-L1 cells exposed to the active plastic extracts PP 3 and PP 4. Merged brightfield and fluorescence images. Nuclei are stained with NucBlue (blue) and triglycerides with NileRed (red). Raw pictures were processed in the same manner for visualization.

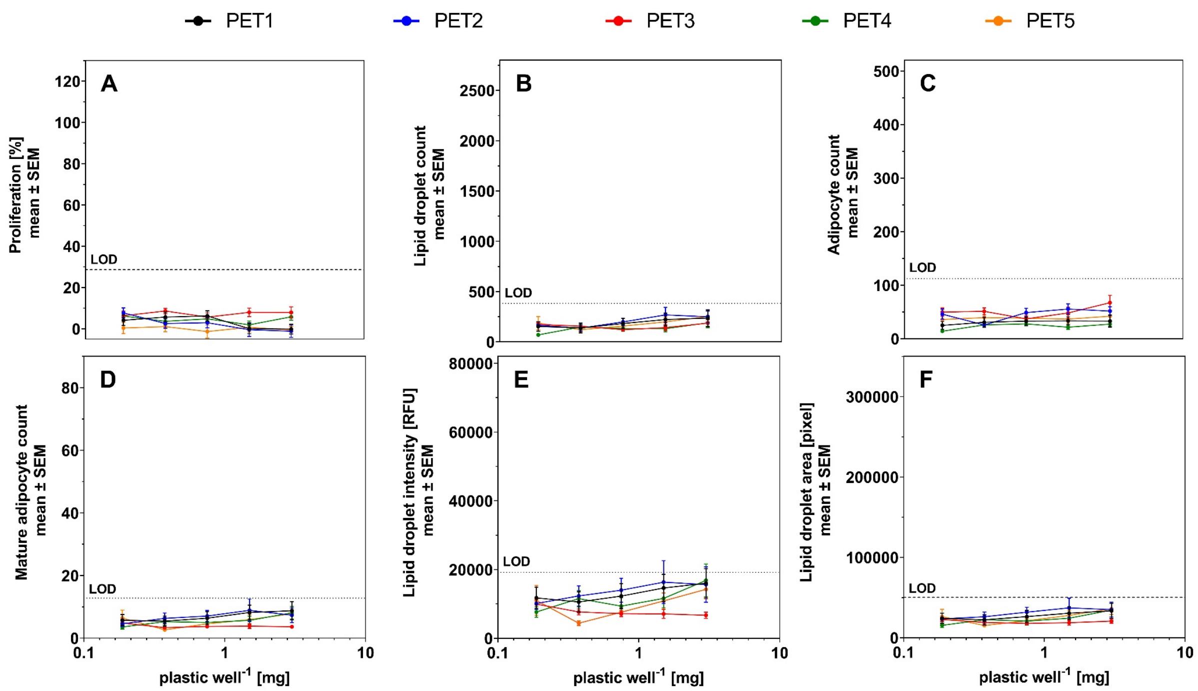

Figure S15. Dose-response relationship for the PET plastic extracts (PET 1–5) in the adipogenesis assay. (A) proliferation normalized on the mean of the vehicle control, (B) lipid droplet count per field, (C) adipocyte count per field, (D) mature adipocyte count per field, (E) total intensity of the NileRed staining within the lipid droplet mask per field and (F) total area occupied by lipid droplets per field. Twelve or more replicates per concentration ($n \geq 12$). LOD = limit of detection, RFU = relative fluorescence unit.

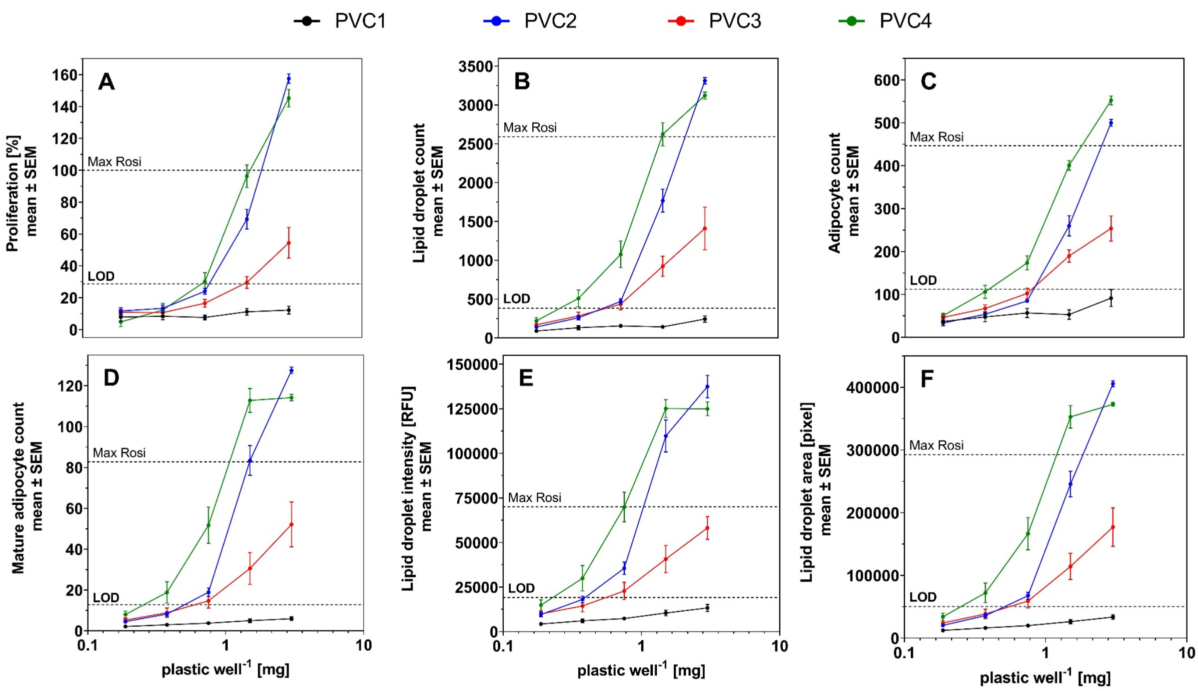

Figure S16. Dose-response relationship for the PVC plastic extracts (PVC 1–4) in the adipogenesis assay. (A) proliferation normalized on the mean of the vehicle control, (B) lipid droplet count per field, (C) adipocyte count per field, (D) mature adipocyte count per field, (E) total intensity of the NileRed staining within the lipid droplet mask per field and (F) total area occupied by lipid droplets per field. Twelve or more replicates per concentration (n ≥ 12). LOD = limit of detection, Max Rosi = rosiglitazone maximal response, RFU = relative fluorescence unit.

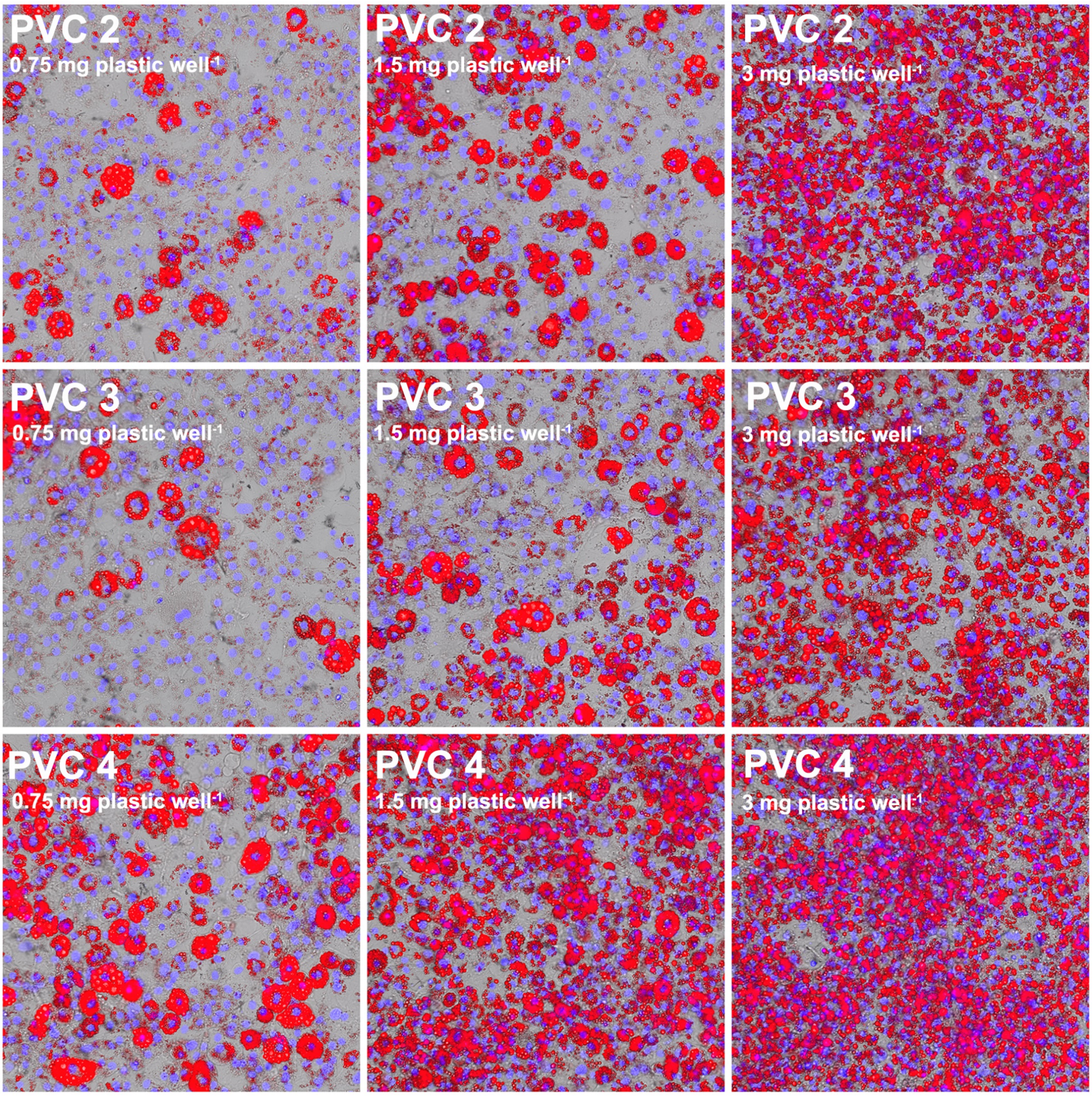

Figure S17. Dose-dependent induction of adipogenesis in 3T3-L1 cells exposed to the active plastic extracts PVC 2– 4. Merged brightfield and fluorescence images. Nuclei are stained with NucBlue (blue) and triglycerides with NileRed (red). Raw pictures were processed in the same manner for visualization.

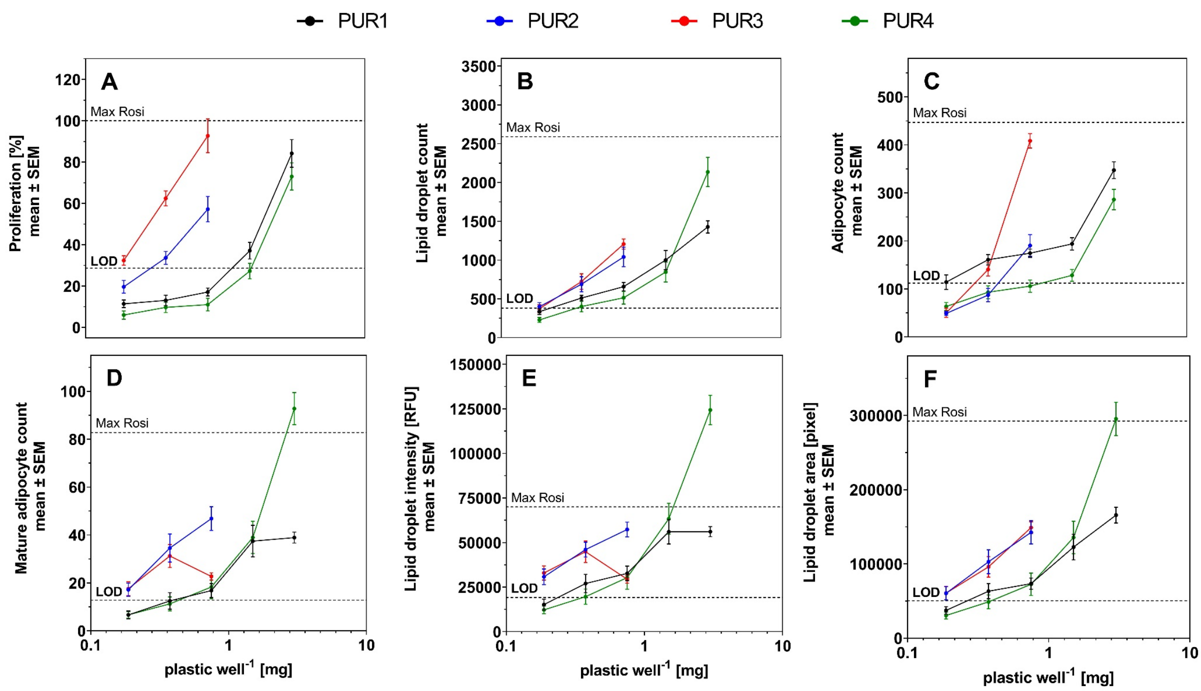

Figure S18. Dose-response relationship for the PUR plastic extracts (PUR 1–4) in the adipogenesis assay. (A) proliferation normalized on the mean of the vehicle control, (B) lipid droplet count per field, (C) adipocyte count per field, (D) mature adipocyte count per field, (E) total intensity of the NileRed staining within the lipid droplet mask per field and (F) total area occupied by lipid droplets per field. Twelve or more replicates per concentration (n ≥ 12). LOD = limit of detection, Max Rosi = rosiglitazone maximal response, RFU = relative fluorescence unit.

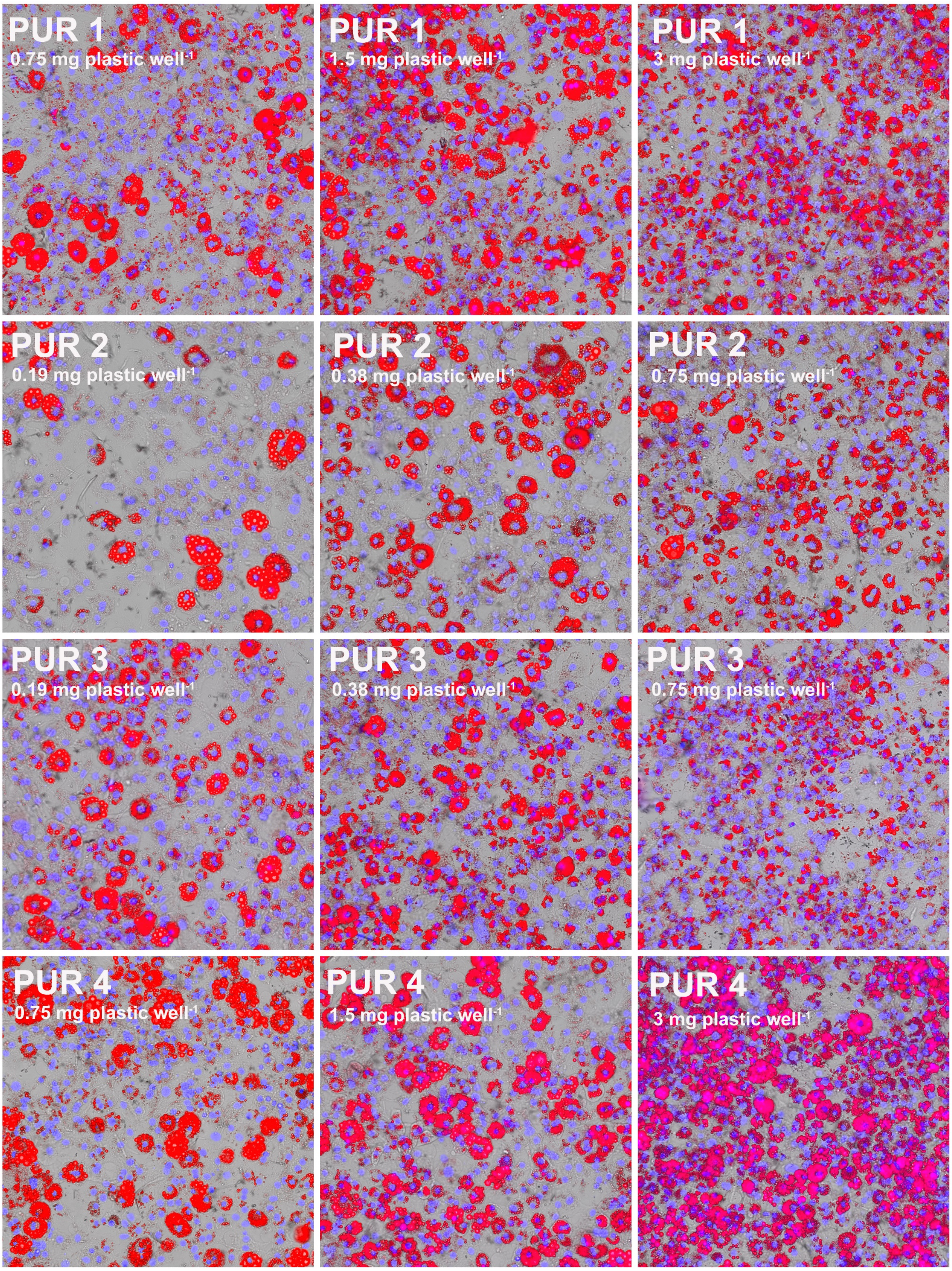

Figure S19. Dose-dependent induction of adipogenesis in 3T3-L1 cells exposed to the active plastic extracts PUR 1-4. Merged brightfield and fluorescence images. Nuclei are stained with NucBlue (blue) and triglycerides with NileRed (red). Raw pictures were processed in the same manner for visualization.

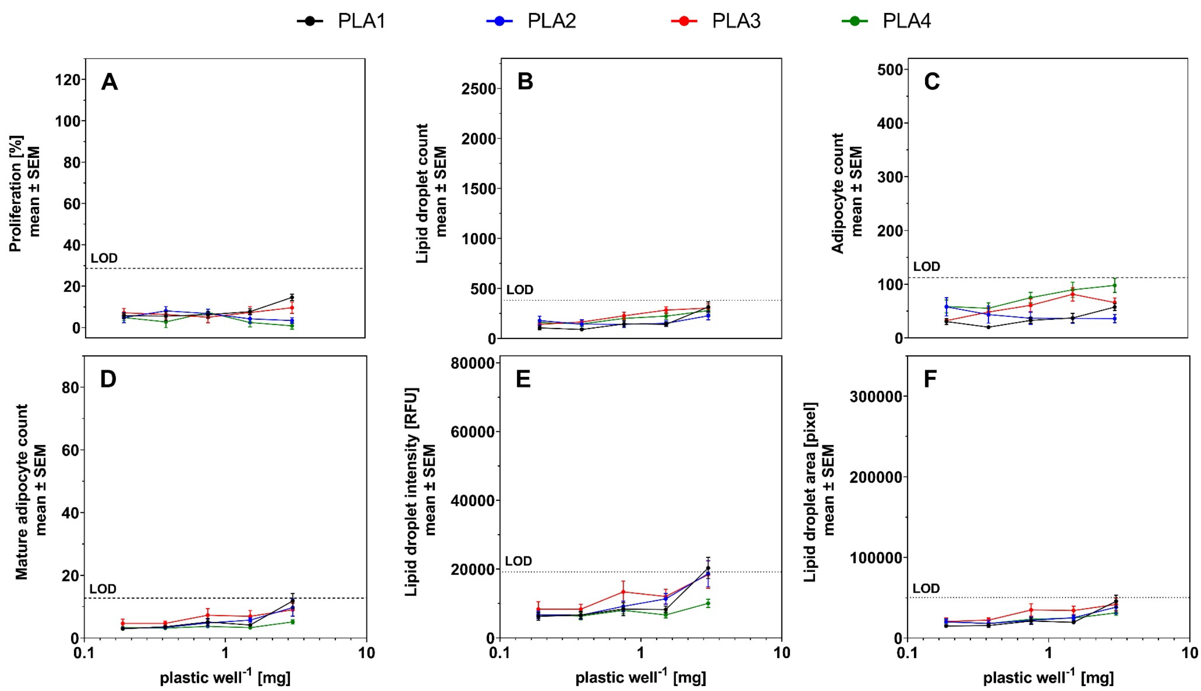

Figure S20. Dose-response relationship for the PLA plastic extracts (PLA 1–4) in the adipogenesis assay. (A) proliferation normalized on the mean of the vehicle control, (B) lipid droplet count per field, (C) adipocyte count per field, (D) mature adipocyte count per field, (E) total intensity of the NileRed staining within the lipid droplet mask per field and (F) total area occupied by lipid droplets per field. Twelve or more replicates per concentration ($n \geq 12$). LOD = limit of detection, Max Rosi = rosiglitazone maximal response, RFU = relative fluorescence unit.

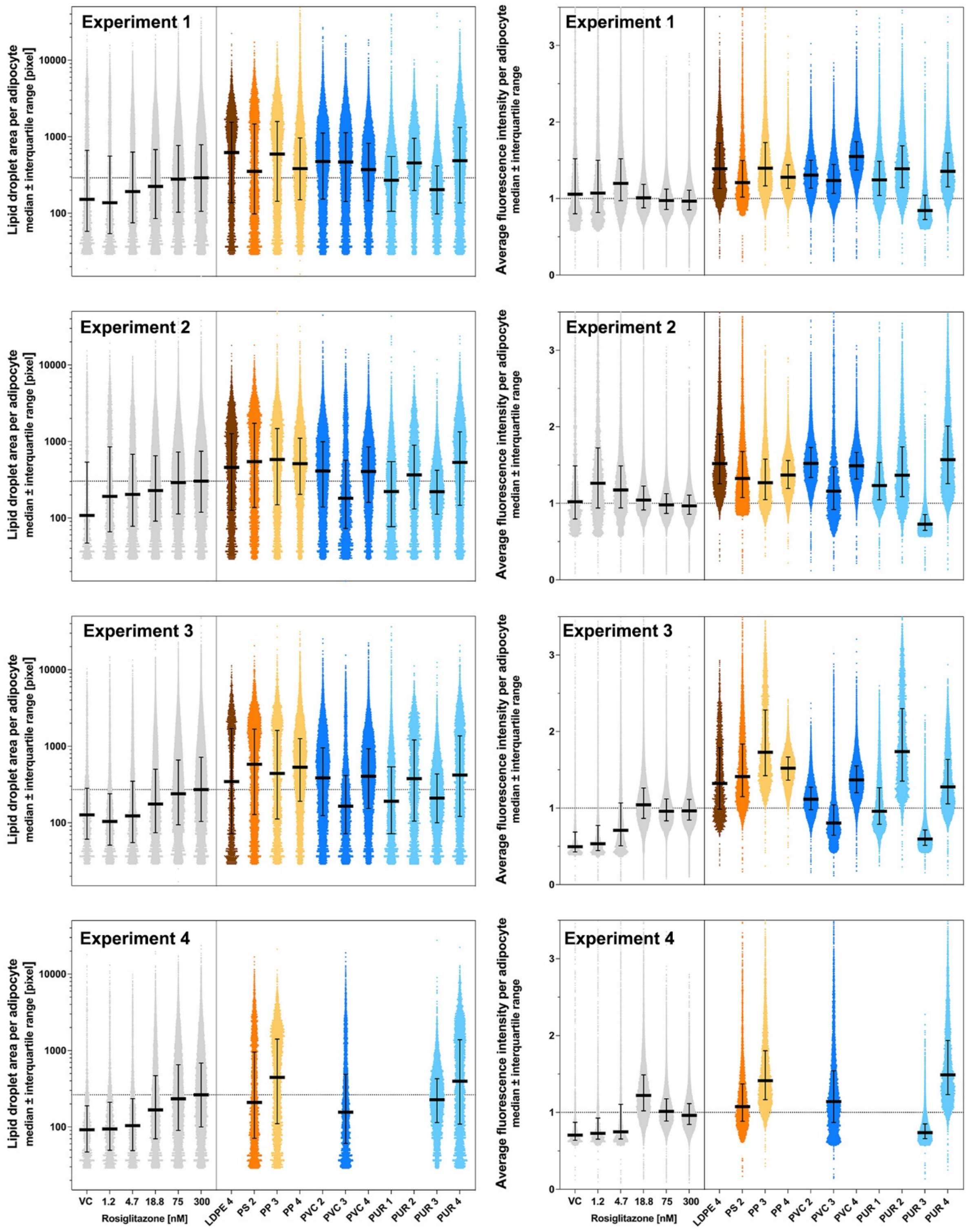

Figure S21. Size distribution of adipocyte population (left) and accumulation of triglyceride per adipocyte in cells exposed to rosiglitazone or the highest noncytotoxic concentration of the eleven active plastic extracts. Single-cell data from 4 independent experiments. Intensity data is normalized to the mean of the highest rosiglitazone concentration (300 nM). VC = vehicle control.

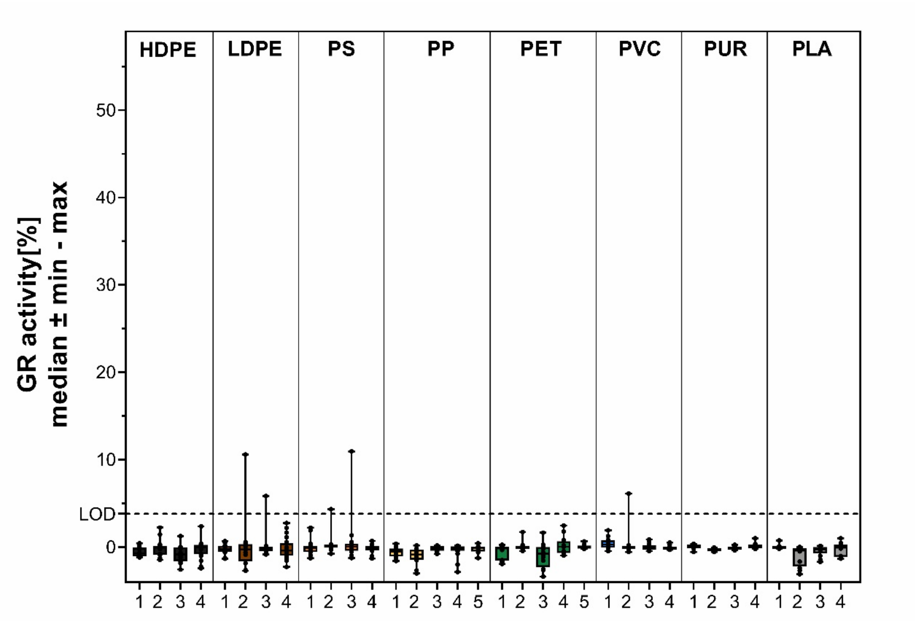

Figure S22. GR activity of plastic extracts at the highest noncytotoxic concentration. Highest noncytotoxic concentration was 1.5 mg plastic well-1 except for PP 4 (0.19 mg plastic well-1), PS 2 and PP 3 (0.38 mg plastic well-1), PLA 1, PVC 2 and PVC 4 (0.75 mg plastic well-1). GR = glucocorticoid receptor, LOD = limit of detection. n ≥ 12.

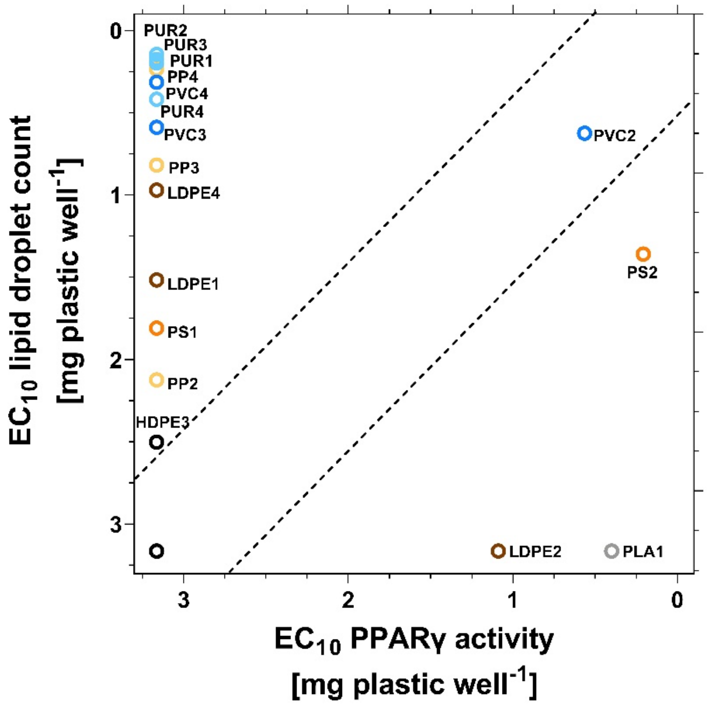

Figure S23. Correlation of the EC_{10} of the PPARγ activity and lipid droplet count.

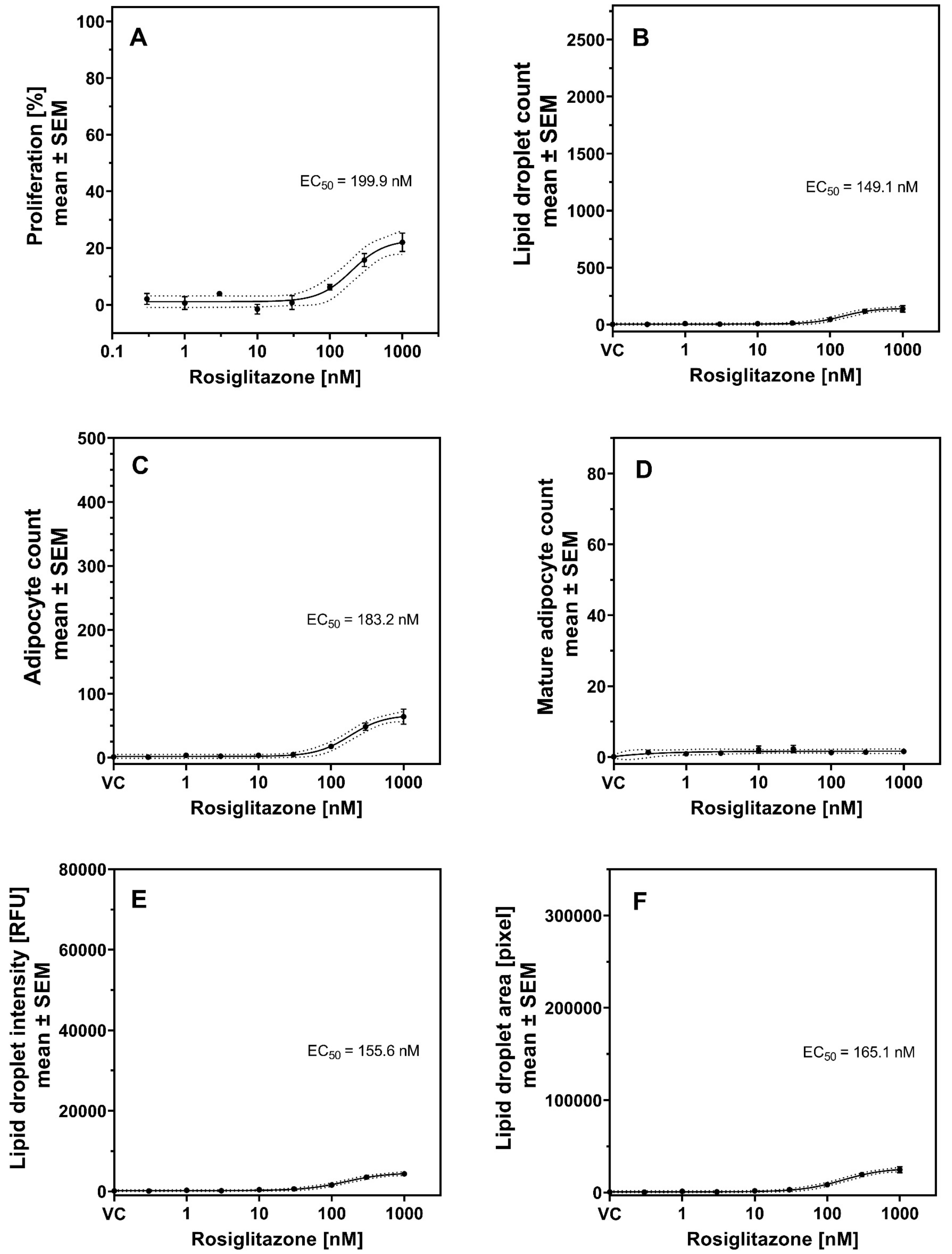

Figure S24. Dose-response relationship for the reference compound rosiglitazone in the adipogenesis assay without dexamethasone in the differentiation medium. (A) proliferation normalized on the mean of the vehicle control, (B) lipid droplet count per field, (C) adipocyte count per field, (D) mature adipocyte count per field, (E) total intensity of the NileRed staining within the lipid droplet mask per field and (F) total area occupied by lipid droplets per field. Eight or more replicates per concentration ($n \geq 8$). VC = vehicle control, RFU = relative fluorescence unit.

Table S1. List of all used chemicals and consumables.

chemical/consumable	supplier	further information
AdipoRed assay kit (NileRed)	Lonza	N3013
ATP (adenosine-5'-triphosphate disodium salt)	PanReac AppliChem	≥98 % (HPLC), CAS: 987655
Bovine calf serum iron-supplemented	Sigma	12138C
Charcoal-stripped fetal bovine serum	Gibco	12676029
CDTA (1,2-cyclohexanedinitrilotetraacetic acid)	Sigma	≥99 % (KT), CAS: 125572954
Dexamethasone	Sigma	≥98 % (HPLC), CAS: 50022
DMEM/F-12 with phenol red	Gibco	31331093
DMEM/F-12 without phenol red	Gibco	21041025
DMEM high glucose	Gibco	31966047
D-Luciferin, monopotassium salt	Thermo Scientific	≥99.7 % (HPLC), 88294
DL-dithiothreitol	Sigma	≥98 % (HPLC), CAS: 3483123
DMSO (dimethyl sulfoxide)	Sigma	≥99.5 % (GC), CAS: 67685
EDTA (ethylenediaminetetraacetic acid)	Sigma	≥99 % (titration), CAS: 60004
Fetal bovine serum	Gibco	10270-106
G 418 disulfate	Sigma	≥450 U/mg, CAS: 108321422
Glycerol	Sigma	≥99 % (GC), CAS: 56815
HEPES	VWR	L1613
IBMX (3-isobutyl-1-methylxanthine)	Sigma	≥99 % (HPLC), CAS: 28822587
Insulin, human recomb., zinc solution	Gibco	12585014, 27 U/mg
Magnesium carbonate hydroxide pentahydrate	Sigma	M5671, CAS: 56378724
Magnesium sulfate heptahydrate	Sigma	≥98 %, CAS: 10034998
Methanol	Sigma	≥99.8 % (HPLC), 322415
NucBlue live cell ready probes reagent	Life technologies	R37605
Penicillin/streptomycin	VWR	L0022-100
Rosiglitazone	Sigma	≥98 % (HPLC), CAS: 122320734
Sterile T75 cell culture flasks	Thermo Scientific	156499
Tris base	Sigma	≥99 % (titration), CAS: 77861
Triton™ X-100	Sigma	laboratory grade, CAS: 9002-93-1
96-well cell culture plates (black with transparent bottom)	Greiner	Cellstar 655090
384-well cell culture plates (white with transparent bottom)	Greiner	Cellstar 738-0062

Table S2. List of adipogenic chemicals based on the published literature.

Name	CAS	PubChem CID	References
1-850	251310 -57-3	2765122	4
2,4,6-tribromophenol	118-79-6	1483	15
2-ethylhexyl diphenyl phosphate	1241-94-7	14716	16
3,5,6-trichloro-2-pyridinol	6515-38-4	23017	17
3-tert-butyl-4-hydroxanisole	121-00-6	8456	13
4-hexyl phenol	2446-69-7	17132	18
4-n-octylphenol	1806-26-4	15730	19
4-nonylphenol	104-40-5	1752	19, 20
8:2 FTAcr	27905-45-9	119747	15
Acetamiprid	135410-20-7	213021	21
Acrylamide	79-06-1	6579	13
Allethrin	584-79-2	11442	22
Alpha naphthoflavone	604-59-1	11790	13
Azoxystrobin	131860-33-8	3034285	15
BDE-47	5436-43-1	95170	15, 23
Benzyl butyl phthalate	85-68-7	2347	24-26
Biphenthrin	82657-04-03	6442842	27
Bisphenol A	80-05-07	6623	21, 23-26, 28-33
Bisphenol A diglycidyl ether	1675-54-3	2286	31
Bisphenol F	620-92-8	12111	28, 29
Bisphenol S	80-09-1	6626	28-30, 32
Butylparaben	94-26-8	7184	26
Carboxymethylcellulose	9000-11-7	24748	13
Cetyl alcohol ethoxylate	5274-61-3	4303686	19
Chlorpyrifos	2921-88-2	2730	15, 17
Cypermethrin	52315-07-8	2912	15
Daidzein	486-66-8	5281708	34
DDE	72-55-9	3035	13
DDT	50-29-3	3036	13
Dechlorane plus	13560-89-9	26111	35
DEHP	117-81-7	8343	15, 36
Dexamethasone	50-02-2	5743	4, 37, 38
Diazinon	333-41-5	3017	39
Diethylene glycol dibenzoate	120-55-8	8437	6
Dibutyl phthalate	84-74-2	3026	15, 26
Dibutyltin	1002-53-5	6484	13
Dicamba	1918-00-9	3030	37
Diclofop-methyl	51338-27-3	39985	13

Name	CAS	PubChem CID	References
Di-iso-butyl phthalate	84-69-5	6782	[26]
Di-iso-decyl phthalate	89-16-7	33599	[6]
Di-iso-nonyl phthalate	28553-12-0	590836	[6]
Dioctyl sodium sulfosuccinate	577-11-7	23673837	[40, 41]
Diphenyl phosphate	838-85-7	13282	[42]
Ethylparaben	120-47-8	8434	[26]
Fenthion	55-38-9	3346	[22]
Fludioxonil	131341-86-1	86398	[21]
Fluoxastrobin	361377-29-9	11048796	[15]
Flusilazole	85509-19-9	73675	[21]
Flutamide	13311-84-7	3397	[4]
Forchlorfenuron	68157-60-8	93379	[21]
Genistein	446-72-0	5280961	[34]
Glyphosate	1071-83-6	3496	[13]
GW3965	405911-17-3	16078973	[4]
Halosulfuron-methyl	100784-20-1	91763	[13]
HBCD	3194-55-6	18529	[43]
Hexafluorobisphenol A	1478-61-1	73864	[4]
Imidacloprid	138261-41-3	86287518	[38]
Isopropylated triphenyl phosphate	78-30-8	6527	[44]
Isoxaflutole	141112-29-0	84098	[37]
Lactofen	77501-63-4	62276	[13]
Lauryl alcohol ethoxylate (4)	4536-30-5	24750	[19]
LG100268	153559-76-3	3922	[4]
MEHP	4376-20-9	20393	[36]
Methylparaben	99-76-3	7456	[26, 45]
Mono(2-ethylhexyl) phthalate	4376-20-9	20393	[46]
Monosodium glutamate	142-47-2	23672308	[13]
Musk xylene	81-15-2	62329	[26]
Nonylphenol ethoxylate (1-2)	9016-45-9	24773	[19]
Nonylphenol ethoxylate (20)	N/A	N/A	[19]
Nonylphenol ethoxylate (4)	N/A	N/A	[19]
Nonylphenol ethoxylate (6)	N/A	N/A	[19]
Nonylphenol ethoxylate (9-10)	N/A	N/A	[19]
Octylphenol ethoxylate (3)	2315-67-5	5590	[19]
P-80	9005-65-6	5284448	[13]
PBDE 99	60348-60-9	36159	[47]
PCB 153	35065-27-1	37034	[23]

Name	CAS	PubChem CID	References
Permethrin	52645-53-1	40326	15
PFHxS	3871-99-6	23678874	48
PFNA	375-95-1	67821	48
PFOA	335-67-1	9554	23, 48, 49
PFOS	1763-23-1	74483	48
Pioglitazone	112529-15-4	60560	22
Pirinixic acid	50892-23-4	5694	48
Prallethrin	23031-36-9	9839306	22
Propylparaben	94-13-3	7175	26, 45
Pymetrozine	123312-89-0	9576037	21
Pyraclostrobin	175013-18-0	6422843	15
Pyrimethanil	53112-28-0	91650	22
Quinoxyfen	124495-18-7	3391107	21, 22
Quizalofop-p-ethyl	100646-51-3	1617113	37
Retinoic acid	302-79-4	444795	22
Rosiglitazone	122320-73-4	77999	Positive control
Span 80	1338-43-8	9920342	41
Spirodiclofen	148477-71-8	177863	21
TBEP	78-51-3	6540	15
TBPH	26040-51-7	117291	15
TDBPIC	52434-90-9	103634	15
Tebuconazole	107534-96-3	86102	22
Tebupirimfos	96182-53-5	93516	21
Tert-butylphenyl diphenyl phosphate	56803-37-3	158333	15
Tetrabromobisphenol A	79-94-7	6618	2, 4, 22
Tetrachlorobisphenol A	79-95-8	6619	4
Tolyfluanid	731-27-1	12898	50
Tomadol 1-9	N/A	N/A	19
Tonalide	1506-02-1	89440	26
Tributyltin	687-73-3	3032732	4, 23, 26, 51-56
Triclocarban	101-20-2	7547	57
Tridecyl alcohol ethoxylate (9)	78330-21-9	N/A	19
Trifloxystrobin	141517-21-7	11665966	15
Triflumizole	68694-11-1	91699	21, 58
Tri-m-cresyl phosphate	563-04-2	11232	6
Tri-n-butyl phosphate	126-73-8	31357	15
Triphenyl phosphate	115-86-6	8289	15, 42, 44, 59
Triphenyltin	76-87-9	6327657	21, 22

Name	CAS	PubChem CID	References
Tris(4-tert-butylphenyl) phosphate	78-33-1	6530	[15]
Troglitazone	97322-87-7	5591	[23, 25, 44]
TTBP	732-26-3	12902	[22]
Zoxamide	156052-68-5	122087	[21]

Table S3. The chemicals tentatively identified using GC-QTOF-MS/MS (data from Zimmermann *et al.*)[14]

Sample	CAS	PubChem CID	Name according to NIST	Mass	Formula	Score
HDPE 1	1000374-06-1[a]	3023652	1,7-di-iso-propylnaphthalene	212.16	C16H20	71.58
	10233-13-3	25068	Dodecanoic acid, 1-methylethyl ester	242.23	C15H30O2	72.54
	138345-00-3	605776	7,9-Di-tertbutyl-1-oxaspiro[4,5]deca-6,9-dien-8-one	262.19	C17H26O2	70.98
	24169-43-5	595960	1,3-Dioxolane, 2-(bromomethyl)-2-(2-methylphenyl)-	256.01	C11H13BrO2	72.61
	87-97-8	6911	Phenol, 2,6-bis(1,1-dimethylethyl)-4-(methoxymethyl)-	250.19	C16H26O2	70.81
HDPE 2	4337-65-9	20342	Hexanedioic acid, mono(2-ethylhexyl)ester	258.18	C14H26O4	82.22
	6386-38-5	62603	Benzenepropanoic acid, 3,5-bis(1,1-dimethylethyl)-4-hydroxy-, methyl ester	292.20	C18H28O3	71.02
HDPE 3	96-76-4	7311	2,4-Di-tert-butylphenol	206.17	C14H22O	80.64
HDPE 4	1000192-65-0[a]	543423	1,2-15,16-Diepoxyhexadecane	254.23	C16H30O2	72.96
	1000315-44-3[a]	6423518	Phthalic acid, isobutyl tridec-2-yn-1-yl ester	400.26	C25H36O4	72.55
	1000336-60-4[a]	44515574	i-Propyl 12-methyl-tridecanoate	270.26	C17H34O2	73.73
	1000374-06-1[a]	3023652	1,7-di-iso-propylnaphthalene	212.16	C16H20	79.77
	101-86-0	7585	Octanal, 2-(phenylmethylene)-	216.15	C15H20O	85.79
	105794-58-9	537071	1-Heptatriacotanol	536.59	C37H76O	70.03
	13466-78-9	26049	3-Carene	136.13	C10H16	92.31
	16507-61-2	5367784	cis-1-Chloro-9-octadecene	286.24	C18H35Cl	74.15
	242794-76-9	564746	Bicyclo[5.2.0]nonane, 2-methylene-4,8,8-trimethyl-4-vinyl-	204.19	C15H24	85.25
	294-62-2	9268	Cyclododecane	168.19	C12H24	74.42
	499-97-8	68140	Cyclohexane, 1-methylene-4-(1-methylethenyl)-	136.13	C10H16	88.01
	535-77-3	10812	Benzene, 1-methyl-3-(1-methylethyl)-	134.11	C10H14	84.43
	546-80-5	261491	Bicyclo[3.1.0]hex-2-ene, 2-methyl-5-(1-methylethyl)-	136.13	C10H16	89.31
	586-63-0	102443	Cyclohexene, 3-methyl-6-(1-methylethylidene)-	136.13	C10H16	85.52
	5989-27-5	440917	D-Limonene	136.13	C10H16	91.24
LDPE 1	1000333-58-3[a]	12541027	cis-13-Octadecenoic acid, methyl ester	296.27	C19H36O2	75.47
	1000336-50-5[a]	13908974	Methyl 9-eicosenoate	324.30	C21H40O2	72.97
	1000364-37-1[a]	91738699	2-Methyl-3,5-dinitrobenzyl alcohol, TBDMS derivative	326.13	C14H22N2O5Si	71.01
	1000374-06-1[a]	3023652	1,7-di-iso-propylnaphthalene	212.16	C16H20	74.02
	1000374-17-9[a]	6428435	7-epi-cis-sesquisabinene hydrate	222.20	C15H26O	72.79
	10436-08-5	547891	cis-11-Eicosenamide	309.30	C20H39NO	77.21
	10436-09-6	5365369	trans-13-Docosenamide	337.33	C22H43NO	84.90
	10482-56-1	443162	L-.alpha.-Terpineol	154.14	C10H18O	87.36
	105794-58-9	537071	1-Heptatriacotanol	536.59	C37H76O	70.34
	1120-25-8	643801	9-Hexadecenoic acid, methyl ester, (Z)-	268.24	C17H32O2	74.59
	112-63-0	5284421	9,12-Octadecadienoic acid (Z,Z)-, methyl ester	294.26	C19H34O2	89.17
	112-80-1	445639	Oleic Acid	282.26	C18H34O2	70.26

Sample	CAS	PubChem CID	Name according to NIST	Mass	Formula	Score
	115-99-1	61040	1,6-Octadien-3-ol, 3,7-dimethyl-, formate	182.13	C11H18O2	81.34
	13466-78-9	26049	3-Carene	136.13	C10H16	82.42
	1632-73-1	15406	Fenchol	154.14	C10H18O	80.75
	1732-10-1	15612	Nonanedioic acid, dimethyl ester	216.14	C11H20O4	76.33
	17367-08-7	549041	Ethanol, 2-(9,12-octadecadienyloxy)-, (Z,Z)-	310.29	C20H38O2	71.76
	2091-29-4	4668	9-Hexadecenoic acid	254.23	C16H30O2	72.15
	562-74-3	11230	Terpinen-4-ol	154.14	C10H18O	82.74
	56554-30-4	556196	7,10,13-Hexadecatrienoic acid, methyl ester	264.21	C17H28O2	83.82
	56630-69-4	61268	13-Docosenoic acid, methyl ester	352.33	C23H44O2	90.77
	57156-97-5	5365571	12,15-Octadecadienoic acid, methyl ester	294.26	C19H34O2	89.69
	5989-27-5	440917	D-Limonene	136.13	C10H16	82.93
	60-33-3	5280450	9,12-Octadecadienoic acid (Z,Z)-	280.24	C18H32O2	72.77
	81601-03-8	91694967	Geranyl oleate	418.38	C28H50O2	75.52
LDPE 2	112-62-9	5364509	9-Octadecenoic acid (Z)-, methyl ester	296.27	C19H36O2	87.08
	5129-56-6	554144	Undecanoic acid, 10-methyl-, methyl ester	214.19	C13H26O2	83.16
	5129-58-8	21204	Tridecanoic acid, 12-methyl-, methyl ester	242.23	C15H30O2	73.08
	5129-61-3	110444	Heptadecanoic acid, 16-methyl-, methyl ester	298.29	C19H38O2	74.37
	57156-97-5	5365571	12,15-Octadecadienoic acid, methyl ester	294.26	C19H34O2	88.65
	6386-38-5	62603	Benzenepropanoic acid, 3,5-bis(1,1-dimethylethyl)-4-hydroxy-, methyl ester	292.20	C18H28O3	73.29
	96-76-4	7311	2,4-Di-tert-butylphenol	206.17	C14H22O	82.88
LDPE 3	6386-38-5	62603	Benzenepropanoic acid, 3,5-bis(1,1-dimethylethyl)-4-hydroxy-, methyl ester	292.20	C18H28O3	89.02
	82304-66-3	545303	7,9-Di-tert-butyl-1-oxaspiro(4,5)deca-6,9-diene-2,8-dione	276.17	C17H24O3	82.82
	96-76-4	7311	2,4-Di-tert-butylphenol	206.17	C14H22O	81.84
LDPE 4	1000131-11-6[a]	5362676	Z,Z,Z-1,4,6,9-Nonadecatetraene	260.25	C19H32	73.26
	1000131-33-2[a]	5363633	Z-(13,14-Epoxy)tetradec-11-en-1-ol acetate	268.20	C16H28O3	70.02
	1000142-34-3[a]	591964	2-Adamantanol, 2-(bromomethyl)-	244.05	C11H17BrO	73.95
	1000192-65-0[a]	543423	1,2-15,16-Diepoxyhexadecane	254.23	C16H30O2	73.92
	1000314-35-6[a]	6421281	(E,Z,Z)-2,4,7-Tridecatrienal	192.15	C13H20O	70.48
	1000336-36-7[a]	14122946	Methyl 4,7,10,13-hexadecatetraenoate	262.19	C17H26O2	72.54
	1000336-38-4[a]	91694372	Methyl 3-cis,9-cis,12-cis-octadecatrienoate	292.24	C19H32O2	71.26
	1000336-60-4[a]	44515574	i-Propyl 12-methyl-tridecanoate	270.26	C17H34O2	73.43
	1000352-68-4[a]	88368751	Oleyl alcohol, trifluoroacetate	364.26	C20H35F3O2	75.37
	1000374-06-1[a]	3023652	1,7-di-iso-propylnaphthalene	212.16	C16H20	79.77
	1000374-18-0[a]	11972555	.alpha.-acorenol	222.20	C15H26O	86.08
	1000405-59-5[a]	91701181	Fumaric acid, 2-octyl tridec-2-yn-1-yl ester	406.31	C25H42O4	71.10
	1000406-96-9[a]	91697642	Undec-10-ynoic acid, tridec-2-yn-1-yl ester	360.30	C24H40O2	74.05
	1000414-43-3[a]	91694966	Geranyl palmitoleate	390.35	C26H46O2	75.56

Sample	CAS	PubChem CID	Name according to NIST	Mass	Formula	Score
	10287-53-3	25127	Parbenate	193.11	C11H15NO2	83.57
	10482-56-1	443162	L-.alpha.-Terpineol	154.14	C10H18O	87.52
	105794-58-9	537071	1-Heptatriacotanol	536.59	C37H76O	78.17
	115-99-1	61040	1,6-Octadien-3-ol, 3,7-dimethyl-, formate	182.13	C11H18O2	83.17
	117066-77-0	none	2-((2S,4aR)-4a,8-Dimethyl-1,2,3,4,4a,5,6,7-octahydronaphthalen-2-yl)propan-2-ol	222.20	C15H26O	85.50
	120-51-4	2345	Benzyl Benzoate	212.08	C14H12O2	70.07
	1209-71-8	6432005	2-Naphthalenemethanol, 1,2,3,4,4a,5,6,7-octahydro-.alpha.,.alpha.,4a,8-tetramethyl-, (2R-cis)-	222.20	C15H26O	87.40
	13466-78-9	26049	3-Carene	136.13	C10H16	89.92
	177205-54-8	622707	1,2,3,4-Tetrahydro-1,4-ethanoanthracene, 9,10-dimethoxy-	268.15	C18H20O2	81.18
	17735-94-3	5312518	cis-13-Eicosenoic acid	310.29	C20H38O2	70.56
	1845-30-3	164888	cis-Verbenol	152.12	C10H16O	82.59
	194607-96-0	527206	2-((4aS,8R,8aR)-4a,8-Dimethyl-3,4,4a,5,6,7,8,8a-octahydronaphthalen-2-yl)propan-2-ol	222.20	C15H26O	85.06
	21747-46-6	10910653	1H-Cycloprop[e]azulene, 1a,2,3,5,6,7,7a,7b-octahydro-1,1,4,7-tetramethyl-, [1aR-(1a.alpha.,7.alpha.,7a.beta.,7b.alpha.)]-	204.19	C15H24	82.27
	22117-09-5	5367371	5,8,11-Heptadecatrien-1-ol	250.23	C17H30O	72.18
	2244-16-8	16724	D-Carvone	150.10	C10H14O	78.01
	29141-10-4	6553885	(1R,2R,5S)-5-Methyl-2-(prop-1-en-2-yl)cyclohexanol	154.14	C10H18O	80.60
	294-62-2	9268	Cyclododecane	168.19	C12H24	73.79
	29803-82-5	122485	2-Cyclohexen-1-ol, 1-methyl-4-(1-methylethyl)-, cis-	154.14	C10H18O	70.29
	353313[a]	17868	Bicyclo[3.1.0]hex-2-ene, 2-methyl-5-(1-methylethyl)-	136.13	C10H16	87.39
	463-40-1	5280934	9,12,15-Octadecatrienoic acid, (Z,Z,Z)-	278.23	C18H30O2	73.20
	470-40-6	11401461	cis-Thujopsene	204.19	C15H24	79.33
	499-97-8	68140	Cyclohexane, 1-methylene-4-(1-methylethenyl)-	136.13	C10H16	89.08
	506-26-3	5280933	Gamolenic acid	278.23	C18H30O2	73.07
	514-95-4	578237	1,5,5-Trimethyl-6-methylene-cyclohexene	136.13	C10H16	76.22
	535-77-3	10812	Benzene, 1-methyl-3-(1-methylethyl)-	134.11	C10H14	84.97
	5392-40-5	8843	Citral	152.12	C10H16O	80.01
	562-74-3	11230	Terpinen-4-ol	154.14	C10H18O	83.75
	56666-38-7	41961	2H-Pyran, tetrahydro-2-(12-pentadecynyloxy)-	308.27	C20H36O2	72.69
	584-79-2	11442	Bioallethrin	302.19	C19H26O3	71.11
	5989-27-5	440917	D-Limonene	136.13	C10H16	83.74
	61465-23-4	577045	(+-)-trans-1-Isopropenyl-4-methyl-1,4-cyclohexanediol	170.13	C10H18O2	71.12
	7212-40-0	12618691	2-Cyclohexen-1-ol, 1-methyl-4-(1-methylethenyl)-, trans-	152.12	C10H16O	72.18
	7452-79-1	24020	Butanoic acid, 2-methyl-, ethyl ester	130.10	C7H14O2	76.76
	77-53-2	65575	Cedrol	222.20	C15H26O	90.06
	7785-70-8	82227	(1R)-2,6,6-Trimethylbicyclo[3.1.1]hept-2-ene	136.13	C10H16	82.07

Sample	CAS	PubChem CID	Name according to NIST	Mass	Formula	Score
	7786-67-6	24585	Cyclohexanol, 5-methyl-2-(1-methylethenyl)-	154.14	C10H18O	81.53
	99-87-6	7463	p-Cymene	134.11	C10H14	84.23
PS 1	100-42-5	7501	Styrene	104.06	C8H8	92.48
	131758-71-9	562543	(2,3-Diphenylcyclopropyl)methyl phenyl sulfoxide, trans-	332.12	C22H20OS	76.37
	20071-09-4	11954175	Benzene, 1,1'-(1,2-cyclobutanediyl)bis-, trans-	208.13	C16H16	83.78
	25558-23-0	568889	Cyclobutane, 1,3-diphenyl-, trans-	208.13	C16H16	71.07
	538-81-8	641683	1,3-Butadiene, 1,4-diphenyl-, (E,E)-	206.11	C16H14	78.93
PS 2	1000192-89-2[a]	5375831	Thiocarbamic acid, N,N-dimethyl, S-1,3-diphenyl-2-butenyl ester	311.13	C19H21NOS	72.30
	1000336-60-4[a]	44515574	i-Propyl 12-methyl-tridecanoate	270.26	C17H34O2	81.81
	100-42-5	7501	Styrene	104.06	C8H8	86.69
	10436-08-5	547891	cis-11-Eicosenamide	309.30	C20H39NO	75.46
	120-51-4	2345	Benzyl Benzoate	212.08	C14H12O2	73.31
	20071-09-4	11954175	Benzene, 1,1'-(1,2-cyclobutanediyl)bis-, trans-	208.13	C16H16	74.04
	23470-00-0	123409	Hexadecanoic acid, 2-hydroxy-1-(hydroxymethyl)ethyl ester	330.28	C19H38O4	81.59
	29422-13-7	34581	Naphthalene, 1,2,3,4-tetrahydro-2-phenyl-	208.13	C16H16	72.68
	301-02-0	5283387	9-Octadecenamide, (Z)-	281.27	C18H35NO	87.13
	56728-02-0	609923	Benzene, 1,1'-[2-methyl-2-(phenylthio)cyclopropylidene]bis-	316.13	C22H20S	70.10
PS 3	100-42-5	7501	Styrene	104.06	C8H8	89.70
	131758-71-9	562543	(2,3-Diphenylcyclopropyl)methyl phenyl sulfoxide, trans-	332.12	C22H20OS	76.42
	20071-09-4	11954175	Benzene, 1,1'-(1,2-cyclobutanediyl)bis-, trans-	208.13	C16H16	86.62
	538-81-8	641683	1,3-Butadiene, 1,4-diphenyl-, (E,E)-	206.11	C16H14	80.62
	56728-02-0	609923	Benzene, 1,1'-[2-methyl-2-(phenylthio)cyclopropylidene]bis-	316.13	C22H20S	77.61
PS 4	1000130-80-7[a]	5363617	E-11-Methyl-12-tetradecen-1-ol acetate	268.24	C17H32O2	77.16
	1000130-81-0[a]	549821	11,13-Dimethyl-12-tetradecen-1-ol acetate	282.26	C18H34O2	78.57
	1000192-65-0[a]	543423	1,2-15,16-Diepoxyhexadecane	254.23	C16H30O2	73.64
	1000374-06-1[a]	3023652	1,7-di-iso-propylnaphthalene	212.16	C16H20	72.97
	1000382-54-3[a]	91693137	Carbonic acid, eicosyl vinyl ester	368.33	C23H44O3	75.68
	1000406-16-9[a]	91692473	Undec-10-ynoic acid, hexadecyl ester	406.38	C27H50O2	75.59
	100-41-4	7500	Ethylbenzene	106.08	C8H10	89.77
	100-42-5	7501	Styrene	104.06	C8H8	89.77
	150-86-7	5280435	Phytol	296.31	C20H40O	77.15
	17634-51-4	561243	1,3,5-Cycloheptatriene, 7-ethyl-	120.09	C9H12	72.65
	20071-09-4	11954175	Benzene, 1,1'-(1,2-cyclobutanediyl)bis-, trans-	208.13	C16H16	86.94
	56728-02-0	609923	Benzene, 1,1'-[2-methyl-2-(phenylthio)cyclopropylidene]bis-	316.13	C22H20S	80.56
	5989-27-5	440917	D-Limonene	136.13	C10H16	78.77
	98-82-8	7406	Benzene, (1-methylethyl)-	120.09	C9H12	81.26
PP 1	n.d.					

Sample	CAS	PubChem CID	Name according to NIST	Mass	Formula	Score
PP 2	n.d.					
PP 3	1000336-43-6[a]	20619411	Methyl 8-methyl-nonanoate	186.16	C11H22O2	77.31
	1000339-14-5[a]	91695412	Fumaric acid, 2-ethylhexyl undecyl ester	382.31	C23H42O4	70.28
	1000368-53-5[a]	91205583	Ethyl stearate, 9,12-diepoxy	340.26	C20H36O4	72.53
	1000381-53-1[a]	91726212	Succinic acid, 2-(2-chlorophenoxy)ethyl ethyl ester	300.08	C14H17ClO5	71.07
	109-43-3	7986	Decanedioic acid, dibutyl ester	314.25	C18H34O4	82.40
	111-11-5	8091	Octanoic acid, methyl ester	158.13	C9H18O2	82.15
	128-37-0	31404	Butylated Hydroxytoluene	220.18	C15H24O	87.82
	24560-98-3	119250	Oxiraneoctanoic acid, 3-octyl-, cis-	298.25	C18H34O3	71.24
	33368-86-4	10186592	2-(Octanoyloxy)propane-1,3-diyl bis(decanoate)	526.42	C31H58O6	73.10
	33368-87-5	10436013	2-(Decanoyloxy)propane-1,3-diyl dioctanoate	498.39	C29H54O6	73.20
	4098-71-9	169132	Isophorone diisocyanate	222.14	C12H18N2O2	80.96
	5129-61-3	110444	Heptadecanoic acid, 16-methyl-, methyl ester	298.29	C19H38O2	80.72
	628-97-7	12366	Hexadecanoic acid, ethyl ester	284.27	C18H36O2	75.72
	6386-38-5	62603	Benzenepropanoic acid, 3,5-bis(1,1-dimethylethyl)-4-hydroxy-, methyl ester	292.20	C18H28O3	74.23
	77-90-7	6505	Tributyl acetylcitrate	402.23	C20H34O8	76.77
	87-97-8	6911	Phenol, 2,6-bis(1,1-dimethylethyl)-4-(methoxymethyl)-	250.19	C16H26O2	77.23
PP 4	1000336-62-4[a]	53745103	i-Propyl 14-methyl-pentadecanoate	298.29	C19H38O2	79.06
	101-68-8	7570	Benzene, 1,1'-methylenebis[4-isocyanato-	250.07	C15H10N2O2	90.58
	10436-09-6	5365369	trans-13-Docosenamide	337.33	C22H43NO	72.31
	301-02-0	5283387	9-Octadecenamide, (Z)-	281.27	C18H35NO	85.30
	77-90-7	6505	Tributyl acetylcitrate	402.23	C20H34O8	84.50
PP 5	1000333-58-3[a]	12541027	cis-13-Octadecenoic acid, methyl ester	296.27	C19H36O2	74.59
	1000336-60-4[a]	44515574	i-Propyl 12-methyl-tridecanoate	270.26	C17H34O2	86.61
	1000351-75-2[a]	14574254	Eicosyl trifluoroacetate	394.31	C22H41F3O2	80.57
	1000368-56-5[a]	91691599	2-Hexyldodecyl isobutyrate	340.33	C22H44O2	80.81
	1000374-06-1[a]	3023652	1,7-di-iso-propylnaphthalene	212.16	C16H20	71.86
	1000382-54-3[a]	91693137	Carbonic acid, eicosyl vinyl ester	368.33	C23H44O3	70.61
	103-95-7	517827	3-(4-Isopropylphenyl)-2-methylpropionaldehyde	190.14	C13H18O	87.10
	112-39-0	8181	Hexadecanoic acid, methyl ester	270.26	C17H34O2	85.30
	119-61-9	3102	Benzophenone	182.07	C13H10O	82.23
	1222-05-5	91497	Cyclopenta[g]-2-benzopyran, 1,3,4,6,7,8-hexahydro-4,6,6,7,8,8-hexamethyl-	258.20	C18H26O	94.81
	128-37-0	31404	Butylated Hydroxytoluene	220.18	C15H24O	89.19
	13491-79-7	26068	Cyclohexanol, 2-(1,1-dimethylethyl)-	156.15	C10H20O	78.36
	2425-77-6	95337	1-Decanol, 2-hexyl-	242.26	C16H34O	79.62
	5129-56-6	554144	Undecanoic acid, 10-methyl-, methyl ester	214.19	C13H26O2	76.80
	5129-58-8	21204	Tridecanoic acid, 12-methyl-, methyl ester	242.23	C15H30O2	79.44

Sample	CAS	PubChem CID	Name according to NIST	Mass	Formula	Score
	5129-61-3	110444	Heptadecanoic acid, 16-methyl-, methyl ester	298.29	C19H38O2	89.70
	5348-82-3	79299	Acetic acid, chloro-, octadecyl ester	346.26	C20H39ClO2	79.89
	55741-10-1	615306	Naphthalene, 6,7-diethyl-1,2,3,4-tetrahydro-1,1,4,4-tetramethyl-	244.22	C18H28	78.47
	6386-38-5	62603	Benzenepropanoic acid, 3,5-bis(1,1-dimethylethyl)-4-hydroxy-, methyl ester	292.20	C18H28O3	76.72
	7460-74-4	81964	Pentanoic acid, 2-phenylethyl ester	206.13	C13H18O2	75.64
	80-54-6	228987	Lilial	204.15	C14H20O	81.43
	88-29-9	6930	7-Acetyl-6-ethyl-1,1,4,4-tetramethyltetralin	258.20	C18H26O	78.53
PET 1	5989-27-5	440917	D-Limonene	136.13	C10H16	86.85
PET 2	n.d.					
PET 3	n.d.					
PET 4	n.d.					
PET 5	n.d.					
PVC 1	1000043-05-3[a]	6452096	Ethyl iso-allocholate	436.32	C26H44O5	70.14
	1000368-53-5[a]	91205583	Ethyl stearate, 9,12-diepoxy	340.26	C20H36O4	82.01
	1000383-37-7[a]	5354568	Glycidyl oleate	338.28	C21H38O3	75.46
	111-03-5	5283468	9-Octadecenoic acid (Z)-, 2,3-dihydroxypropyl ester	356.29	C21H40O4	70.61
	119-61-9	3102	Benzophenone	182.07	C13H10O	76.29
	14290-23-4	539937	Myristin, 1,3-diaceto-2-	386.27	C21H38O6	79.34
	17598-94-6	33979	Dodecanoic acid, 1-(hydroxymethyl)-1,2-ethanediyl ester	456.38	C27H52O5	72.34
	26719-54-0	537376	Dodecanoic acid 3-dodecanoyloxy-propyl ester	440.39	C27H52O4	71.92
	4337-65-9	20342	Hexanedioic acid, mono(2-ethylhexyl)ester	258.18	C14H26O4	76.61
	5129-56-6	554144	Undecanoic acid, 10-methyl-, methyl ester	214.19	C13H26O2	81.77
	5129-61-3	110444	Heptadecanoic acid, 16-methyl-, methyl ester	298.29	C19H38O2	81.70
	52380-33-3	5364432	11-Octadecenoic acid, methyl ester	296.27	C19H36O2	82.24
	55268-70-7	191981	Hexadecanoic acid, 2,3-bis(acetyloxy)propyl ester	414.30	C23H42O6	72.01
	55429-68-0	539928	Eicosanoic acid, 2-(acetyloxy)-1-[(acetyloxy)methyl]ethyl ester	470.36	C27H50O6	73.40
	645-66-9	69527	Lauric anhydride	382.35	C24H46O3	73.38
	761-35-3	99931	Hexadecanoic acid, 1-(hydroxymethyl)-1,2-ethanediyl ester	568.51	C35H68O5	71.87
PVC 2	1000324-52-2[a]	91718008	Adipic acid, isohexyl methyl ester	244.17	C13H24O4	74.23
	1000339-40-5[a]	12151622	1,2-Cyclohexanedicarboxylic acid, dinonyl ester	424.36	C26H48O4	80.59
	1000339-74-3[a]	91721826	1,2-Cyclohexanedicarboxylic acid, cyclohexylmethyl nonyl ester	394.31	C24H42O4	78.08
	1000339-85-1[a]	91721974	1,2-Cyclohexanedicarboxylic acid, 3,5-dimethylcyclohexyl nonyl ester	408.32	C25H44O4	75.36
	109-39-7	66959	2-Butoxyethyl oleate	382.35	C24H46O3	78.43
	112-62-9	5364509	9-Octadecenoic acid (Z)-, methyl ester	296.27	C19H36O2	80.99
	117-81-7	8343	Bis(2-ethylhexyl) phthalate	390.28	C24H38O4	72.28
	128-37-0	31404	Butylated Hydroxytoluene	220.18	C15H24O	86.70
	29761-21-5	34697	Phosphoric acid, isodecyl diphenyl ester	390.20	C22H31O4P	74.21

Sample	CAS	PubChem CID	Name according to NIST	Mass	Formula	Score
	301-02-0	5283387	9-Octadecenamide, (Z)-	281.27	C18H35NO	81.87
	4337-65-9	20342	Hexanedioic acid, mono(2-ethylhexyl)ester	258.18	C14H26O4	76.59
PVC 3	1000115-60-4[a]	5369409	5-Hexadecenoic acid, 2-methoxy-, methyl ester	298.25	C18H34O3	71.33
	1000127-49-8[a]	590850	Phen-1,4-diol, 2,3-dimethyl-5-trifluoromethyl-	206.06	C9H9F3O2	72.01
	1000131-33-2[a]	5363633	Z-(13,14-Epoxy)tetradec-11-en-1-ol acetate	268.20	C16H28O3	71.95
	1000215-67-6[a]	none	trans-2,4-Dimethylthiane, S,S-dioxide	162.07	C7H14O2S	73.76
	1000215-75-3[a]	none	trans-2-methyl-4-n-pentylthiane, S,S-dioxide	218.13	C11H22O2S	82.26
	1000253-26-1[a]	569846	Octanediamide, N,N'-di-benzoyloxy-	412.16	C22H24N2O6	71.67
	1000270-36-9[a]	569440	Benzamide, N-(1,3-dihydro-2-oxo-4-isobenzofuryl)-	253.07	C15H11NO3	79.15
	1000324-49-0[a]	91713297	Adipic acid, 2-ethylhexyl tetradecyl ester	454.40	C28H54O4	70.35
	1000333-54-0[a]	15717634	17-Octadecynoic acid, methyl ester	294.26	C19H34O2	70.63
	1000333-58-3[a]	12541027	cis-13-Octadecenoic acid, methyl ester	296.27	C19H36O2	86.84
	1000340-22-6[a]	9814973	Benzoic acid, tridecyl ester	304.24	C20H32O2	76.91
	1000340-22-7[a]	64671	Benzoic acid, tetradecyl ester	318.26	C21H34O2	81.9
	1000352-68-4[a]	88368751	Oleyl alcohol, trifluoroacetate	364.26	C20H35F3O2	79.42
	1000356-41-5[a]	91724604	Isophthalic acid, butyl 10-chlorodecyl ester	396.21	C22H33ClO4	71.83
	1000367-89-7[a]	90471467	Benzoic acid, dec-2-yl ester	262.19	C17H26O2	71.93
	1000368-75-3[a]	91711800	Benzoic acid, 10-chlorodecyl ester	296.15	C17H25ClO2	72.25
	1000371-47-7[a]	21262075	1-Nonylcycloheptane	224.25	C16H32	74.28
	1000377-71-8[a]	91719631	Phthalic acid, nonyl oct-3-yl ester	404.29	C25H40O4	82.23
	1000406-16-5[a]	91692432	Undec-10-ynoic acid, dodecyl ester	350.32	C23H42O2	71.23
	10417-94-4	446284	cis-5,8,11,14,17-Eicosapentaenoic acid	302.23	C20H30O2	73.46
	105794-58-9	537071	1-Heptatriacotanol	536.59	C37H76O	78.32
	108511-83-7	569871	2-Benzoyloxy-1,1,10-trimethyl-6,9-epidioxydecalin	330.18	C20H26O4	71.18
	1129-41-5	14322	Carbamic acid, methyl-, 3-methylphenyl ester	165.08	C9H11NO2	80.66
	115-89-9	8291	Diphenyl methyl phosphate	264.06	C13H13O4P	78.24
	119-61-9	3102	Benzophenone	182.07	C13H10O	80.07
	120-46-7	8433	Dibenzoylmethane	224.08	C15H12O2	73.54
	128-37-0	31404	Butylated Hydroxytoluene	220.18	C15H24O	82.89
	139776-09-3	569530	S-Benzoyl-N-(p-nitrobenzylidene)thiohydroxylamine	286.04	C14H10N2O3S	74.31
	143-07-7	3893	Dodecanoic acid	200.18	C12H24O2	78.34
	149180-87-0	624073	Butylaldehyde, 4-benzyloxy-4-[2,2,-dimethyl-4-dioxolanyl]-	278.15	C16H22O4	72.53
	150-86-7	5280435	Phytol	296.31	C20H40O	78.92
	177746-99-5	91692548	Methyl 15-hydroxy-9,12-octadecadienoate	310.25	C19H34O3	76.43
	20548-62-3	590836	Phthalic acid, bis(7-methyloctyl) ester	418.31	C26H42O4	78.82
	22599-96-8	22213932	Cholestan-3-ol, 2-methylene-, (3.beta.,5.alpha.)-	400.37	C28H48O	73.41
	24560-98-3	119250	Oxiraneoctanoic acid, 3-octyl-, cis-	298.25	C18H34O3	77.99

Sample	CAS	PubChem CID	Name according to NIST	Mass	Formula	Score
	25360-09-2	19107815	tert-Hexadecanethiol	258.24	C16H34S	72.32
	2676-41-7	146287	6,9,12-Octadecatrienoic acid, methyl ester	292.24	C19H32O2	74.8
	26896-20-8	62838	Neodecanoic acid	172.15	C10H20O2	72.5
	28108-99-8	34148	Phosphoric acid, (1-methylethyl)phenyl diphenyl ester	368.12	C21H21O4P	78.89
	29761-21-5	34697	Phosphoric acid, isodecyl diphenyl ester	390.20	C22H31O4P	73.13
	334-68-9	9548	Dodecane, 1-fluoro-	188.19	C12H25F	74.35
	33795-18-5	214694	Phosphonic acid, (p-hydroxyphenyl)-	174.01	C6H7O4P	76.11
	3443-82-1	5365676	9,12-Octadecadienoic acid (Z,Z)-, 2-hydroxy-1-(hydroxymethyl)ethyl ester	354.28	C21H38O4	79.87
	34909-69-8	631942	Phosphoric acid, bis(4-methylphenyl) phenyl ester	354.10	C20H19O4P	83.97
	373-49-9	445638	Palmitoleic acid	254.24	C16H30O2	77.31
	56051-53-7	554084	Cyclopropanebutanoic acid, 2-[[2-[[2-[(2-pentylcyclopropyl)methyl]cyclopropyl]methyl]cyclopropyl]methyl]-, methyl ester	374.32	C25H42O2	75.58
	57-10-3	985	n-Hexadecanoic acid	256.24	C16H32O2	74.41
	57156-91-9	42151	2,5-Octadecadiynoic acid, methyl ester	290.23	C19H30O2	71.56
	60-33-3	5280450	9,12-Octadecadienoic acid (Z,Z)-	280.24	C18H32O2	74.4
	60609-53-2	5364688	8-Hexadecenal, 14-methyl-, (Z)-	252.25	C17H32O	77.49
	74685-30-6	5364600	5-Eicosene, (E)-	280.32	C20H40	80.86
	76841-70-8	71403428	E-2-Hexenyl benzoate	204.12	C13H16O2	73.99
	77-90-7	6505	Tributyl acetylcitrate	402.23	C20H34O8	71.72
	78-31-9	6528	Phosphoric acid, 4-methylphenyl diphenyl ester	340.09	C19H17O4P	79.33
	816-19-3	102491	Hexanoic acid, 2-ethyl-, methyl ester	158.13	C9H18O2	71.83
	82304-66-3	545303	7,9-Di-tert-butyl-1-oxaspiro(4,5)deca-6,9-diene-2,8-dione	276.17	C17H24O3	71.69
	84-77-5	6788	Didecyl phthalate	446.34	C28H46O4	83.9
	85763-57-1	33865	11-Methyldodecanol	200.21	C13H28O	78.32
PVC 4	1225365[a]	520263	Phosphoric acid, 2-methylphenyl diphenyl ester	340.09	C19H17O4P	86.56
	1919690[a]	81591	Benzoic acid, heptyl ester	220.15	C14H20O2	84.53
	1000308-89-8[a]	3024584	Phthalic acid, decyl nonyl ester	432.32	C27H44O4	70.78
	1000339-40-5[a]	12151622	1,2-Cyclohexanedicarboxylic acid, dinonyl ester	424.36	C26H48O4	74.42
	1000339-74-3[a]	91721826	1,2-Cyclohexanedicarboxylic acid, cyclohexylmethyl nonyl ester	394.31	C24H42O4	77.87
	1000340-22-6[a]	9814973	Benzoic acid, tridecyl ester	304.24	C20H32O2	80.46
	1000353-65-9[a]	91714177	Adipic acid, 3-heptyl tetradecyl ester	440.39	C27H52O4	76.38
	1000367-91-3[a]	103653	Benzoic acid, 2-methylbutyl ester	192.12	C12H16O2	77.5
	1000368-69-4[a]	243678	Benzoic acid, hept-2-yl ester	220.15	C14H20O2	76.04
	1000371-07-7[a]	91719575	Phthalic acid, 5-methylhex-2-yl heptadecyl ester	502.40	C32H54O4	75.96
	18699-48-4	29218	1,4-Benzenedicarboxylic acid, bis(2-methylpropyl) ester	278.15	C16H22O4	90.68
	20548-62-3	590836	Phthalic acid, bis(7-methyloctyl) ester	418.31	C26H42O4	81.45
	29761-21-5	34697	Phosphoric acid, isodecyl diphenyl ester	390.20	C22H31O4P	80.26

Sample	CAS	PubChem CID	Name according to NIST	Mass	Formula	Score
	34909-69-8	631942	Phosphoric acid, bis(4-methylphenyl) phenyl ester	354.10	C20H19O4P	88.72
	59736-57-1	570433	Benzoic acid 2-methylpentyl ester	206.13	C13H18O2	83.13
	76841-70-8	71403428	E-2-Hexenyl benzoate	204.12	C13H16O2	73.45
	77-90-7	6505	Tributyl acetylcitrate	402.23	C20H34O8	83.62
	78-31-9	6528	Phosphoric acid, 4-methylphenyl diphenyl ester	340.09	C19H17O4P	83.33
	84-64-0	6779	1,2-Benzenedicarboxylic acid, butyl cyclohexyl ester	304.17	C18H24O4	75.60
	84-76-4	6787	1,2-Benzenedicarboxylic acid, dinonyl ester	418.31	C26H42O4	83.79
	85-68-7	2347	Benzyl butyl phthalate	312.14	C19H20O4	71.83
	94-50-8	66751	Benzoic acid, octyl ester	234.16	C15H22O2	85.23
PUR 1	112-61-8	8201	Methyl stearate	298.29	C19H38O2	75.92
	128-37-0	31404	Butylated Hydroxytoluene	220.18	C15H24O	89.33
PUR 2	1000128-20-5[a]	none	(+,-)-Epi-perhydrohistrionicotoxin	295.29	C19H37NO	71.38
	1000195-87-0[a]	550132	8,14-Seco-3,19-epoxyandrostane-8,14-dione, 17-acetoxy-3.beta.-methoxy-4,4-dimethyl-	420.25	C24H36O6	70.01
	1000303-02-6[a]	6423312	7-Amino-1,3-dihydro-indol-2-one	148.06	C8H8N2O	75.44
	128-37-0	31404	Butylated Hydroxytoluene	220.18	C15H24O	92.86
	149-57-5	8697	Hexanoic acid, 2-ethyl-	144.12	C8H16O2	71.15
	2456-81-7	75567	Pyridine, 4-(1-pyrrolidinyl)-	148.10	C9H12N2	81.91
	2566-91-8	6451414	Oxiraneoctanoic acid, 3-octyl-, methyl ester, cis-	312.27	C19H36O3	76.43
	301-02-0	5283387	9-Octadecenamide, (Z)-	281.27	C18H35NO	80.39
	5129-61-3	110444	Heptadecanoic acid, 16-methyl-, methyl ester	298.29	C19H38O2	72.06
	584-84-9	11443	Benzene, 2,4-diisocyanato-1-methyl-	174.04	C9H6N2O2	85.39
	61338-98-5	547892	Benzeneethanamine, 2-fluoro-.beta.,3,4-trihydroxy-N-isopropyl-	229.11	C11H16FNO3	74.85
	76-25-5	6436	Triamcinolone Acetonide	434.21	C24H31FO6	70.57
	823-40-5	13205	1,3-Benzenediamine, 2-methyl-	122.08	C7H10N2	71.37
PUR 3	1000370-31-1[a]	458684	4,4'-Di-tert-butyl-diphenylamine	281.21	C20H27N	70.45
	1000370-31-3[a]	117942	Tert-octyldiphenylamine	281.21	C20H27N	79.04
	1000400-90-8[a]	291360	benzaldehyde, 4-(ethylphenylamino)-	225.12	C15H15NO	75.37
	1000408-33-2[a]	91739821	2-[2-Methoxy-5-(1,1,3,3-tetramethylbutyl)phenyl]-2H-benzotriazole	337.22	C21H27N3O	77.39
	6386-38-5	62603	Benzenepropanoic acid, 3,5-bis(1,1-dimethylethyl)-4-hydroxy-, methyl ester	292.20	C18H28O3	70.30
	78-40-0	6535	Triethyl phosphate	182.07	C6H15O4P	74.93
PUR 4	128-37-0	31404	Butylated Hydroxytoluene	220.18	C15H24O	77.47
	729-43-1	5484329	Ethanone, 1-phenyl-, (1-phenylethylidene)hydrazone	236.13	C16H16N2	81.99
PLA 1	1000382-54-3[a]	91693137	Carbonic acid, eicosyl vinyl ester	368.33	C23H44O3	78.36
	554-12-1	11124	Methyl propionate	88.05	C4H8O2	74.34
PLA 2	554-12-1	11124	Methyl propionate	88.05	C4H8O2	72.33
PLA 3	not analyzed via GC-QTOF-MS/MS					

Sample	CAS	PubChem CID	Name according to NIST	Mass	Formula	Score
PLA 4	112-67-4	8206	Palmitoyl chloride	274.21	C16H31ClO	70.25
	112-80-1	445639	Oleic Acid	282.26	C18H34O2	74.11
	143-07-7	3893	Dodecanoic acid	200.18	C12H24O2	74.32
	1673-08-1	74288	Hexadecanoic acid, cyclohexyl ester	338.32	C22H42O2	70.45
	5129-61-3	110444	Heptadecanoic acid, 16-methyl-, methyl ester	298.29	C19H38O2	82.05
	57-10-3	985	n-Hexadecanoic acid	256.24	C16H32O2	84.18
	57-11-4	5281	Octadecanoic acid	284.27	C18H36O2	71.32

Table S4. Cell viability (%) of the cytotoxic samples in the reporter gene assay experiments. PPARγ = peroxisome proliferator receptor gamma, GR = glucocorticoid receptor, SD = standard deviation.

**PPARγ CALUX**																		
mg plastic well^{-1}	PS 2			PP 3			PP 4			PVC 2			PLA1			PVC 4		
	mean	SD	n	mean	SD	n	mean	SD	n	mean	SD	n	mean	SD	n	mean	SD	n
1.5	**9.7**	**5**	16	**13.9**	**7.6**	12	**22.6**	**13.2**	16	**44.9**	**21.6**	20	**25.7**	**7.9**	**12**	107.9	14.9	20
0.75	**68.4**	**14.2**	16	**41**	**21**	12	**14.9**	**3.8**	16	106.8	15.4	20	103.2	15.1	12	120.9	20.3	20
0.38	108.2	10.6	16	86.9	20.8	12	**19.9**	**9.8**	16	117.5	16.3	20	111.6	15.5	12	126.7	20.4	20
0.19	120.5	7.1	16	106	16.6	12	97.7	25.4	16	123.9	19.9	20	118.4	21	12	126.6	30.5	20
0.09	121.7	6.3	16	123	20.4	12	109.8	11.8	16	125.5	25.1	20	128.6	24.5	12	121.8	34.6	20
0.04	116.8	15.7	16	104	26.4	12	101.8	10.9	16	115.4	34	20	131.9	21.5	12	101.8	27.2	20
**GR CALUX**																		
mg plastic well^{-1}	PS 2			PP 3			PP 4			PVC 2			PLA 1			PVC 4		
	mean	SD	n	mean	SD	n	mean	SD	n	mean	SD	n	mean	SD	n	mean	SD	n
1.5	**7**	**7.1**	12	**43.2**	**13**	12	**8.8**	**8.6**	16	**52**	**38.7**	16	**45.1**	**42.1**	12	**71**	**20**	12
0.75	**76.1**	**45.9**	12	**57.3**	**13.4**	12	**13**	**9.1**	16	109.8	10.6	16	104.3	9.3	12	96	9	12
0.38	101	38.4	12	88.9	15.3	12	**17.5**	**25.2**	16	118.3	7.6	16	112.7	6.9	12	110	10	12
0.19	117	23.7	12	110.4	5.8	12	96.8	18.4	16	117.2	8.5	16	112.2	6	12	116	6	12
0.09	113.3	19.6	12	115.2	7.5	12	118.4	17.8	16	118.9	8	16	113.9	6.9	12	113	12	12
0.04	111.2	31.7	12	103.8	10.4	12	105.8	13.4	16	104.5	6.8	16	107.5	9.6	12	102	11	12

Table S5. Comparison of the adipogenic effects of plastic extracts and the abundance of three metabolic disrupting chemicals detected in at least three samples, diphenyl phosphate (DPP), 2-ethylhexyl diphenyl phosphate (EHDP), and triphenyl phosphate (TPP) in the LC-QTOF-MS/MS.

	**Lipid droplet count**		**Raw abundance**													
Samples	Median	EC_{50}	DPP 1 [a]	DPP 2 [a]	DPP 3 [a]	DPP 4 [a]	EHDP	TPP 1 [a]	TPP 2 [a]	TPP 3 [a]	TPP 4 [a]	TPP 5 [a]	TPP 6 [a]	TPP 7 [a]	TPP 8 [a]	TPP 9 [a]
HDPE 1	164.6	3.1	0	0	<LOD	<LOD	0	0	0	1542	0	0	0	0	0	0
HDPE 2	161.4	3.1	0	0	<LOD	<LOD	0	0	0	0	<LOD	0	0	0	0	0
HDPE 3	360.8	3.1	0	0	<LOD	<LOD	0	0	0	0	0	0	0	2426	0	0
HDPE 4	369.1	3.1	0	0	<LOD	<LOD	4.7	0	0	0	<LOD	0	0	0	0	0
LDPE 1	447.3	3.1	0	0	<LOD	<LOD	5.7	0	0	0	<LOD	0	0	0	0	0
LDPE 2	373.2	3.1	0	0	<LOD	<LOD	0	0	0	24.6	0	0	0	0	0	137.4
LDPE 3	294.4	3.1	0	0	<LOD	<LOD	0	0	0	0	0	0	0	<LOD	0	0
LDPE 4	1352	1.59	0	0	<LOD	<LOD	0	0	0	0	974.2	0	0.1	0	0	0
PS 1	419.9	3.1	0	0	<LOD	0	0	14.6	0	0	0	0	0	0	0	0
PS 2	1333	2.05	9.8	0	<LOD	<LOD	0	0	16.7	0	0	0	0	0	<LOD	0
PS 3	186.1	3.1	0	0	<LOD	0	0	0	0	0	0	0	0	0	0	0
PS 4	123.7	3.1	0	0	0	<LOD	0	0	0	0	<LOD	0	0	0	0	0
PP 1	197.3	3.1	0	0	<LOD	<LOD	0	0	1633	0	<LOD	0	0	0	0	984.3
PP 2	413	3.1	0	0	0	<LOD	0	0	0	2.2	<LOD	0	0	0	0	0
PP 3	1742	1.4	0	0	<LOD	0	0	1184	0	59.3	<LOD	0	0	0	0	0
PP 4	2927	0.4	0	0	0	<LOD	0	0	0	0	<LOD	0	0	0	0	0
PP 5	119.1	3.1	0	0	0	<LOD	0	0	0	0	<LOD	0	0	0	0	0
PET 1	99.12	3.1	0	0	0	<LOD	0	0	0	0	<LOD	0	0	0	0	0
PET 2	185	3.1	0	0	0	<LOD	0	0	0	0	<LOD	0	0	0	0	0
PET 3	139.4	3.1	0	0	0	<LOD	0	0	0	0	<LOD	0	0	0	0	0
PET 4	122.4	3.1	0	0	<LOD	<LOD	0	0	0	0	0	0	0	0	0	0
PET 5	183.7	3.1	0	0	<LOD	<LOD	0	0	0	0	<LOD	0	0	<LOD	0	0

	Lipid droplet count		Raw abundance													
Samples	Median	EC_{50}	DPP 1 [a]	DPP 2 [a]	DPP 3 [a]	DPP 4 [a]	EHDP	TPP 1 [a]	TPP 2 [a]	TPP 3 [a]	TPP 4 [a]	TPP 5 [a]	TPP 6 [a]	TPP 7 [a]	TPP 8 [a]	TPP 9 [a]
PVC 1	171.4	3.1	0	0	0	<LOD	0	0	0	0	<LOD	0	0	0	0	0
PVC 2	3302	0.85	4251	2466	437,900	<LOD	0	0	0	356,178	<LOD	0	0	0	0	0
PVC 3	903.5	1	0	0	0	<LOD	0	0	0	0	554,290	0	27.1	0	0	0
PVC 4	3044	0.53	0	0	<LOD	315,591	2040	0	0	0	<LOD	4094	544,411	<LOD	4257	10.7
PUR 1	1340	0.61	0	0	0	<LOD	1.6	0	0	63.9	<LOD	771.1	0	0	0	0
PUR 2	969.4	0.33	0	0	0	<LOD	<LOD	0	0	0	<LOD	22.3	0	0	0	0
PUR 3	1186	0.32	0	0	<LOD	<LOD	65.8	95.7	1.4	31.3	0	0	0	0	0	0
PUR 4	2195	1.05	0	0	0	<LOD	438.7	0	0	0	<LOD	0	0.1	0	0	0
PLA 1	281.7	3.1	0	0	<LOD	<LOD	0	0	0	0	0	0	33.4	0	<LOD	0
PLA 2	203.7	3.1	0	0	<LOD	<LOD	0	0	0	0	0	0	0	<LOD	0	0
PLA 3	not analyzed via LC-QTOF-MS/MS															
PLA 4	271.8	3.1	0	0	<LOD	<LOD	0	0	0	0	<LOD	0	0	0	0	0

References

1. Zebisch, K.; Voigt, V.; Wabitsch, M.; Brandsch, M., Protocol for effective differentiation of 3T3-L1 cells to adipocytes. *Anal Biochem* **2012,** *425*, (1), 88-90.

2. Chappell, V. A.; Janesick, A.; Blumberg, B.; Fenton, S. E., Tetrabromobisphenol-A promotes early adipogenesis and lipogenesis in 3T3-L1 cells. *Toxicol Sci* **2018,** *166*, (2), 332-344.

3. Boucher, J. G.; Boudreau, A.; Ahmed, S.; Atlas, E., *In vitro* effects of Bisphenol A beta-D-glucuronide (BPA-G) on adipogenesis in human and murine preadipocytes. *Environ Health Perspect* **2015,** *123*, (12), 1287-93.

4. Kassotis, C. D.; Masse, L.; Kim, S.; Schlezinger, J. J.; Webster, T. F.; Stapleton, H. M., Characterization of adipogenic chemicals in three different cell culture systems: Implications for reproducibility based on cell source and handling. *Sci Rep* **2017,** *7*, 42104.

5. Sargis, R. M.; Johnson, D. N.; Choudhury, R. A.; Brady, M. J., Environmental endocrine disruptors promote adipogenesis in the 3T3-L1 cell line through glucocorticoid receptor activation. *Obesity* **2010,** *18*, (7), 1283-8.

6. Pomatto, V.; Cottone, E.; Cocci, P.; Mozzicafreddo, M.; Mosconi, G.; Nelson, E. R.; Palermo, F. A.; Bovolin, P., Plasticizers used in food-contact materials affect adipogenesis in 3T3-L1 cells. *J Steroid Biochem Mol Biol* **2018,** *178*, 322-332.

7. Riu, A.; Grimaldi, M.; le Maire, A.; Bey, G.; Phillips, K.; Boulahtouf, A.; Perdu, E.; Zalko, D.; Bourguet, W.; Balaguer, P., Peroxisome proliferator-activated receptor gamma is a target for halogenated analogs of bisphenol A. *Environ Health Perspect* **2011,** *119*, (9), 1227-32.

8. Kassotis, C. D.; Hoffman, K.; Volker, J.; Pu, Y.; Veiga-Lopez, A.; Kim, S. M.; Schlezinger, J. J.; Bovolin, P.; Cottone, E.; Saraceni, A.; Scandiffio, R.; Atlas, E.; Leingartner, K.; Krager, S.; Tischkau, S. A.; Ermler, S.; Legler, J.; Chappell, V. A.; Fenton, S. E.; Mesmar, F.; Bondesson, M.; Fernandez, M. F.; Stapleton, H. M., Reproducibility of adipogenic responses to metabolism disrupting chemicals in the 3T3-L1 pre-adipocyte model system: An interlaboratory study. *Toxicology* **2021,** *461*, 152900.

9. Adomshick, V.; Pu, Y.; Veiga-Lopez, A., Automated lipid droplet quantification system for phenotypic analysis of adipocytes using CellProfiler. *Toxicol Mech Methods* **2020,** *30*, (5), 378-387.

10. Zimmermann, L.; Dombrowski, A.; Volker, C.; Wagner, M., Are bioplastics and plant-based materials safer than conventional plastics? In vitro toxicity and chemical composition. *Environ Int* **2020,** *145*, 106066.

11. Zimmermann, L.; Bartosova, Z.; Braun, K.; Oehlmann, J.; Volker, C.; Wagner, M., Plastic products leach chemicals that induce *in vitro* toxicity under realistic use conditions. *Environ Sci Technol* **2021,** *55*, (17), 11814-11823.

12. Schymanski, E. L.; Singer, H. P.; Slobodnik, J.; Ipolyi, I. M.; Oswald, P.; Krauss, M.; Schulze, T.; Haglund, P.; Letzel, T.; Grosse, S.; Thomaidis, N. S.; Bletsou, A.; Zwiener, C.; Ibanez, M.; Portoles, T.; de Boer, R.; Reid, M. J.; Onghena, M.; Kunkel, U.; Schulz, W.; Guillon, A.; Noyon, N.; Leroy, G.; Bados, P.; Bogialli, S.; Stipanicev, D.; Rostkowski, P.; Hollender, J., Non-target screening with high-resolution mass spectrometry: critical review using a collaborative trial on water analysis. *Anal Bioanal Chem* **2015,** *407*, (21), 6237-55.

13. Amato, A. A.; Wheeler, H. B.; Blumberg, B., Obesity and endocrine-disrupting chemicals. *Endocr Connect* **2021,** *10*, (2), R87-R105.

14. Zimmermann, L.; Dierkes, G.; Ternes, T. A.; Volker, C.; Wagner, M., Benchmarking the *in vitro* toxicity and chemical composition of plastic consumer products. *Environ Sci Technol* **2019,** *53*, (19), 11467-11477.

15. Kassotis, C. D.; Hoffman, K.; Stapleton, H. M., Characterization of adipogenic activity of house dust extracts and semi-volatile indoor contaminants in 3T3-L1 cells. *Environ Sci Technol* **2017,** *51*, (15), 8735-8745.

16. Sun, W.; Duan, X.; Chen, H.; Zhang, L.; Sun, H., Adipogenic activity of 2-ethylhexyl diphenyl phosphate via peroxisome proliferator-activated receptor gamma pathway. *Sci Total Environ* **2020,** *711*, 134810.

17. Blanco, J.; Guardia-Escote, L.; Mulero, M.; Basaure, P.; Biosca-Brull, J.; Cabre, M.; Colomina, M. T.; Domingo, J. L.; Sanchez, D. J., Obesogenic effects of chlorpyrifos and its metabolites during the differentiation of 3T3-L1 preadipocytes. *Food Chem Toxicol* **2020,** *137*, 111171.

18. Sun, Z.; Cao, H.; Liu, Q. S.; Liang, Y.; Fiedler, H.; Zhang, J.; Zhou, Q.; Jiang, G., 4-Hexylphenol influences adipogenic differentiation and hepatic lipid accumulation *in vitro*. *Environ Pollut* **2021,** *268*, (Pt A), 115635.

19. Kassotis, C. D.; Kollitz, E. M.; Ferguson, P. L.; Stapleton, H. M., Nonionic ethoxylated surfactants induce adipogenesis in 3T3-L1 cells. *Toxicol Sci* **2018,** *162*, (1), 124-136.

20. Hao, C. J.; Cheng, X. J.; Xia, H. F.; Ma, X., The endocrine disruptor 4-nonylphenol promotes adipocyte differentiation and induces obesity in mice. *Cell Physiol Biochem* **2012,** *30*, (2), 382-94.

21. Janesick, A. S.; Dimastrogiovanni, G.; Vanek, L.; Boulos, C.; Chamorro-Garcia, R.; Tang, W.; Blumberg, B., On the utility of ToxCast and ToxPi as methods for identifying new obesogens. *Environ Health Perspect* **2016,** *124*, (8), 1214-26.

22. Andrews, F. V.; Kim, S. M.; Edwards, L.; Schlezinger, J. J., Identifying adipogenic chemicals: Disparate effects in 3T3-L1, OP9 and primary mesenchymal multipotent cell models. *Toxicol In Vitro* **2020,** *67*, 104904.

23. Bastos Sales, L.; Kamstra, J. H.; Cenijn, P. H.; van Rijt, L. S.; Hamers, T.; Legler, J., Effects of endocrine disrupting chemicals on *in vitro* global DNA methylation and adipocyte differentiation. *Toxicol In Vitro* **2013,** *27*, (6), 1634-43.

24. Yin, L.; Yu, K. S.; Lu, K.; Yu, X., Benzyl butyl phthalate promotes adipogenesis in 3T3-L1 preadipocytes: A High Content Cellomics and metabolomic analysis. *Toxicol In Vitro* **2016,** *32*, 297-309.

25. Wang, Y. F.; Chao, H. R.; Wu, C. H.; Tseng, C. H.; Kuo, Y. T.; Tsou, T. C., A recombinant peroxisome proliferator response element-driven luciferase assay for evaluation of potential environmental obesogens. *Biotechnol Lett* **2010,** *32*, (12), 1789-96.

26. Pereira-Fernandes, A.; Demaegdt, H.; Vandermeiren, K.; Hectors, T. L.; Jorens, P. G.; Blust, R.; Vanparys, C., Evaluation of a screening system for obesogenic compounds: screening of endocrine disrupting compounds and evaluation of the PPAR dependency of the effect. *PLoS One* **2013,** *8*, (10), e77481.

27. Wei, C.; Wang, X.; Yao, X.; Xi, F.; He, Y.; Xu, Y.; Ma, L.; Chen, X.; Zhao, C.; Du, R.; Pang, W.; Yang, G.; Yu, T. Y., Bifenthrin induces fat deposition by improving fatty acid uptake and inhibiting lipolysis in mice. *J Agric Food Chem* **2019,** *67*, (51), 14048-14055.

28. Martinez, M. A.; Blanco, J.; Rovira, J.; Kumar, V.; Domingo, J. L.; Schuhmacher, M., Bisphenol A analogues (BPS and BPF) present a greater obesogenic capacity in 3T3-L1 cell line. *Food Chem Toxicol* **2020,** *140*, 111298.

29. Choi, S. I.; Lee, J. S.; Lee, S.; Sim, W. S.; Kim, Y. C.; Lee, O. H., *Potentilla rugulosa* Nakai extract attenuates Bisphenol A-, S- and F-induced ROS production and differentiation of 3T3-L1 preadipocytes in the absence of dexamethasone. *Antioxidants (Basel)* **2020,** *9*, (2).

30. Drobna, Z.; Talarovicova, A.; Schrader, H. E.; Fennell, T. R.; Snyder, R. W.; Rissman, E. F., Bisphenol F has different effects on preadipocytes differentiation and weight gain in adult mice as compared with Bisphenol A and S. *Toxicology* **2019,** *420*, 66-72.

31. Chamorro-Garcia, R.; Kirchner, S.; Li, X.; Janesick, A.; Casey, S. C.; Chow, C.; Blumberg, B., Bisphenol A diglycidyl ether induces adipogenic differentiation of multipotent stromal stem cells through a peroxisome proliferator-activated receptor gamma-independent mechanism. *Environ Health Perspect* **2012,** *120*, (7), 984-9.

32. Helies-Toussaint, C.; Peyre, L.; Costanzo, C.; Chagnon, M. C.; Rahmani, R., Is bisphenol S a safe substitute for bisphenol A in terms of metabolic function? An *in vitro* study. *Toxicol Appl Pharmacol* **2014,** *280*, (2), 224-35.

33. Ariemma, F.; D'Esposito, V.; Liguoro, D.; Oriente, F.; Cabaro, S.; Liotti, A.; Cimmino, I.; Longo, M.; Beguinot, F.; Formisano, P.; Valentino, R., Low-dose Bisphenol-A impairs adipogenesis and generates dysfunctional 3T3-L1 adipocytes. *PLoS One* **2016,** *11*, (3), e0150762.

34. Hall, J. M.; Powell, H. A.; Rajic, L.; Korach, K. S., The role of dietary phytoestrogens and the nuclear receptor PPARgamma in adipogenesis: An *in vitro* study. *Environ Health Perspect* **2019,** *127*, (3), 37007.

35. Peshdary, V.; Calzadilla, G.; Landry, A.; Sorisky, A.; Atlas, E., Dechlorane Plus increases adipogenesis in 3T3-L1 and human primary preadipocytes independent of peroxisome proliferator-activated receptor gamma transcriptional activity. *Int J Obes (Lond)* **2019,** *43*, (3), 545-555.

36. Feige, J. N.; Gelman, L.; Rossi, D.; Zoete, V.; Metivier, R.; Tudor, C.; Anghel, S. I.; Grosdidier, A.; Lathion, C.; Engelborghs, Y.; Michielin, O.; Wahli, W.; Desvergne, B., The endocrine disruptor monoethyl-hexyl-phthalate is a selective peroxisome proliferator-activated receptor gamma modulator that promotes adipogenesis. *J Biol Chem* **2007,** *282*, (26), 19152-66.

37. Biserni, M.; Mesnage, R.; Ferro, R.; Wozniak, E.; Xenakis, T.; Mein, C. A.; Antoniou, M. N., Quizalofop-p-Ethyl induces adipogenesis in 3T3-L1 adipocytes. *Toxicol Sci* **2019,** *170*, (2), 452-461.

38. Mesnage, R.; Biserni, M.; Genkova, D.; Wesolowski, L.; Antoniou, M. N., Evaluation of neonicotinoid insecticides for oestrogenic, thyroidogenic and adipogenic activity reveals imidacloprid causes lipid accumulation. *J Appl Toxicol* **2018,** *38*, (12), 1483-1491.

39. Smith, A.; Yu, X.; Yin, L., Diazinon exposure activated transcriptional factors CCAAT-enhancer-binding proteins alpha (C/EBPalpha) and peroxisome proliferator-activated receptor gamma (PPARgamma) and induced adipogenesis in 3T3-L1 preadipocytes. *Pestic Biochem Physiol* **2018,** *150*, 48-58.

40. Temkin, A. M.; Bowers, R. R.; Magaletta, M. E.; Holshouser, S.; Maggi, A.; Ciana, P.; Guillette, L. J.; Bowden, J. A.; Kucklick, J. R.; Baatz, J. E.; Spyropoulos, D. D., Effects of crude oil/dispersant mixture and dispersant components on PPARgamma activity *in vitro* and *in vivo*: Identification of Dioctyl Sodium Sulfosuccinate (DOSS; CAS #577-11-7) as a probable obesogen. *Environ Health Perspect* **2016,** *124*, (1), 112-9.

41. Bowers, R. R.; Temkin, A. M.; Guillette, L. J.; Baatz, J. E.; Spyropoulos, D. D., The commonly used nonionic surfactant Span 80 has RXRalpha transactivation activity, which likely increases the obesogenic potential of oil dispersants and food emulsifiers. *Gen Comp Endocrinol* **2016,** *238*, 61-68.

42. Cano-Sancho, G.; Smith, A.; La Merrill, M. A., Triphenyl phosphate enhances adipogenic differentiation, glucose uptake and lipolysis via endocrine and noradrenergic mechanisms. *Toxicol In Vitro* **2017,** *40*, 280-288.

43. Xie, X.; Yu, C.; Ren, Q.; Wen, Q.; Zhao, C.; Tang, Y.; Du, Y., Exposure to HBCD promotes adipogenesis both *in vitro* and *in vivo* by interfering with Wnt6 expression. *Sci Total Environ* **2020,** *705*, 135917.

44. Tung, E. W. Y.; Ahmed, S.; Peshdary, V.; Atlas, E., Firemaster(R) 550 and its components isopropylated triphenyl phosphate and triphenyl phosphate enhance adipogenesis and transcriptional activity of peroxisome proliferator activated receptor (PPARgamma) on the adipocyte protein 2 (aP2) promoter. *PLoS One* **2017,** *12*, (4), e0175855.

45. Elmore, S. E.; Cano-Sancho, G.; La Merrill, M. A., Disruption of normal adipocyte development and function by methyl- and propyl- paraben exposure. *Toxicol Lett* **2020,** *334*, 27-35.

46. Hao, C.; Cheng, X.; Xia, H.; Ma, X., The endocrine disruptor mono-(2-ethylhexyl) phthalate promotes adipocyte differentiation and induces obesity in mice. *Biosci Rep* **2012,** *32*, (6), 619-29.

47. Wen, Q.; Xie, X.; Zhao, C.; Ren, Q.; Zhang, X.; Wei, D.; Emanuelli, B.; Du, Y., The brominated flame retardant PBDE 99 promotes adipogenesis via regulating mitotic clonal expansion and PPARgamma expression. *Sci Total Environ* **2019,** *670*, 67-77.

48. Watkins, A. M.; Wood, C. R.; Lin, M. T.; Abbott, B. D., The effects of perfluorinated chemicals on adipocyte differentiation *in vitro*. *Mol Cell Endocrinol* **2015,** *400*, (C), 90-101.

49. Ma, Y.; Yang, J.; Wan, Y.; Peng, Y.; Ding, S.; Li, Y.; Xu, B.; Chen, X.; Xia, W.; Ke, Y.; Xu, S., Low-level perfluorooctanoic acid enhances 3 T3-L1 preadipocyte differentiation via altering peroxisome proliferator activated receptor gamma expression and its promoter DNA methylation. *J Appl Toxicol* **2018,** *38*, (3), 398-407.

50. Neel, B. A.; Brady, M. J.; Sargis, R. M., The endocrine disrupting chemical tolylfluanid alters adipocyte metabolism via glucocorticoid receptor activation. *Mol Endocrinol* **2013,** *27*, (3), 394-406.

51. Pereira-Fernandes, A.; Vanparys, C.; Hectors, T. L.; Vergauwen, L.; Knapen, D.; Jorens, P. G.; Blust, R., Unraveling the mode of action of an obesogen: mechanistic analysis of the model obesogen tributyltin in the 3T3-L1 cell line. *Mol Cell Endocrinol* **2013,** *370*, (1-2), 52-64.

52. Grun, F.; Watanabe, H.; Zamanian, Z.; Maeda, L.; Arima, K.; Cubacha, R.; Gardiner, D. M.; Kanno, J.; Iguchi, T.; Blumberg, B., Endocrine-disrupting organotin compounds are potent inducers of adipogenesis in vertebrates. *Mol Endocrinol* **2006,** *20*, (9), 2141-55.

53. Inadera, H.; Shimomura, A., Environmental chemical tributyltin augments adipocyte differentiation. *Toxicol Lett* **2005,** *159*, (3), 226-34.

54. Kim, S.; Li, A.; Monti, S.; Schlezinger, J. J., Tributyltin induces a transcriptional response without a brite adipocyte signature in adipocyte models. *Arch Toxicol* **2018,** *92*, (9), 2859-2874.

55. Li, X.; Ycaza, J.; Blumberg, B., The environmental obesogen tributyltin chloride acts via peroxisome proliferator activated receptor gamma to induce adipogenesis in murine 3T3-L1 preadipocytes. *J Steroid Biochem Mol Biol* **2011,** *127*, (1-2), 9-15.

56. Regnier, S. M.; El-Hashani, E.; Kamau, W.; Zhang, X.; Massad, N. L.; Sargis, R. M., Tributyltin differentially promotes development of a phenotypically distinct adipocyte. *Obesity* **2015,** *23*, (9), 1864-71.

57. Dong, M.; Yuan, P.; Song, Y.; Lei, H.; Chen, G.; Zhu, X.; Wu, F.; Chen, C.; Liu, C.; Shi, Z.; Zhang, L., *In vitro* effects of Triclocarban on adipogenesis in murine preadipocyte and human hepatocyte. *J Hazard Mater* **2020,** *399*, 122829.

58. Li, X.; Pham, H. T.; Janesick, A. S.; Blumberg, B., Triflumizole is an obesogen in mice that acts through peroxisome proliferator activated receptor gamma (PPARgamma). *Environ Health Perspect* **2012,** *120*, (12), 1720-6.

59. Kim, S.; Rabhi, N.; Blum, B. C.; Hekman, R.; Wynne, K.; Emili, A.; Farmer, S.; Schlezinger, J. J., Triphenyl phosphate is a selective PPARgamma modulator that does not induce brite adipogenesis *in vitro* and *in vivo*. *Arch Toxicol* **2020,** *94*, (9), 3087-3103.
